# Novel Bioactive Penicipyrroether A and Pyrrospirone J from the Marine-Derived *Penicillium* sp. ZZ380

**DOI:** 10.3390/md17050292

**Published:** 2019-05-15

**Authors:** Tengfei Song, Mingmin Tang, Hengju Ge, Mengxuan Chen, Xiaoyuan Lian, Zhizhen Zhang

**Affiliations:** 1 College of Pharmaceutical Sciences, Zhejiang University, Hangzhou 310058, China; tmm0907@163.com; 2 Ocean College, Zhoushan Campus, Zhejiang University, Zhoushan 316021, China; sdasheng72@163.com (T.S.); 15805152141@163.com (H.G.); zerocmx@163.com (M.C.)

**Keywords:** marine fungus, *Penicillium* sp. ZZ380, penicipyrroether A, pyrrospirone J, antiproliferative and antibacterial activities

## Abstract

The marine-sourced fungus *Penicillium* sp. ZZ380 was previously reported to have the ability to produce a series of new pyrrospirone alkaloids. Further investigation on this strain resulted in the isolation and identification of novel penicipyrroether A and pyrrospirone J. Each of them represents the first example of its structural type, with a unique 6/5/6/5 polycyclic fusion that is different from the 6/5/6/6 fused ring system for the reported pyrrospirones. Their structures were elucidated by extensive nuclear magnetic resonance (NMR) and high resolution electrospray ionization mass spectroscopy (HRESIMS) spectroscopic analyses, electronic circular dichroism (ECD) and ^13^C NMR calculations and X-ray single crystal diffraction. Penicipyrroether A showed potent antiproliferative activity against human glioma U87MG and U251 cells with half maximal inhibitory concentration (IC_50_) values of 1.64–5.50 μM and antibacterial inhibitory activity with minimum inhibitory concentration (MIC) values of 1.7 μg/mL against methicillin-resistant *Staphylococcus aureus* and 3.0 μg/mL against *Escherichia coli*.

## 1. Introduction

Hirsutellones and related compounds are a family of fungal secondary metabolites that bear a unique 13-membered ether ring, which is composed of the structural units of decahydrofluorene, *para*-cyclophane and pyrrolidinone [1,2]. This family of fungal metabolites is divided into four groups: GKK1032s, hirsutellones, pyrrocidines and pyrrospirones. To date, about 29 such metabolites have been isolated and identified from fungal sources of genera *Cylindrocarpon*, *Embellisia*, *Hirsutella*, *Lewia*, *Neonectria*, *Penicillium* and *Trichoderma* and some of these metabolites have proved to have cytotoxic, antifungal and antibacterial activities [1,2,3,4].

**Figure 1:**
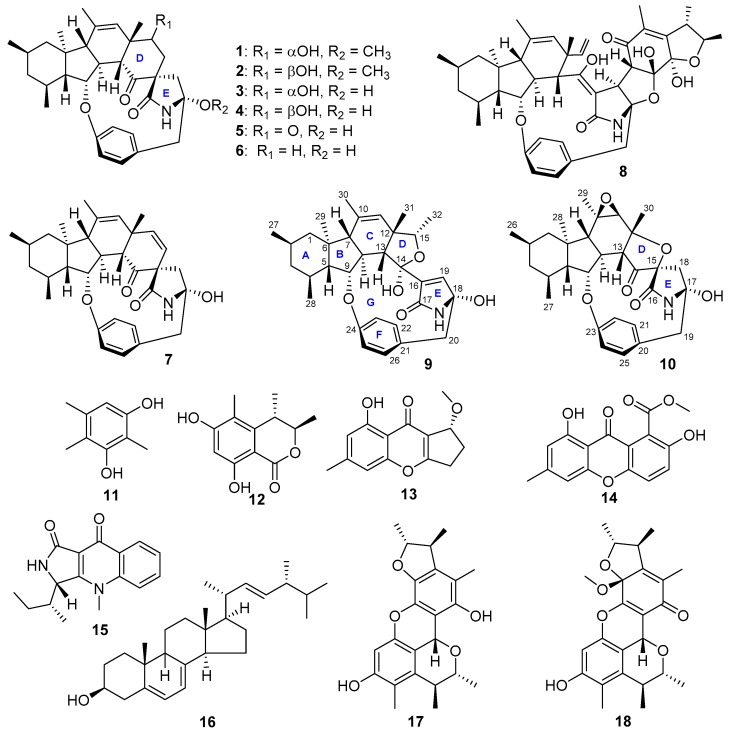
Structures of compounds **1**–**18** isolated from the cultures of *Penicillium* sp. ZZ380.

A marine fungus *Penicillium* sp. ZZ380 [2] was recently isolated from a wild sea crab *Pachygrapsus crassipes* during the course of our ongoing program to discover novel bioactive agents from marine microorganisms. This fungus in the BMPM liquid medium produced new pyrrospirones C–I (**1**–**7**) [2], penicipyrrodiether A (**8**) [3] and compound **10** (Figure 1), the structure of which was not possible to establish previously, due to its structural instability. Pyrrospirone G (**5**) was found to have potent activity against the proliferation of different glioma cells and pyrrospirones C (**1**), F (**4**), I (**7**) and penicipyrrodiether A (**8**) showed activities in inhibiting the growth of methicillin-resistant *Staphylococcus aureus* (MRSA) and *Escherichia coli*.

It is well known that OSMAC (One Strain, Many Compounds) has been used to activate the silent gene cluster of microbial secondary metabolic biosynthesis to express and then produce different novel bioactive natural products [5,6,7]. In order to obtain more bioactive compounds from this pyrrospirones-produced fungus ZZ380, a different liquid medium of PDB (potato dextrose broth) was used to culture strain ZZ380 in this study, resulting in the isolation of nine compounds, including novel penicipyrroether A (**9**) (Figure 1) but no pyrrospirones and penicipyrrodiether A. Herein, we report the culture of strain ZZ380 in PDB medium as well as the structural determination and bioactive evaluation of penicipyrroether A (**9**) and the previously unidentified compound **10**.

## 2. Results and Discussion

An EtOAc extract prepared from a mass culture of *Penicillium* sp. ZZ380 in PDB medium was separated by ODS column chromatography, followed by HPLC purification, to give compounds **9** and **11**–**18**. Compound **10** was isolated from a previous culture of ZZ380 in BMPM medium [2]. On the basis of the nuclear magnetic resonance (NMR) data and specific rotation, as well as the comparison with the reported data, eight known compounds were identified as 2,4,5-trimethylresorcinol (**11**) [8], stoloniferol B (**12**) [9], coniochaetone E (**13**) [10], pinselin (**14**) [11], quinolactacin A_1_ (**15**) [12], ergosterol (**16**) [13], penicitrinol A (**17**) [14] and B (**18**) [15]. Their ^13^C NMR data are presented in Tables S1 and S2 (Supplementary Materials).

**Figure 2:**
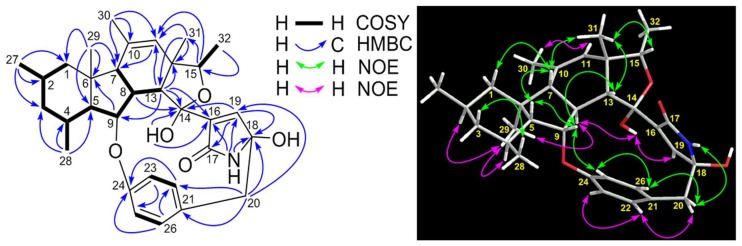
^1^H-^1^H COSY, key HMBC and NOE correlations of penicipyrroether A (**9**).

**Figure 3:**
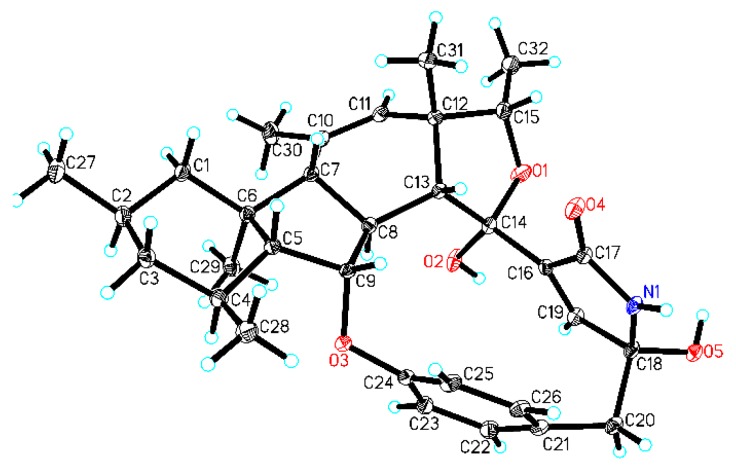
X-ray crystal structure of penicipyrroether A (**9**, Cu Kα radiation).

Compound **9** was obtained as colorless block crystals from MeOH and has a molecular formula C_32_H_41_NO_5_ deduced from its high resolution electrospray ionization mass spectroscopy (HRESIMS) ions at *m*/*z* [M + H]^+^ 520.3063 and [M + Na]^+^ 542.2877 as well as its ^13^C NMR data. Analyses of its ^1^H, ^13^C, ^1^H-^1^H COSY, HSQC and HMBC spectra (Figures S1–S17) indicated that the structural parts of rings A–C, F and G for **9** and pyrrospirones C–I (**1**–**7**) are the same. However, the structures of **9** and pyrrospirones have significant differences. The first difference is the ring D with a five-membered ether ring for **9** and a cyclohexanone for pyrrospirones. The second difference is that **9** has no spiro junction for rings D and E. The five-membered ether ring D and its connections with rings C and E were confirmed by ^1^H-^1^H COSY correlation (Table 1 and Figure 2) of H-8 (*δ*_H_ 3.12, m) with H-13 (*δ*_H_ 3.78, d, *J* = 5.7 Hz) and HMBC correlations (Table 1 and Figure 2) of H-13 with C-8 (*δ*_C_ 48.9), C-9 (*δ*_C_ 87.5), C-11 (*δ*_C_ 126.8), C-12 (*δ*_C_ 48.6), C-14 (*δ*_C_ 102.1), C-16 (*δ*_C_ 139.2) and C-31 (*δ*_C_ 22.0), H-15 (*δ*_H_ 4.35, q, *J* = 6.4 Hz) with C-11 and C-31 and H-19 (*δ*_H_ 6.95, d, *J* = 1.8 Hz) with C-14, C-17 (*δ*_C_ 172.7) and C-18 (*δ*_C_ 88.1). The locations of CH_3_-31, CH_3_-32 and OH-14 were also established by HMBC correlations of H_3_-31 (*δ*_H_ 1.35, s) with C-11, C-12, C-13 (*δ*_C_ 56.3) and C-15 (*δ*_C_ 79.4) and H_3_-32 (*δ*_H_ 1.26, d, *J* = 6.4 Hz) with C-12 and C-15, as well as OH-14 (*δ*_H_ 6.25, s) with C-14 and C-16. The planner structure of **9** was further confirmed by X-ray diffraction analysis (Cu Kα, CCDC deposition number 1868814, crystallized in MeOH, Figure 3).

**Figure 4:**
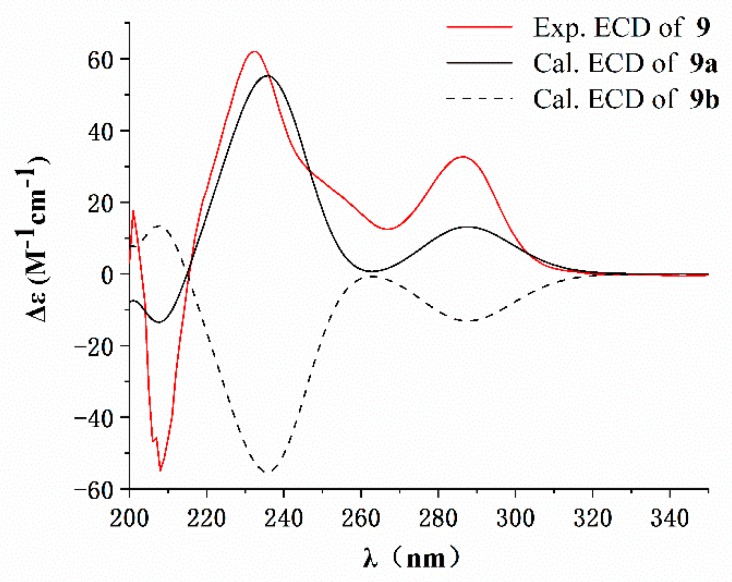
Experimental electronic circular dichroism (ECD) spectrum of penicipyrroether A (**9**, 200–350 nm) in MeOH and the calculated ECD spectra of the model molecules **9a** and **9b** at the B3LYP/6-311+G(d,p) level (**9a**: 2*R*,4*S*,5*R*,6*S*,7*S*,8*S*,9*S*,12*S*,13*R*,14*R*,18*R;* **9b**: 2*S*,4*R*,5*S*,6*R*,7*R*,8*R*,9*R*,12*R*,13*S*,14*S*,18*S*).

The relative stereochemistry of **9** was established based on the analyses of NOESY spectrum (Figures S18 and S19) and coupling constants. As depicted in Figure 2, NOE correlations of H-9 with H-5 and H-13, H_3_-31 with H-7, H-13 and H-15, βH-20 with NH-17 and H-26 and H-25 with H-9 and H-26 indicated a β-orientation for these protons. Similarly, NOE correlations of H_3_-29 with H-2, H-4 and H-8, H-8 with OH-14 and H-22 with αH-20 and H-23 were suggestive of an α-orientation for these protons. The large coupling constants of ^3^*J*_4_,_5_ (11.3 Hz) and ^3^*J*_7_,_8_ (13.2 Hz) also confirmed the *trans*-juncture for A/B and B/C rings [16]. NOE correlations of H-11 with H_3_-30 and H-19 with OH-14 indicated a *Z*-geometry for both of the double bonds at C_10–11_ and C_16–19_. The absolute configuration of **9** was determined as 2*R*,4*S*,5*R*,6*S*,7*S*,8*S*,9*S*,12*S*,13*R*,14*R*,18*R* based on the result of the X-ray diffraction analysis (Cu Kα, Flack parameter 0.02). A computational method was also applied to assign the absolute stereochemistry of **9** by comparing its experimental electronic circular dichroism (ECD) spectrum with the calculated ECD spectra. The result (Figure 4) showed that the calculated ECD spectrum of model molecule **9a** was close to the experimental curve of **9**. Based on the foregoing evidence, the structure of **9** was elucidated as a new alkaloid, named penicipyrroether A. Its ^13^C and ^1^H NMR data (Table 1) were assigned based on a combination of HSQC, ^1^H-^1^H COSY, HMBC and NOE spectroscopic analyses.

**Figure 5:**
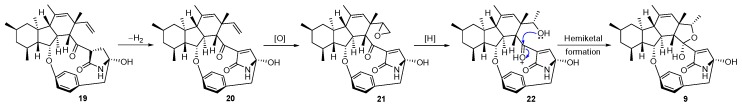
Plausible biosynthetic pathway of penicipyrroether A (**9**).

It has been reported that compound **20**, a dehydro-derivative of GKK1032A_2_ (**19**) (Figure 5), might be a precursor of the fungal metabolites of GKK1032s, pyrrospirones and penicipyrrodiether A [1,2,3,17]. Similarly, penicipyrroether A (**9**) might be also derived from **20**. A proposed biosynthetic pathway for penicipyrroether A (**9**) was depicted in Figure 5. After oxidation of the vinyl group of **20**, the epoxide derivative (**21**) was transformed into **22** and then an intramolecular hemiketal formation [3,15] of **22** forms penicipyrroether A (**9**).

**Figure 6:**
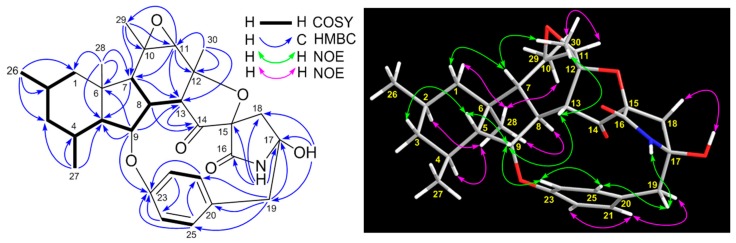
^1^H-^1^H COSY, key HMBC and NOE correlations of pyrrospirone J (**10**).

Compound **10** was obtained as a colorless amorphous powder and has a molecular formula C_30_H_37_NO_6_ deduced from its HRESIMS ions at *m*/*z* [M + H]^+^ 508.2711 and [M + Na]^+^ 530.2531 as well as its ^13^C NMR data, which showed two fewer carbons, when compared to the structure of penicipyrroether A (**9**). Detailed interpretation of ^1^H, ^13^C, ^1^H-^1^H COSY, HSQC and HMBC spectra (Figures S23–S38) demonstrated that compound **10** is an analogue of penicipyrroether A (**9**) with the same structural part of rings A, B, F and G but possesses an epoxy moiety at C-10 and C-11 and a different five-membered ether ring D fused with ring E through a spiro carbon of C-15. In addition, the dehydro-pyrrolidinone moiety (ring E) in **9** was replaced by a pyrrolidinone moiety in **10**. The presence of the epoxy moiety was confirmed by HMBC correlations (Table 2 and Figure 6) of H-7 (*δ*_H_ 1.55, d, *J* = 14.3 Hz) with C-10 (*δ*_C_ 58.8) and C-11 (*δ*_C_ 63.5), H-11 (*δ*_H_ 2.46, s) with C-10, C-12 (*δ*_C_ 81.3), C-13 (*δ*_C_ 44.5), C-29 (*δ*_C_ 21.0) and C-30 (*δ*_C_ 26.0) and H_3_-29 (*δ*_H_ 1.20, s) with C-7 (*δ*_C_ 49.9), C-10 and C-11. Similarly, the ether ring D and its connections with rings C and E was indicated by HMBC correlations of H-8 (*δ*_H_ 2.79, m) with C-13 and C-14 (*δ*_C_ 180.9), H-11 with C-12 and C-13, H-13 (*δ*_H_ 3.21, d, *J* = 8.0 Hz) with C-8 (*δ*_C_ 39.2), C-11, C-12, C-14 and C-30, H-18 (*δ*_H_ 1.99, 2.45, d, *J* = 12.1Hz, each) with C-14, C-15 (*δ*_C_ 79.7), C-16 (*δ*_C_ 172.1), C-17 (*δ*_C_ 86.2) and C-19 (*δ*_C_ 45.0), H_3_-30 (*δ*_H_ 1.59, s) with C-11, C-12 and C-13 and NH-16 (*δ*_H_ 8.76, s) with C-15, C-16, C-17 and C-18 (*δ*_C_ 40.9).

**Figure 7:**
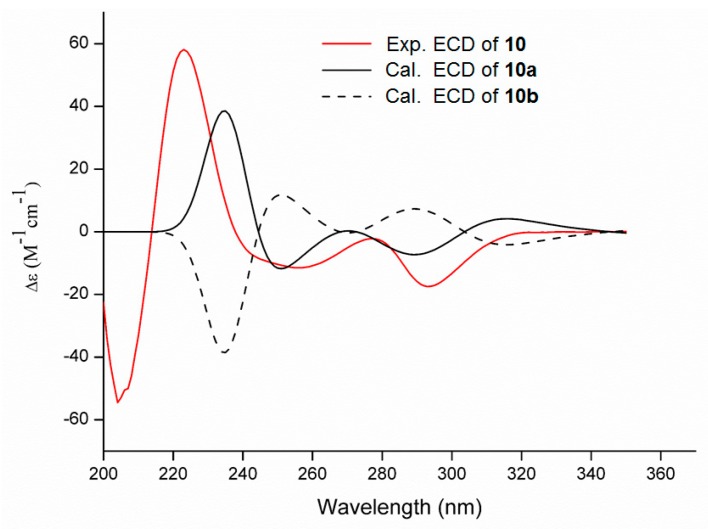
Experimental ECD spectrum of pyrrospirone J (**10**, 200–400 nm) in MeOH and the calculated ECD spectra of the model molecules **10a** and **10b** at the B3LYP/6-311+G(d,p) level (**10a**: 2*R*,4*S*,5*R*,6*R*,7*R*,8*S*,9*S*,10*R*,11*R*,12*R*,13*R*,15*R*,17*R*; **10b**: 2*S*,4*R*,5*S*,6*S*,7*S*,8*R*,9*R*,10*S*,11*S*,12*S*,13*S*,15*S*,17*S*).

The relative stereochemistry of **10** was assigned by analyses of the NOESY spectrum (Figures S39 and S40) and coupling constants. NOE correlations (Figure 6) of H-5 (*δ*_H_ 1.22) with βH-3 (*δ*_H_ 0.51) and H-9 (*δ*_H_ 4.82), H-7 (*δ*_H_ 1.55) with βH-1 (*δ*_H_ 0.81) and H_3_-30 (*δ*_H_ 1.59), H-9 with H-13 (*δ*_H_ 3.21) and H-24 (*δ*_H_ 6.94), H-13 with H_3_-30, NH-16 (*δ*_H_ 8.76) with βH-19 (*δ*_H_ 2.99) and H-25 (*δ*_H_ 6.78) and H-25 with βH-19 and H-24 suggested a β-orientation for these protons; while NOE correlations of H-8 (*δ*_H_ 2.79) with H_3_-28 (*δ*_H_ 1.05), H_3_-28 with αH-1 (*δ*_H_ 1.80), H-2 (*δ*_H_ 1.82), H-4 (*δ*_H_ 1.78) and H_3_-29 (*δ*_H_ 1.20), H_3_-29 with H-11 (*δ*_H_ 2.46), H-21 (*δ*_H_ 6.92) with αH-19 (*δ*_H_ 2.69) and H-22 (*δ*_H_ 6.82) and H-18 (*δ*_H_ 1.99) with OH-17 (*δ*_H_ 6.24) proved an α-orientation for these protons. The *trans*-juncture for A/B and B/C rings was also confirmed by the large coupling constants of ^3^*J*_4_,_5_ (11.6 Hz) and ^3^*J*_7_,_8_ (14.3 Hz) [16]. The absolute configuration of **10** was determined as 2*R*,4*S*,5*R*,6*R*,7*R*,8*S*,9*S*,10*R*,11*R*,12*R*,13*R*,15*R*,17*R* based on the result from ECD calculation (Figure 7). Unfortunately, several efforts to obtain single crystal for X-ray diffraction were not successful because the structure of **10** was changed during the process of crystallization.

**Figure 8:**
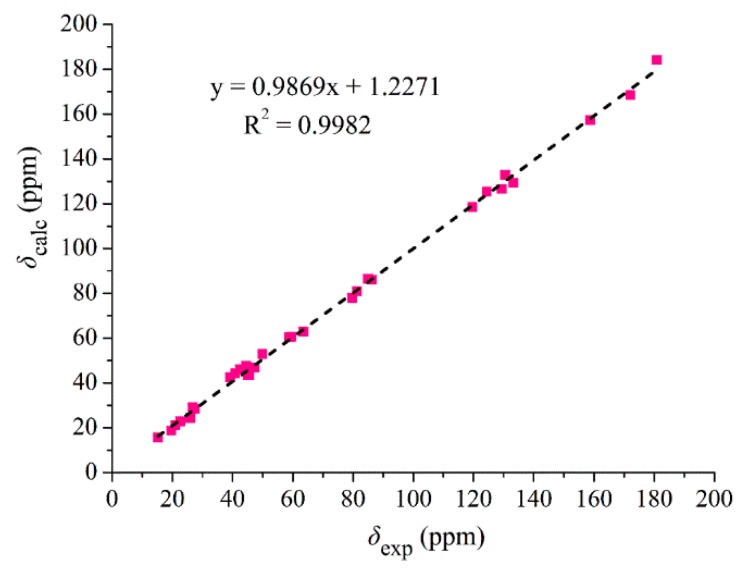
Regression analysis of experimental versus calculated ^13^C NMR chemical shifts of pyrrospirone J (**10**) at the mPW1PW91-SCRF (DMSO)/6-311+g(d,p) level and linear fitting is shown as a line.

It was noted that the ketonic carbonyl at C-14 of **10** resonated at 180.9 ppm, which is unusual. In order to confirm this possibility, we conducted a computational calculation of its ^13^C chemical shifts [18,19,20]. The calculated ^13^C NMR data (Table S7) of **10** were in good agreement with its experimental values, with the corrected mean absolute error (CMAE) of 0.28 ppm and the correlation coefficient (*R*^2^) of 0.9982 (Figure 8) and the calculated ^13^C chemical shift for C-14 was 184.1 ppm, which was close to the experimental value of 180.9 ppm. Based on the foregoing evidence, the structure of **10** was determined as a new alkaloid, named pyrrospirone J. The full assignment of ^13^C and ^1^H NMR data (Table 2) was made based on HSQC, ^1^H-^1^H COSY, HMBC and NOE spectroscopic analyses.

New alkaloids—penicipyrroether A (**9**) and pyrrospirone J (**10**)—were evaluated for their activities in inhibiting the proliferation of human glioma U87MG and U251 cells through sulforhodamine B (SRB) assay [21]. Doxorubicin (DOX, a chemotherapeutic drug) was used as a positive control. It has been found that both compounds had activity against different glioma cells with IC_50_ values of 1.64–5.50 μM for **9** and 10.52–17.92 μM for **10** (Table 3). The cytotoxicities of penicipyrroether A (**9**) and DOX against normal human astrocytes were also tested and showed CC_50_ values of 23.28 ± 1.05 μM for **9** and 8.57 ± 0.16 μM for DOX. The data indicated that the antiglioma activity of **9** was equivalent to (or slightly stronger than) the activity of the positive control DOX and the selective index (CC_50_/IC_50_) of 4.2–14.2 for **9** was higher than that of 1.1–7.1 for DOX.

The antibacterial activities of penicipyrroether A (**9**) and pyrrospirone J (**10**) against MRSA and *E*. *coli* were also determined by the micro broth dilution method [22]. Gentamicin (an antibiotic against both Gram-positive and Gram-negative bacteria) and vancomycin (an antibiotic against MRSA) were used as positive controls. The results (Table 3) showed that penicipyrroether A (**9**) had good antibacterial activities with MIC values of 1.7 μg/mL against MRSA and 3.0 μg/mL against *E. coli*. However, pyrrospirone J (**10**) was inactive at a concentration of 50 μg/mL.

## 3. Materials and Methods

### 3.1. General Experimental Procedures

Optical rotation, ultraviolet-visible (UV) and electronic circular dichroism (ECD) were measured on an Autopol I polarimeter (Rudolph Research Analytical, Hackettstown, NJ, USA), a METASH UV-8000 spectrometer (Shanghai METASH Instruments Co. Ltd., Shanghai, China) and a JASCO J-815 spectropolarimeter (JASCO Co. Tokyo, Japan), respectively. Infrared radiation (IR) spectra were recorded on a Bruker TENSOR II high performance FT-IR spectrometer (Bruker, Karlsruhe, Germany). HRESIMS data was obtained from an Agilent 6230 time-of-flight liquid chromatography–mass spectrometry (TOF LC-MS, Agilent, CA, USA). NMR spectra were acquired on a Bruker 500 spectrometer and chemical shifts were expressed in *δ* (ppm). Octadecyl silane (ODS, Cosmosil 75C_18_-Prep, Nacalai Tesque Inc., Kyoto, Japan) was applied for column chromatography. HPLC separation was conducted on a SHIMADZU LC-20AP prepared HPLC system with column A (Welch-20, 250 × 21 mm, 5 μm, XB-C_18_) or column B (CT-30, 280 × 30 mm, 10 μm, Fuji-C_18_). All solvents were ordered from the Sinopharm Chemical Reagent Co. Ltd. (Shanghai, China). Human glioma U87MG (JDS-2568) and U251 (XB-0439) cells were purchased from the Cell Bank of the Chinese Academy of Sciences and normal human astrocytes (HA, Cat. No. 1800) from the ScienCell. Methicillin-resistant *Staphylococcus aureus* (MRSA) ATCC 43300 and *Escherichia coli* ATCC 25922 were gifts from Drs. Zhongjun Ma and Pinmei Wang, respectively. Gentamicin (99.6%) and vancomycin (>98.0%) were bought from Meilune Biotechnology Co. Ltd. (Dalian, China), doxorubicin (DOX, >98.0%) from Sigma-Aldrich, PDA (potato dextrose agar) from Baisi Biotechnology Co. Ltd. (Hangzhou, China) and MHB (Mueller-Hinton Broth) from Oxoid Ltd. PDB medium (potato dextrose broth, potato 100 g, glucose 10 g, sea salt 35 g, tap water 1000 mL) was made in the authors’ laboratory.

### 3.2. Marine Strain ZZ380

Strain ZZ380 was previously isolated from marine crab *Pachygrapsus crassipes* and assigned as *Penicillium* sp. ZZ380 by ITS DNA sequence analysis [2].

### 3.3. Mass Culture of Strain ZZ380

Colonies of the strain ZZ380 from the PDA slant were inoculated into 250 mL of PDB liquid medium in a 500 mL Erlenmeyer flask and then incubated at 28 °C for three days on a rotary shaker (180 rpm) to produce seed broth. The seed broth of 5 mL was inoculated into a 500 mL Erlenmeyer flask, containing 250 mL of PDB medium and then incubated at 28 °C for 30 days under stationary state. A total of 55 L of culture were prepared for this study.

### 3.4. Isolation of Compounds 9–18

The 55 L culture of ZZ380 in PDB medium was centrifuged to give mycelia and broth. The mycelia were extracted with MeOH three times to get a MeOH extract. The broth was partitioned with EtOAc three times to give an EtOAc extract. A mixture (23.0 g) of the MeOH and EtOAc extract was fractionated by an ODS column eluting successively with 40%, 60%, 80% and 100% MeOH to afford four fractions (Frs. 1–4). Fr. 1 was further separated by an ODS column eluting with 35%, 45% and 55% MeOH to give three sub fractions (SFrs. 1a–1c). Through purification using prepared HPLC with column A at a flow rate of 12 mL/min, compounds **11** (4.2 mg, t_R_: 22.0 min, mobile phase: MeOH/H_2_O, 38/62, detector: 254 nm) and **12** (6.7 mg, t_R_: 31.0 min, mobile phase: MeOH/H_2_O, 38/62, detector: 254 nm) were obtained from SFr. 1a; compounds **15** (4.0 mg, t_R_: 33.0 min, mobile phase: MeOH/H_2_O, 45/55, detector: 210 nm), **14** (20.5 mg, t_R_: 37.0 min, mobile phase: MeOH/H_2_O, 53/47, detector: 254 nm), **18** (7.0 mg, t_R_: 37.0 min, mobile phase: MeOH/H_2_O, 66/34, detector: 254 nm) were obtained from SFr. 1b, SFr. 1c and Fr. 2, respectively. Fr. 3 and Fr. 4 were also separated by ODS column eluting with 85% or 95% MeOH to furnish SFrs. 3a and 3b or SFrs. 4a and 4b. Through purification using prepared HPLC with column B at a flow rate of 15 mL/min, compounds **13** (43.0 mg, t_R_: 19.0 min, mobile phase: MeOH/H_2_O, 80/20, detector: 210 nm) and **17** (9.5 mg, t_R_: 45.0 min, mobile phase: MeOH/H_2_O, 83/17, detector: 254 nm) were obtained from SFrs. 3a and 3b, respectively; while compounds **9** (3.1 mg, t_R_: 41.0 min, mobile phase: MeOH/H_2_O, 91/9, detector: 210 nm) and **16** (50.0 mg, t_R_: 62.0 min, mobile phase: MeOH/H_2_O, 98/2; 280 nm; t_R_ 62 min; 50.0 mg) were obtained from SFrs. 4a and 4b, respectively.

Compound **10** was isolated from a previous culture of strain ZZ380 in BMPM medium [2]. A crude extract was fractionated on an ODS column eluting successively with 80% MeOH, 90% MeOH and 100% MeOH to give six fractions (Frs. 1–6) based on the results of TLC analysis. Fr. 2 was re-separated by an ODS column eluting with 80% MeOH to give Frs. 2A and 2B. Fr. 2A was further separated by prepared HPLC with column B using mobile phase of acetonitrile/water (68:32) at a flow rate of 10 mL/min to give **10** (2.0 mg, t_R_: 60.0 min) and Frs. 2A_1_–2A_3_.

Penicipyrroether A (**9**): Colorless block crystals; molecular formula C_32_H_41_NO_5_; [*α*]${}_{D}^{20}$ + 71.4° (*c* 0.10, MeOH); ECD (10 mg/L, MeOH) λ_max_ (Δε) 208 (−57.22), 233 (+64.65), 286 (+34.08) nm; UV (MeOH) λ_max_ (log ε) 202 (4.20), 229 (3.66), 278 (2.67) nm; IR (MeOH) *ν*_max_ 3375, 2924, 1672, 1437, 1408, 1239, 1098, 1013, 951 cm^−1^; ^13^C (125 MHz) and ^1^H (500 MHz) NMR data, see Table 1; HRESIMS *m*/*z* [M + H]^+^ 520.3063 (calcd. for C_32_H_42_NO_5_, 520.3063), [M + Na]^+^ 542.2877 (calcd. for C_32_H_41_NNaO_5_, 542.2882).

Pyrrospirone J (**10**): Colorless amorphous powder; molecular formula C_30_H_37_NO_6_; ECD (10 mg/L, MeOH) λ_max_ (Δε) 204 (−55.93), 223 (+59.59), 256 (−11.80), 293 (−17.92) nm; UV (MeOH) λ_max_ (log ε) 211 (4.40), 230 (4.21), 284 (3.36) nm; IR (MeOH) *ν*_max_ 3347, 2923, 2851, 1748, 1686, 1505, 1460, 1371, 1239, 1169, 1074, 937 cm^−1^; ^13^C (125 MHz) and ^1^H (500 MHz) NMR data, see Table 2; HRESIMS *m*/*z* [M + H]^+^ 508.2711 (calcd. for C_30_H_38_NO_6_ 508.2699) and [M + Na]^+^ 530.2531 (calcd. for C_30_H_37_NNaO_6_ 530.2519).

Crystal data of penicipyrroether A (**9**): Colorless crystals of penicipyrroether A (**9**) was obtained from MeOH. X-ray diffraction analysis was performed on an Xcalibur Atlas Gemini Ultra diffractometer (Agilent Technologies, CA, USA) with Cu Kα radiation (λ = 1.54184 Å) at 100 K. Structure was solved by direct method (SHELXL-2018) and refined with full-matrix least-squares on *F*^2^ (ShelXL, Sheldrick, 2015). All non-hydrogen atoms were refined anisotropically and all hydrogen atoms were placed in idealized positions and refined as riding atoms with the relative isotropic parameters [2]. Crystal data of penicipyrroether A (**9**): C_32_H_41_NO_5_ (M = 519.68), orthorhombic crystal (0.13 × 0.12 × 0.09 mm), space group P212121 (no. 19), unit cell dimensions *a* = 6.32760(10) Å, *b* = 19.8687(2) Å, *c* = 25.0617(3) Å, *V* = 3150.79(7) Å^3^, *α* = 90°, *β* = 90°, *γ* = 90°; *Z* = 4; *D_calced_* = 1.231 g/cm^3^; *μ* = 0.684 mm^−1^; 18406 reflection measured (5.676° ≤ 2θ ≤ 147.088°); 6237 unique (*R*_int_ = 0.0244, *R*_sigma_ = 0.0209) which were used in all calculation; the final refinement ( *I* ≥ 2*σ* (*I*)) gave *R_1_* = 0.0418 and *wR_2_* = 0.1116 (all data); Flack parameter = 0.02 (5). Crystallographic data of penicipyrroether A (**9**) have been deposited at the Cambridge Crystallographic Data Centre (deposition number: CCDC 1868814). Copies of the crystallographic data can be obtained free of charge via www.ccdc.cam.ac.uk/conts/retrieving.html or from the Cambridge Crystallographic Data Centre, 12, Union Road, Cambridge CB2 1EZ, U.K. [fax (+44)1223-336-033; or e-mail: data_request@ccdc.cam.ac.uk).

### 3.5. ECD Calculation

Monte Carlo conformational searches of compound **10** were conducted with the Spartan’ 10 software (v1.1.0, x 86, Wavefunction Inc., Irvine, CA, USA) using Merck Molecular Force Field (MMFF) and three conformers were obtained for ECD calculations. The X-ray CIF profile of **9** and the three conformer of **10** (Tables S3 and S4 and Figures S44 and S45) were initially optimized at B3LYP/6-31g (d,p) level in MeOH using the conductor-like polarizable continuum model (CPCM). The theoretical ECD calculation was carried out in MeOH using Time-dependent Density functional theory (TD-DFT) at the B3LYP/6-31+g (d,p) level for all conformers of **9** and **10**. Rotatory strengths for a total of 60 excited states for **9** (or 30 for **10**) were calculated. ECD spectra were generated using the program SpecDis 1.6 (University of Würzburg, Würzburg, Germany) and GraphPad Prism 5 (University of California San Diego, USA) from dipole-length rotational strengths by applying Gaussian band shapes with sigma = 0.2 eV for **9** (or 0.3 eV for **10**).

### 3.6. ^13^C NMR Calculation

The previously described methods [19,20,21] were used for ^13^C NMR calculation. Briefly, Monte Carlo conformational searches were carried out by means of Spartan’s 10 software using MMFF. Four conformers (Table S8 and Figure S46) of compound **10** were obtained for NMR calculations. The conformers were initially optimized at B3LYP/6-31g (d,p) level in DMSO using the CPCM calculation model. Gauge-independent atomic orbital (GIAO) calculations of ^13^C NMR chemical shifts were performed by DFT at the mPW1PW91-SCRF (DMSO)/6-311+g(d,p) level with the CPCM calculation model in Gaussian 09 software (G09w, D01, Gaussian Inc., Wallingford, CT, USA). The calculated ^13^C NMR data of the lowest energy conformers for **10** were averaged based on the Boltzmann distribution theory and their relative Gibbs free energy.

### 3.7. Sulforhodamine B (SRB) Assay

The activity of the tested compounds in inhibiting the proliferation of human glioma U87-MG and U251 cells was determined by SRB assay [22]. Doxorubicin (DOX) was used as a positive control. U87MG cells were cultured in MEM (Minimum Essential Medium, Gibco, Grand Island, NY, USA) with 10% FBS (Fetal Bovine Serum, PAA Laboratories Inc., Toronto, ON, Canada), U251 in DMEM (Dulbecco’s Modified Eagle Medium, Gibco) and normal human astrocytes (HA) in AM (Astrocyte Medium, ScienCell, Cat. No. 1801). All cells were incubated at 37 °C in a 5% CO_2_ humidified incubator. The cultured cells after the third generation were used for experiment.

### 3.8. Antibacterial Activive Assay

The previously described micro broth dilution method [3,22] was used to evaluate the antibacterial activity of the tested compounds against the growth of MRSA and *E. coli*. Vancomycin (an antibiotic against MRSA) and gentamicin (an antibiotic against Gram-positive and Gram-negative bacteria) were used as positive controls. The microorganisms were cultured in MHB medium in 96-well plates at a concentration of 1 × 10^6^ CFU/mL. The MIC was determined after 12 h incubation at 37 °C with tested compounds.

## 4. Conclusions

It was reported that the marine-derived *Penicillium* sp. ZZ380 produced a series of pyrrospirones C–I with a characteristic spiro conjunction for rings D and E in BMPM medium [2]. This study reported two new alkaloids of penicipyrroether A (**9**), produced by strain ZZ380 in PDB medium and pyrrospirone J (**10**), a previously unidentified compound isolated from a previous culture of ZZ380 in BMPM medium [2]. Both compounds **9** and **10** possess unique structures with a 6/5/6/5 fused ring system of rings A/B/C/D, rather than the 6/5/6/6 ring fusion for pyrrospirones C–I. Each of the two new alkaloids is the first compound of its structural type. Penicipyrroether A (**9**) is a cyclo-condensation product of GKK1032 analogue via the addition of a five-membered ether ring and exhibited potent inhibitory activity against the proliferation of glioma U87MG and U251 cells and the growth of MRSA and *E. coli*.

## Figures and Tables

**Table 1:** ^13^C and ^1^H NMR data of penicipyrroether A (**9**, in pyridine-*d*_5_).

No.	*δ*_C_, Type	*δ*_H_ (*J* in Hz)	^1^H-^1^H COSY	HMBC
1	49.2, CH_2_	βH: 0.76, t (12.0); αH: 1.88, dd (12.2, 3.3)	H-2, αH-1; H-2, βH-1	C-27, C-29
2	28.5, CH	1.76, m	H-1, H-3, H_3_-27	
3	46.1, CH_2_	βH: 0.57, q (12.0); αH: 1.69, m	H-2, αH-3, H-4; H-2, βH-3, H-4	C-27, C-28
4	28.0, CH	1.97, m	H-3, H-5, H_3_-28	
5	62.0, CH	1.22, dd (11.3, 7.6)	H-4, H-9	C-4, C-6, C-29
6	41.3, C	–		
7	54.4, CH	2.36, d (13.2)	H-8	C-10
8	48.9, CH	3.12, m	H-7, H-9, H-13	C-7, C-9, C-13, C-14
9	87.5, CH	5.01, dd (7.6, 4.8)	H-5, H-8	C-6
10	139.9, C	–		
11	126.8, CH	5.62, s		C-7, C-30
12	48.6, C	–		
13	56.3, CH	3.78, d (5.7)	H-8	C-9, C-11, C-12, C-14, C-16, C-31
14	102.1, C	–		
15	79.4, CH	4.35, q (6.4)	H_3_-32	C-11, C-31
16	139.2, C	–		
17	172.7, C	–		
18	88.1, C	–		
19	147.3, CH	6.95, d (1.8)		C-14, C-17, C-18
20	45.7, CH_2_	βH: 3.56, d (12.2); αH: 3.51, d (12.2)	αH-20; βH-20	C-18, C-19, C-21, C-22, C-26
21	130.8, C	–		
22	132.7, CH	7.31, dd (8.1, 1.9)	H-23	C-20, C-24, C-26
23	122.4, CH	7.13, dd (8.1, 2.4)	H-22	C-21, C-25
24	159.6, C	–		
25	118.4, CH	7.22 ^a^	H-26	C-21, C-23
26	130.2, CH	7.38, dd (8.4, 1.9)	H-25	C-22, C-24
27	23.4, CH_3_	0.91, d (6.4)	H-2	C-1, C-2, C-3
28	20.2, CH_3_	1.18, d (6.3)	H-4	C-3, C-4, C-5
29	16.8, CH_3_	1.31, s		C-1, C-5, C-6, C-7
30	20.8, CH_3_	1.81, s		C-7, C-10, C-11
31	22.0, CH_3_	1.35, s		C-11, C-12, C-13, C-15
32	14.8, CH_3_	1.26, d (6.4)	H-15	C-12, C-15
OH-14	–	6.25, s		C-14, C-16
OH-18	–	8.35, s		
NH-17	–	9.43, s		C-16, C-18, C-19

^a^ The signal was overlapped with that of NMR solvent pyridine-*d*_5_.

**Table 2:** ^13^C and ^1^H NMR data of pyrrospirone J (**10**, in DMSO-*d*_6_).

No.	*δ*_C_, Type	*δ*_H_ (*J* in Hz)	^1^H-^1^H COSY	HMBC
1	47.3, CH_2_	βH: 0.81, t (12.3); αH: 1.80, m	H-2, αH-2; H-2, βH-2	C-26, C-28
2	27.4, CH	1.82, m	H-1, H-3, H_3_-26	C-6
3	45.5, CH_2_	βH: 0.51, q (12.1); αH: 1.75, m	H-2, αH, H-4 H-2, βH, H-4	C-2, C-27
4	26.8, CH	1.78, m	H-3, H-5, H_3_-27	C-2
5	59.5, CH	1.22, dd (11.6, 8.8)	H-4, H-9	C-4, C-6, C-28
6	42.5, C	–		
7	49.9, CH	1.55, d (14.3)	H-8	C-1, C-5, C-6, C-8, C-13, C-28
8	39.2, CH	2.79, m	H-7, H-9, H-13	C-7, C-9, C-10, C-13, C-14
9	85.0, CH	4.82, dd (8.6, 7.0)	H-5, H-8	C-5, C-6, C-13, C-23
10	58.8, C	–		
11	63.5, CH	2.46, s		C-10, C-12, C-13, C-29, C-30
12	81.3, C	–		
13	44.5, CH	3.21, d (8.0)		C-8, C-9, C-11, C-12, C-14, C-30
14	180.9, C	–		
15	79.7, C	–		
16	172.1, C	–		
17	86.2, C	–		
18	40.9, CH_2_	βH: 1.99, d (12.1); αH: 2.45, d (12.1)	αH-18; βH-18	C-14, C-15, C-16, C-17, C-19
19	45.0, CH_2_	βH: 2.99, d (14.5); αH: 2.69, d (14.5)	αH-19; βH-19	C-17, C-18, C-20, C-21, C-25
20	129.4, C	–		
21	133.3, CH	6.92, dd (8.3, 2.1)	H-22	C-19, C-23, C-25
22	124.4, CH	6.82, dd (8.3, 2.7)	H-21	C-20, C-23, C-24
23	158.7, C	–		
24	119.7, CH	6.94, dd (8.8, 2.7)	H-25	C-20, C-22, C-23
25	130.6, CH	6.78, dd (8.8, 2.1)	H-24	C-19, C-21, C-23
26	22.7, CH_3_	0.86, d (6.1)	H-2	C-1, C-2, C-3
27	19.7, CH_3_	1.02, d (6.2)	H-4	C-3, C-4, C-5
28	15.2, CH_3_	1.05, s		C-1, C-5, C-6, C-7
29	21.0, CH_3_	1.20, s		C-7, C-10, C-11
30	26.0, CH_3_	1.59, s		C-11, C-12, C-13
OH-17	–	6.24, s		C-17, C-18, C-19
NH-16	–	8.76, s		C-15, C-16, C-17, C-18

**Table 3:** Antiglioma and antibacterial activities of penicipyrroether A (**9**) and pyrrospirone J (**10**).

Compounds	Glioma Cells (μM)		Bacteria (μg/mL)	
	U87MG	U251	MRSA	*E. coli*
**9**	1.64 ± 0.05	5.50 ± 0.12	1.7	3.0
**10**	10.52 ± 0.62	17.92 ± 0.93	>50	>50
DOX	1.20 ± 0.06	8.03 ± 1.20	NT	NT
Gentamicin	NT	NT	0.36	1.44
Vancomycin	NT	NT	0.20	NT

NT: No testing.

## Supplementary Materials

The following is available online at https://www.mdpi.com/1660-3397/17/5/292/s1, Figures S1–S22: NMR, HRESIMS, UV and IR spectra of penicipyrroether A (**9**), Figures S23–S43: NMR, HRESIMS, UV and IR spectra of pyrrospirone J (**10**), Figure S44: The optimized geometry of conformer (**9-1**) of penicipyrroether A (**9**), Figure S45: The optimized geometry of conformers (**10-1**–**10-3**) of pyrrospirone J (**10**), Figure S46: Four conformations of the low-energy conformers of pyrrospirone J (**10**) calculated at B3LYP/6-31G(d) level, Table S1: ^13^C NMR data of known compounds **11**–**15**, Table S2: ^13^C NMR data of known compounds **16**–**18**, Table S3: Gibbs free energies and equilibrium populations of low-energy conformer of penicipyrroether A (**9**), Table S4: Cartesian coordinates for the low-energy reoptimized MMFF conformers of penicipyrroether A (**9**) at B3LYP/6-311+G(d,p) level of theory in CH_3_OH, Table S5: Gibbs free energies and equilibrium populations of low-energy conformers of pyrrospirone J (**10**), Table S6: Cartesian coordinates for the low-energy reoptimized MMFF conformers of pyrrospirone J (**10**) at B3LYP/6-311+G(d,p) level of theory in CH_3_OH, Table S7: Experimental and calculated ^13^C NMR data of pyrrospirone J (**10**), Table S8: Gibbs free energies and equilibrium populations of the low-energy conformers of pyrrospirone J (**10**) for ^13^C NMR calculation, Table S9: Cartesian coordinates for the low-energy reoptimized MMFF conformers of pyrrospirone J (**10**) at B3LYP/6-311+G(d,p) level of theory in DMSO for ^13^C NMR calculation.
