# Supplementary material for: Novel Bioactive Penicipyrroether A and Pyrrospirone J from the Marine-Derived Penicillium sp. ZZ380
Source: Mar Drugs. 2019 May 15;17(5):292. doi: 10.3390/md17050292 (PMC6562518; doi:10.3390/md17050292)
Supplement: Supplementary file 1 [file marinedrugs-17-00292-s001.pdf]

marine drugs

Supplementary Materials for

Novel Bioactive Penicipyrroether A and Pyrrospirone J from the Marine-derived *Penicillium* sp. ZZ380

Tengfei Song [1], Mingmin Tang [1], Hengju Ge [2], Mengxuan Chen [2], Xiaoyuan Lian [1,]* and Zhizhen Zhang [2,]*

[1] College of Pharmaceutical Sciences, Zhejiang University, Hangzhou 310058, China; tmm0907@163.com (M.T.)

[2] Ocean College, Zhoushan Campus, Zhejiang University, Zhoushan 316021, China; sdasheng72@163.com (T.S.), 15805152141@163.com (H.G.), zerocmx@163.com

* Correspondence: xylian@zju.edu.cn (X.L.); zzhang88@zju.edu.cn (Z.Z.);
Tel.: +86-135-7547-6388 (X.L.); +86-136-7585-9706 (Z.Z.)

CONTENT

Table S1. ^{13}C NMR data of known compounds **11–15** ············4
Table S2. ^{13}C NMR data of known compounds **16–18**············4
Figures S1–4. ^{1}H NMR spectra of penicipyrroether A (**9**)············6
Figures S5–7. ^{13}C NMR spectra of penicipyrroether A (**9**)············8
Figures S8–10. ^{1}H-^{1}H COSY spectra of penicipyrroether A (**9**) ············9
Figures S11–13. HSQC spectra of penicipyrroether A (**9**)············11
Figures S14–17. HMBC spectra of penicipyrroether A (**9**)············12
Figures S18–19. NOESY spectra of penicipyrroether A (**9**)············14
Figure S20. HRESIMS spectrum of penicipyrroether A (**9**)············15
Figure S21. UV spectrum of penicipyrroether A (**9**) ············16
Figure S22. IR spectrum of penicipyrroether A (**9**) ············16
Figures S23–26. ^{1}H NMR spectra of pyrrospirone J (**10**)············18
Figures S27–30. ^{13}C NMR spectra of pyrrospirone J (**10**)············20
Figures S31–32. ^{1}H-^{1}H COSY spectra of pyrrospirone J (**10**) ············22
Figures S33–34. HSQC spectra of pyrrospirone J (**10**)············23
Figures S35–38. HMBC spectra of pyrrospirone J (**10**)············24
Figures S39–40. NOESY spectra of pyrrospirone J (**10**)············26
Figure S41. HRESIMS spectrum of pyrrospirone J (**10**)············27
Figure S42. UV spectrum of pyrrospirone J (**10**) ············27
Figure S43. IR spectrum of pyrrospirone J (**10**) ············28
Figure S44. The optimized geometry of conformer (**9-1**) of penicipyrroether A (**9**) ············28
Table S3. Gibbs free energies and equilibrium populations of low-energy conformer of penicipyrroether A (**9**) ············28
Table S4. Cartesian coordinates for the low-energy reoptimized MMFF conformers of penicipyrroether A (**9**) at B3LYP/6-311+G(d,p) level of theory in CH_3OH············29
Figure S45. The optimized geometry of conformers (**10-1–10-3**) of pyrrospirone J (**10**) ·····31
Table S5. Gibbs free energies and equilibrium populations of low-energy conformers of pyrrospirone J (**10**) ············31
Table S6. Cartesian coordinates for the low-energy reoptimized MMFF conformers of pyrrospirone J (**10**) at B3LYP/6-311+G(d,p) level of theory in CH_3OH············31
Table S7. Experimental and calculated ^{13}C NMR data of pyrrospirone J (**10**) ············37
Figure S46. Four conformations of the low-energy conformers of pyrrospirone J (**10**) calculated at B3LYP/6-31G(d) level············38
Table S8. Gibbs free energies and equilibrium populations of the low-energy conformers of pyrrospirone J (**10**) for ^{13}C NMR calculation············38
Table S9. Cartesian coordinates for the low-energy reoptimized MMFF conformers of pyrrospirone J (**10**) at B3LYP/6-311+G(d,p) level of theory in DMSO for ^{13}C NMR calculation············38

Structures of known compounds **11–18**

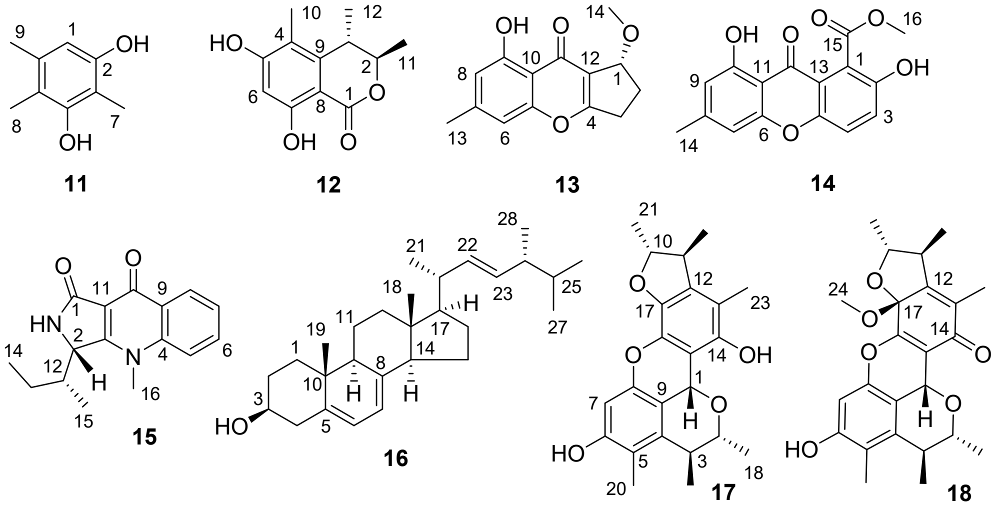

2,4,5-Trimethylresorcinol (**11**): Colorless powder; ^{13}C NMR (150 MHz, in DMSO-d_6) NMR data, see Table S_1.

Stoloniferol B (**12**): Colorless powder; $[\alpha]_D^{17}$ +109.9°, (*c* 0.5, $CHCl_3$); ^{13}C NMR (150 MHz, in DMSO-d_6) NMR data, see Table S_1.

Coniochaetones E (**13**): Colorless powder; $[\alpha]_D^{25}$ +47.3° (*c* 0.1, MeOH); ^{13}C NMR (125 MHz, in DMSO-d_6) NMR data, see Table S_1.

Pinselin (**14**): Yellow powder; ^{13}C NMR (125 MHz, in DMSO-d_6) NMR data, see Table S_1.

Quinolactacin A_1 (**15**): Colorless powder; $[\alpha]_D^{25}$ +33.7° (*c* 0.1, $CHCl_3$); ^{13}C NMR (150 MHz, in DMSO-d_6) NMR data, see Table S_1.

Ergosterol (**16**): Colorless powder; $[\alpha]_D^{20}$ –163.5° (*c* 0.3, $CHCl_2$); ^{13}C NMR (125 MHz, in $CDCl_3$) NMR data, see Table S_2.

Penicitrinol A (1**7**): Colorless powder; $[\alpha]_D^{20}$ +12.7° (*c* 0.4, MeOH); ^{13}C NMR (150 MHz, in DMSO-d_6) NMR data, see Table S_2.

Penicitrinol B (1**8**): Yellow powder; $[\alpha]_D^{25}$+18.4° (*c* 0.2, MeOH); ^{13}C NMR (150 MHz, in DMSO-d_6) NMR data, see Table S_2.

Table S1. ^{13}C NMR (150 MHz or 125* MHz, in DMSO-d_6) data of known compounds **11–15**

No.	**11**	**12**	**13***	**14***	**15**
	δ_C, type	δ_C, type	δ_C, type	δ_C, type	δ_C, type
1	108.2^{a}, CH	168.2, C	78.8, CH	117.4^{a}, C	168.9, C
2	153.4^{b}, C	79.5, CH	26.9, CH_2	151.5^{b}, C	57.1, CH
3	108.3^{a}, C	33.7, CH	29.6, CH_2	125.8, CH	164.7, C
4	153.0^{b}, C	114.0, C	174.5, C	120.5, CH	141.4, C
5	113.3, C	163.6^{a}, C	156.7, C	149.1^{c}, C	117.3, CH
6	133.2, C	100.2^{b}, CH	107.8^{a}, CH	155.9^{b}, C	132.8, CH
7	9.1^{c}, CH_3	161.5^{a}, C	146.9, C	107.9^{d}, CH	124.5^{a}, CH
8	11.8^{c}, CH_3	97.9^{b}, C	112.2, CH	149.7^{c}, C	126.0^{a}, CH
9	19.9, CH_3	142.8, C	160.1, C	111.2, CH	128.1, C
10		9.8, CH_3	108.1^{a}, C	161.0, C	171.9, C
11		19.5^{c}, CH_3	180.4, C	106.4^{d}, C	110.5, C
12		19.4^{c}, CH_3	119.3, C	180.7, C	36.3, CH
13			21.7, CH_3	117.7^{a}, C	27.5, CH_2
14			56.3, CH_3	22.5, CH_3	12.1^{b}, CH_3
15				167.3, C	11.8^{b}, CH_3
16				52.7, CH_3	35.9, CH_3

$^{a\text{-}d}$ Data with the same labels in each column may be interchanged.

Table S2. ^{13}C NMR (150 MHz, in DMSO-d_6, or 125* MHz, in $CDCl_3$) data of known compounds **16–18**

**16***				**17**				**18**			
No.	δ_C, type	No.	δ_C, type	No.	δ_C, type	No.	δ_C, type	No.	δ_C, type	No.	δ_C, type
1	38.5^{a}, CH_2	15	23.1^{e}, CH_2	1	65.3, CH	15	106.0^{d}, C	1	60.7, CH	15	110.7^{c}, C
2	32.0^{b}, CH_2	16	28.4^{b}, CH_2	2	78.3, CH	16	132.0^{e}, C	2	78.8, CH	16	156.4^{a}, C
3	70.4, CH	17	55.8^{g}, CH	3	36.9, CH	17	137.5^{a}, C	3	37.4, CH	17	97.7^{b}, C
4	40.8^{a}, CH_2	18	12.1, CH_3	4	138.4^{a}, C	18	21.8^{f}, CH_3	4	140.3, C	18	22.7^{d}, CH_3
5	139.9^{c}, C	19	16.4^{h}, CH_3	5	117.3^{b}, C	19	20.7^{f}, CH_3	5	119.5, C	19	21.0^{d}, CH_3
6	119.6^{d}, CH	20	40.6^{a}, CH	6	156.0, C	20	10.9^{g}, CH_3	6	156.0^{a}, C	20	10.7^{e}, CH_3
7	116.4^{d}, CH	21	21.2^{e}, CH_3	7	99.2, CH	21	19.9^{f}, CH_3	7	99.2^{b}, CH	21	20.2^{d}, CH_3
8	141.4^{c}, C	22	135.7^{i}, CH	8	145.7^{c}, C	22	19.1^{f}, CH_3	8	144.4, C	22	16.9, CH_3
9	46.3, CH	23	132.0^{i}, CH	9	108.8^{d}, C	23	11.4^{g}, CH_3	9	111.2^{c}, C	23	10.9^{e}, CH_3
10	37.1^{a}, C	24	42.9^{f}, CH	10	86.5, CH			10	82.8, CH	24	50.6, CH_3
11	21.2^{e}, CH_2	25	33.2^{b}, CH	11	43.4, CH			11	41.0, CH		
12	39.1^{a}, CH_2	26	20.1^{e}, CH_3	12	132.4^{e}, C			12	151.6, C		
13	42.9^{f}, C	27	19.7^{e}, CH_3	13	114.4^{b}, C			13	128.1, C		
14	54.6^{g}, CH	28	17.7^{h}, CH_3	14	147.4^{c}, C			14	185.6, C		

$^{a\text{-}i}$ Data with the same labels in each column may be interchanged.

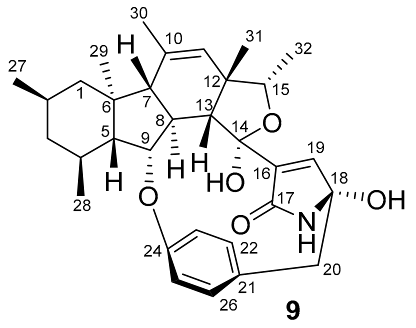

9

^{13}C and ^{1}H NMR data of penicipyrroether A (**9**, in pyridine-d_5)

No.	δ_C, type	δ_H (J in Hz)	No.	δ_C, type	δ_H (J in Hz)
1	49.2, CH_2	βH: 0.76, t (12.0); αH: 1.88, dd (12.2, 3.3)	19	147.3, CH	6.95, d (1.8)
2	28.5, CH	1.76, m	20	45.7, CH_2	βH: 3.56, d (12.2), αH: 3.51, d (12.2)
3	46.1, CH_2	βH: 0.57, q (12.0); αH: 1.69, m	21	130.8, C	–
4	28.0, CH	1.97, m	22	132.7, CH	7.31, dd (8.1, 1.9)
5	62.0, CH	1.22, dd (11.3, 7.6)	23	122.4, CH	7.13, dd (8.1, 2.4)
6	41.3, C	–	24	159.6, C	–
7	54.4, CH	2.36, d (13.2)	25	118.4, CH	7.22[a]
8	48.9, CH	3.12, m	26	130.2, CH	7.38, dd (8.4, 1.9)
9	87.5, CH	5.01, dd (7.6, 4.8)	27	23.4, CH_3	0.91, d (6.4)
10	139.9, C	–	28	20.2, CH_3	1.18, d (6.3)
11	126.8, CH	5.62, s	29	16.8, CH_3	1.31, s
12	48.6, C	–	30	20.8, CH_3	1.81, s
13	56.3, CH	3.78, d (5.7)	31	22.0, CH_3	1.35, s
14	102.1, C	–	32	14.8, CH_3	1.26, d (6.4)
15	79.4, CH	4.35, q (6.4)	OH-14	–	6.25, s
16	139.2, C	–	OH-18	–	8.35, s
17	172.7, C	–	NH-17	–	9.43, s
18	88.1, C	–			

[a] The signal was overlapped with that of NMR solvent pyridine-d_5.

Figure S1. ^{1}H NMR spectrum of penicipyrroether A (**9**)

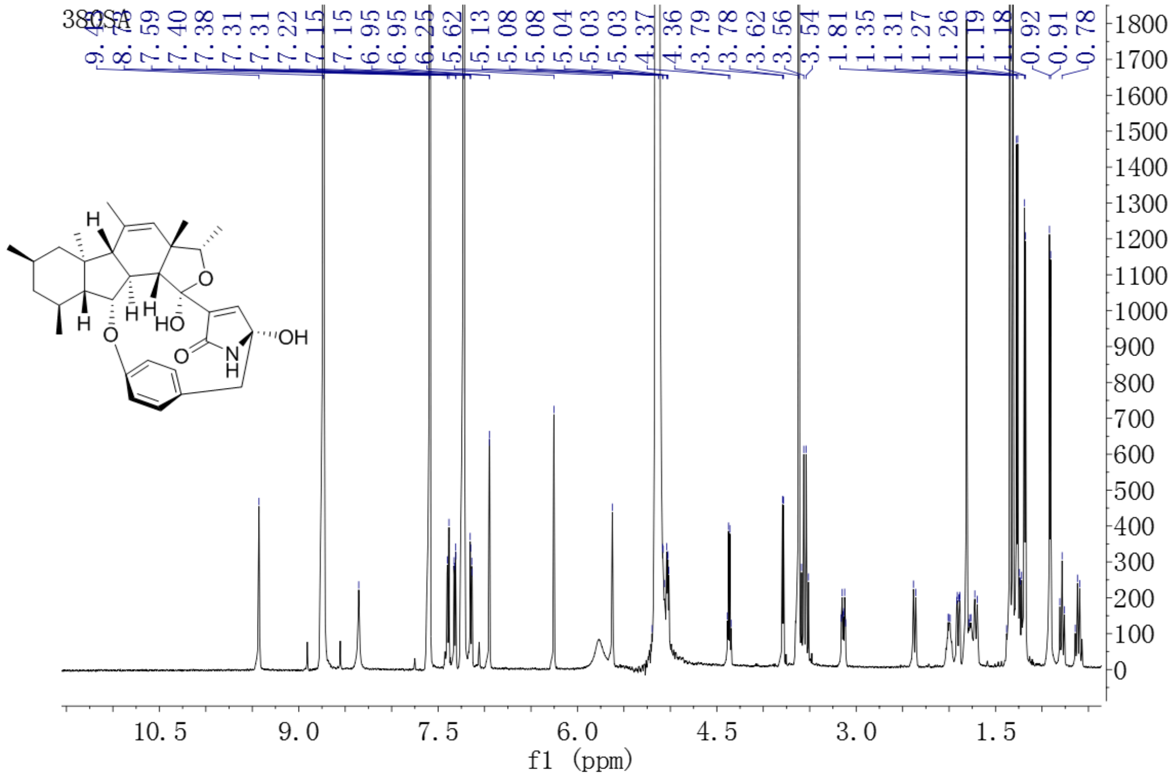

Figure S2. ^{1}H NMR spectrum of penicipyrroether A (**9**)

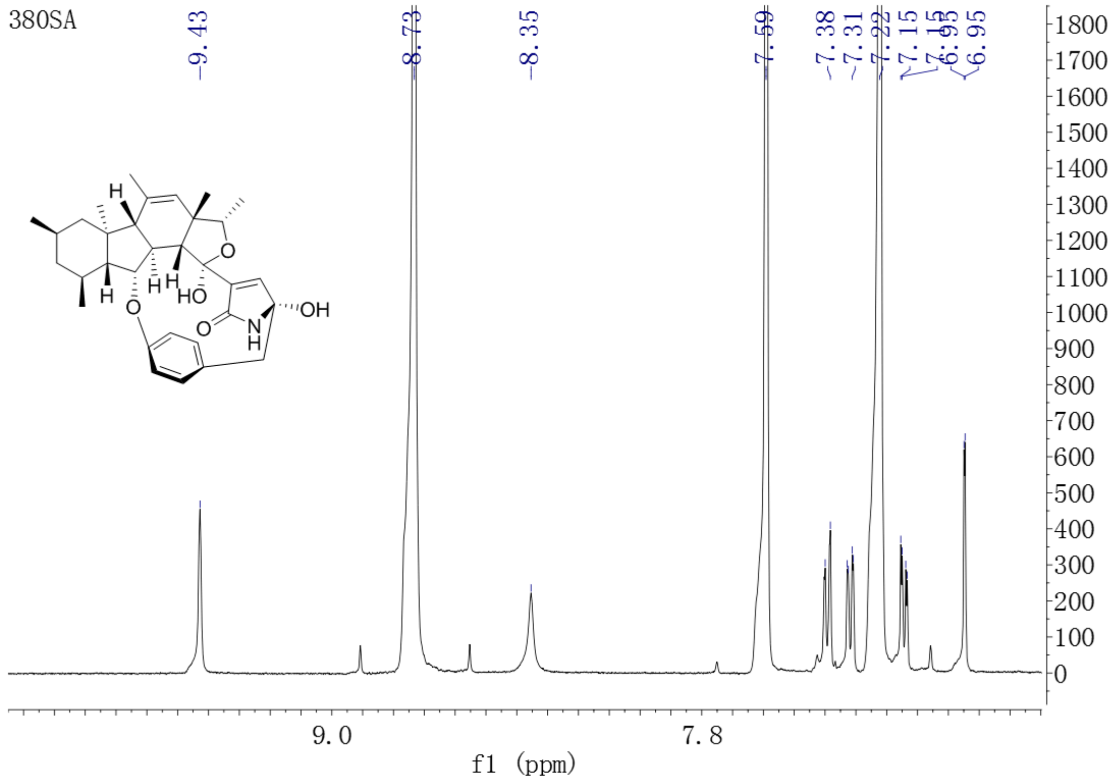

Figure S3. ^{1}H NMR spectrum of penicipyrroether A (**9**)

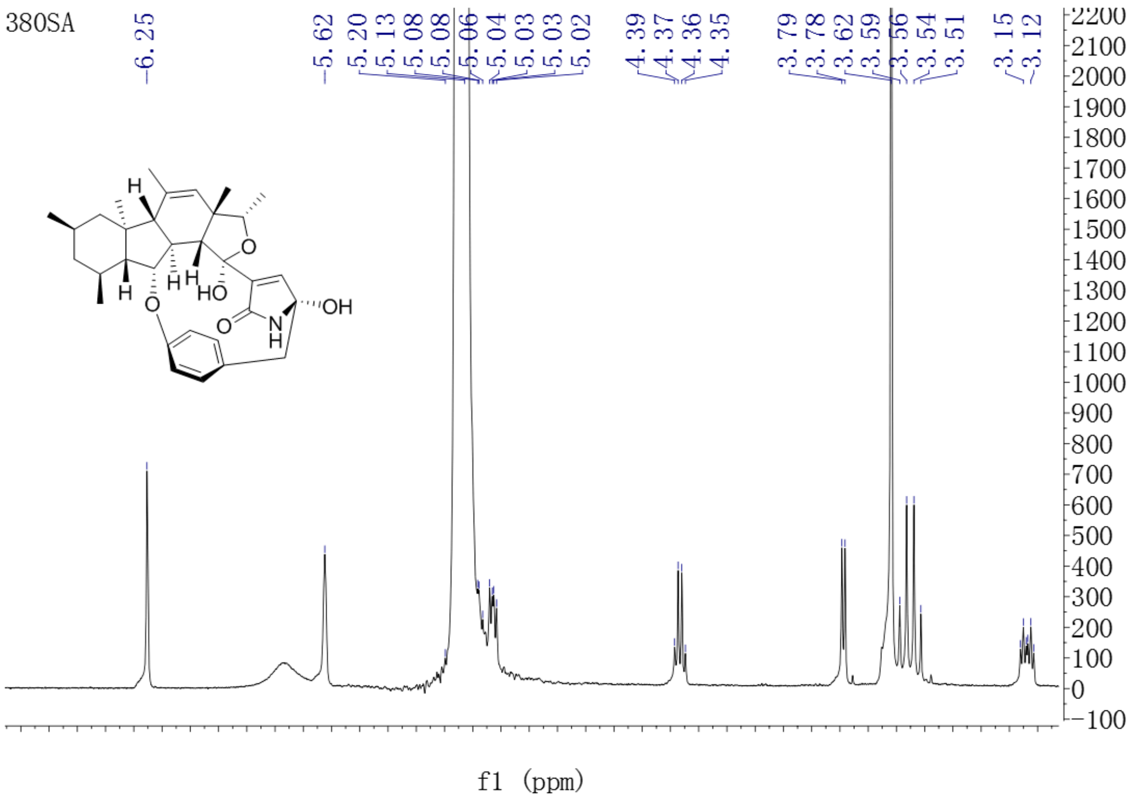

Figure S4. ^{1}H NMR spectrum of penicipyrroether A (**9**)

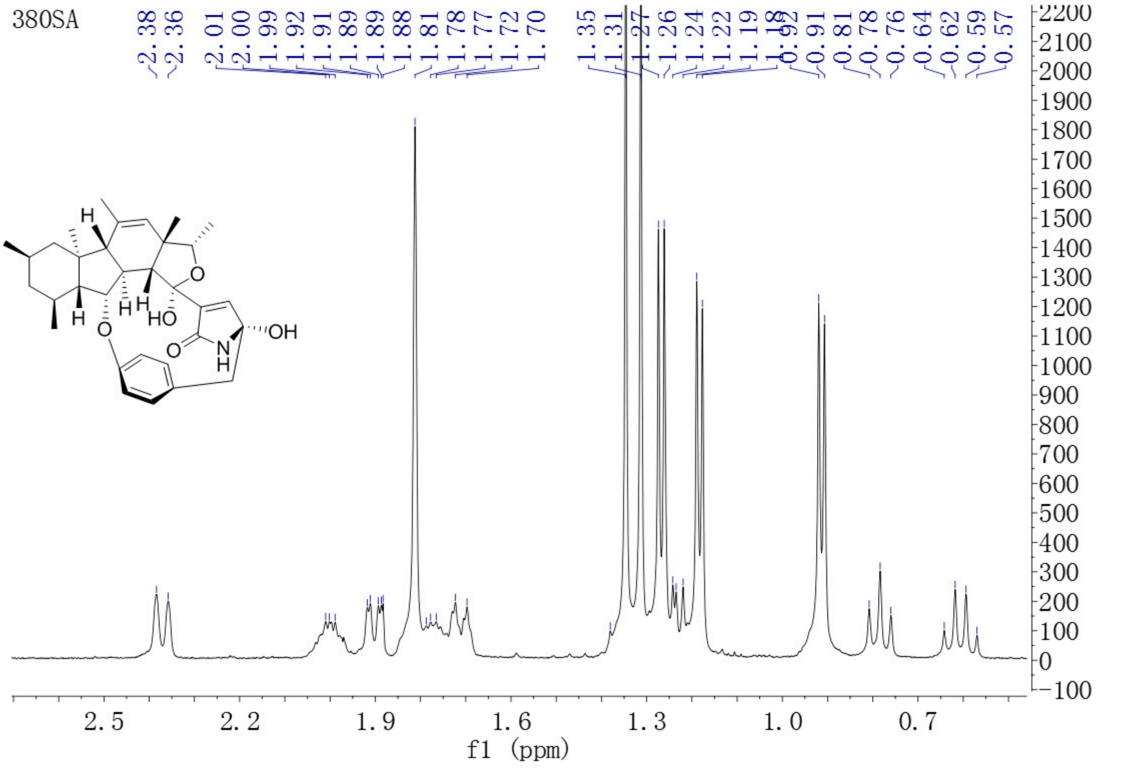

Figure S5. ^{13}C NMR spectrum of penicipyrroether A (**9**)

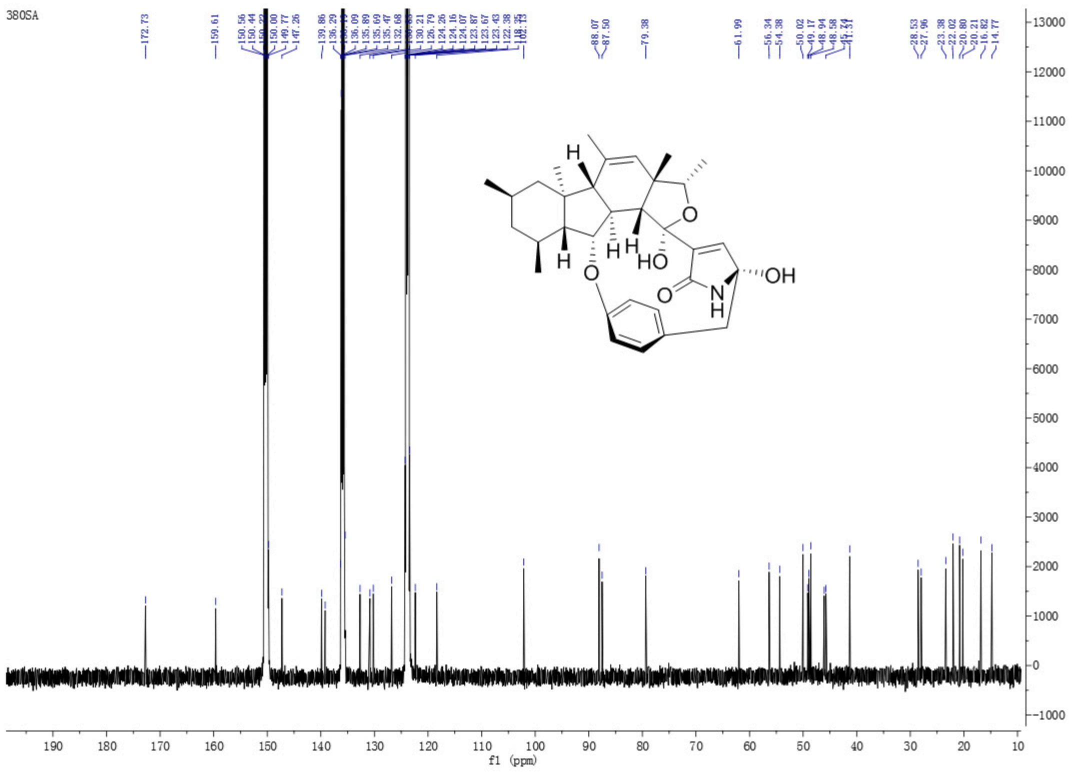

Figure S6. ^{13}C NMR spectrum of penicipyrroether A (**9**)

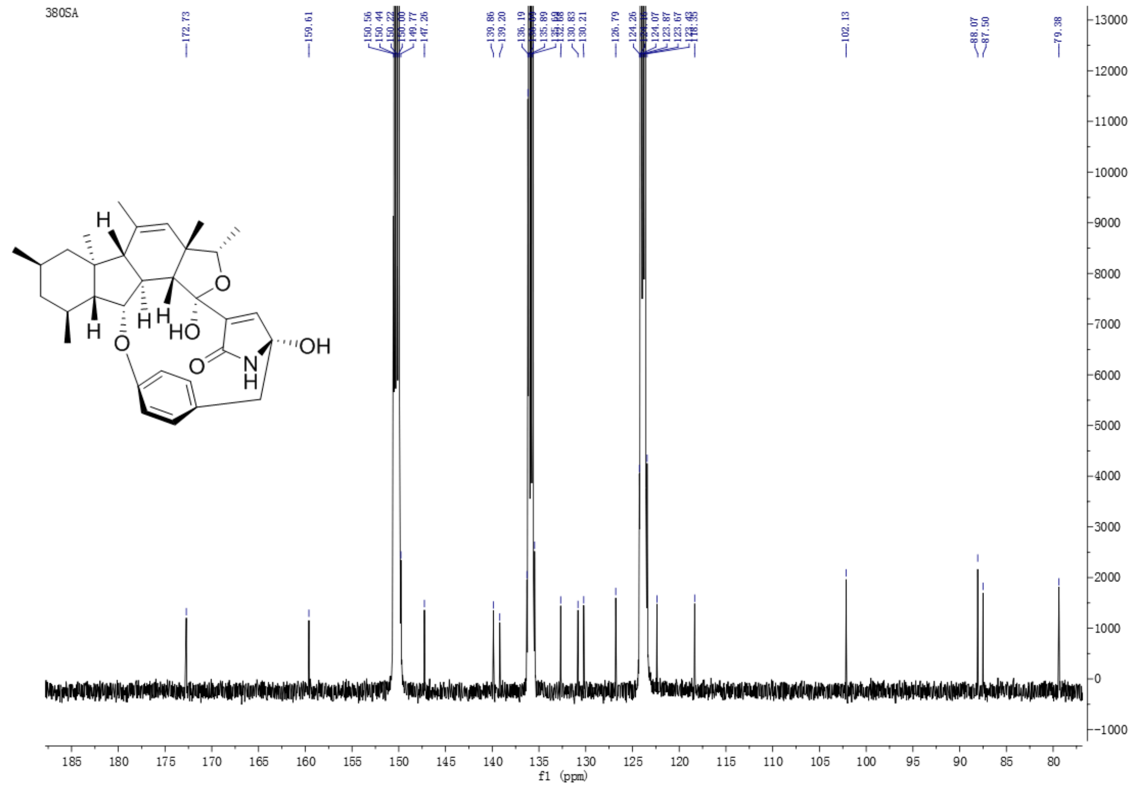

Figure S7. ^{13}C NMR spectrum of penicipyrroether A (**9**)

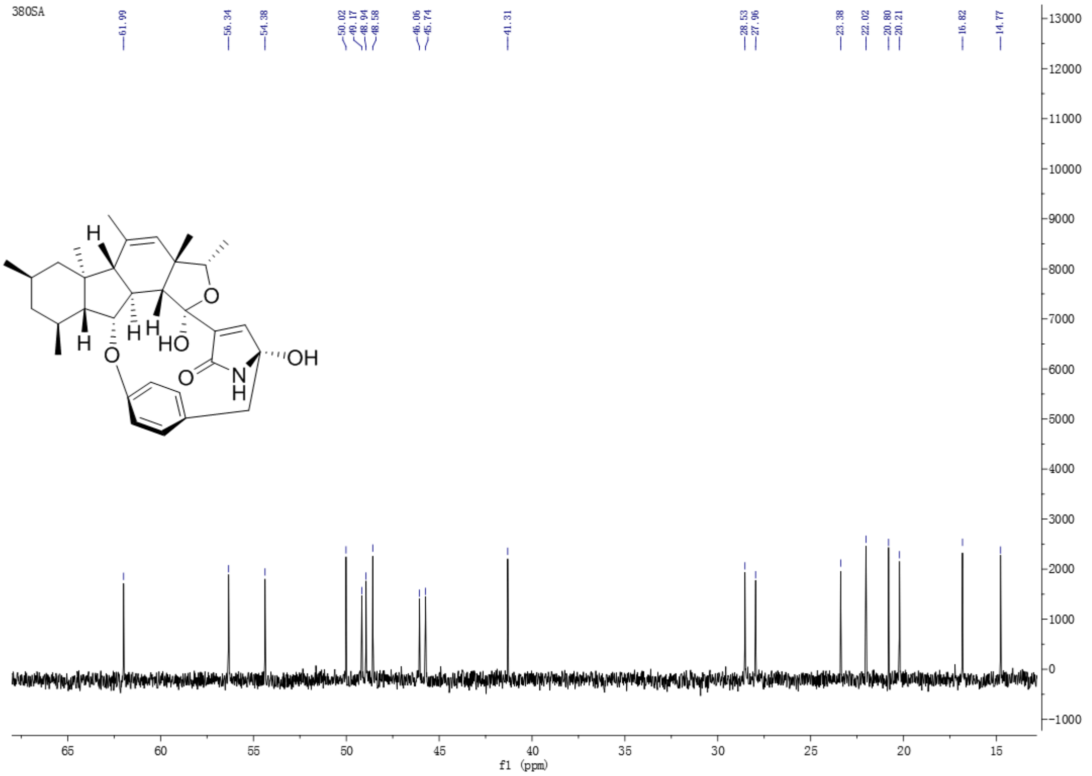

Figure S8. ^{1}H-^{1}H COSY spectrum of penicipyrroether A (**9**)

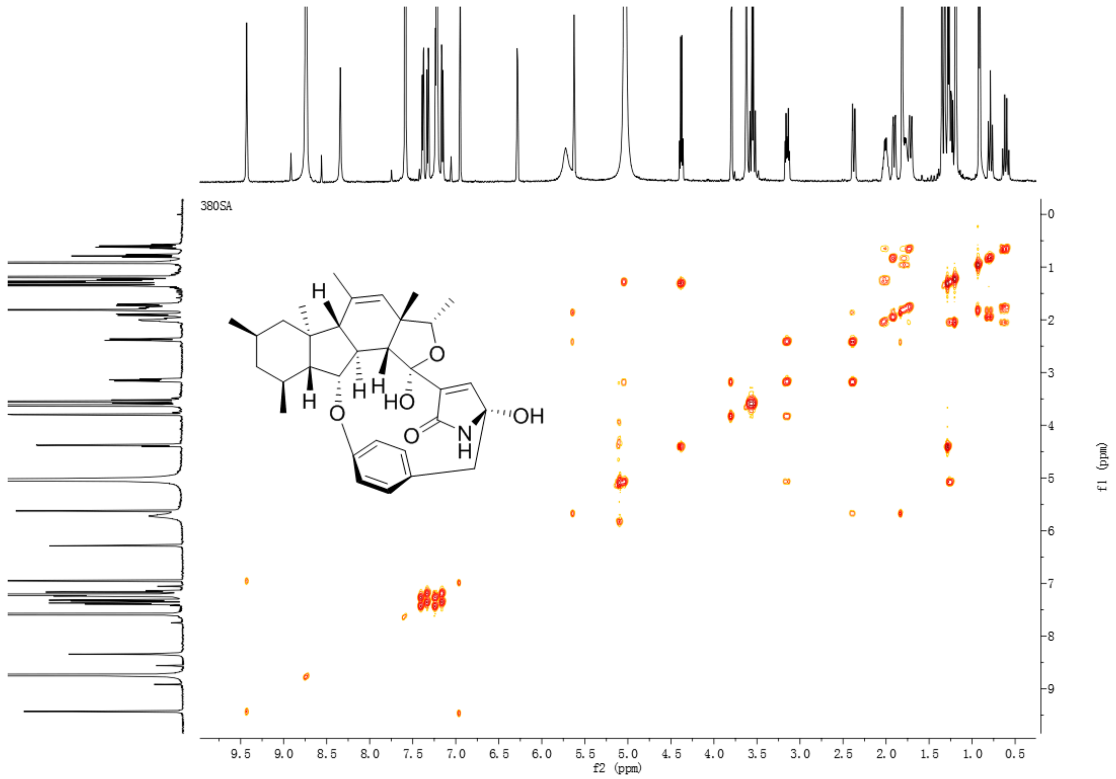

Figure S9. ^{1}H-^{1}H COSY spectrum of penicipyrroether A (**9**)

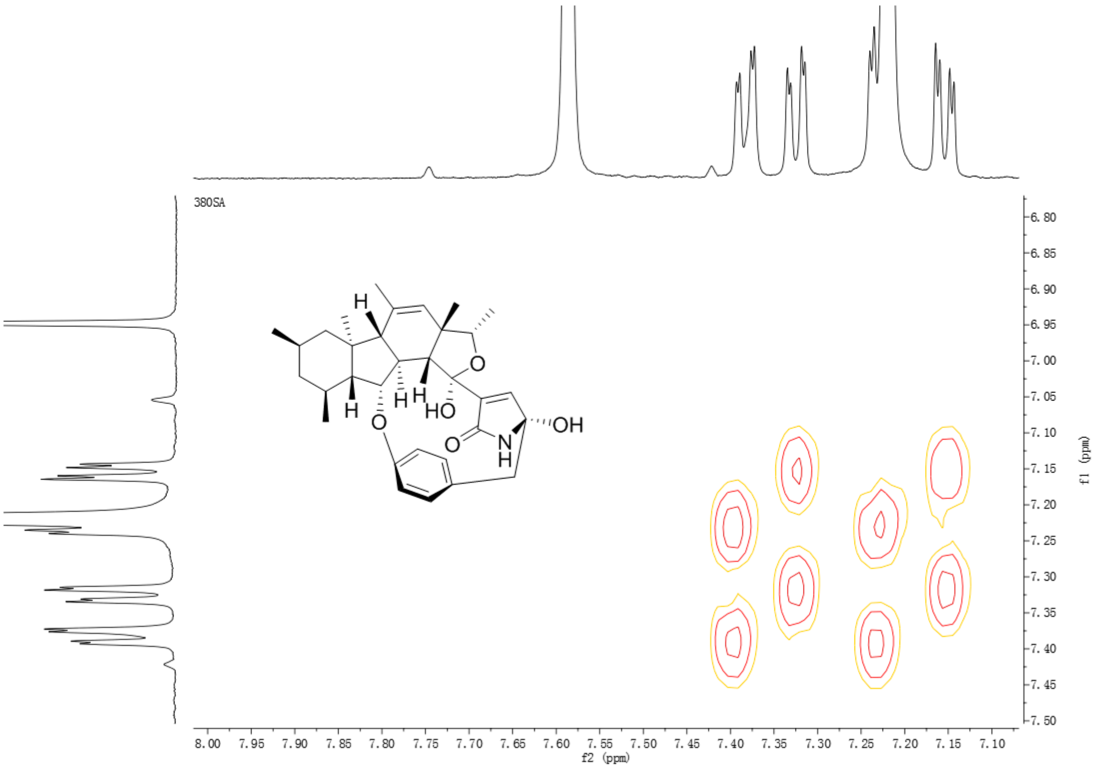

Figure S10. ^{1}H-^{1}H COSY spectrum of penicipyrroether A (**9**)

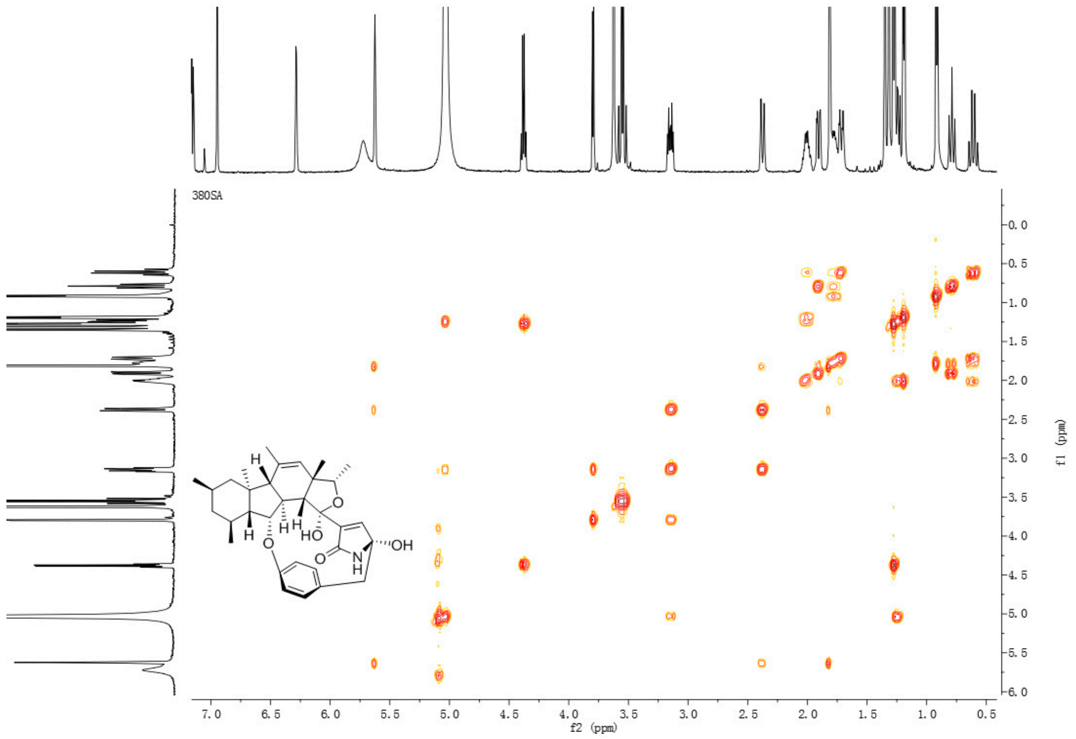

Figure S11. HSQC spectrum of penicipyrroether A (**9**)

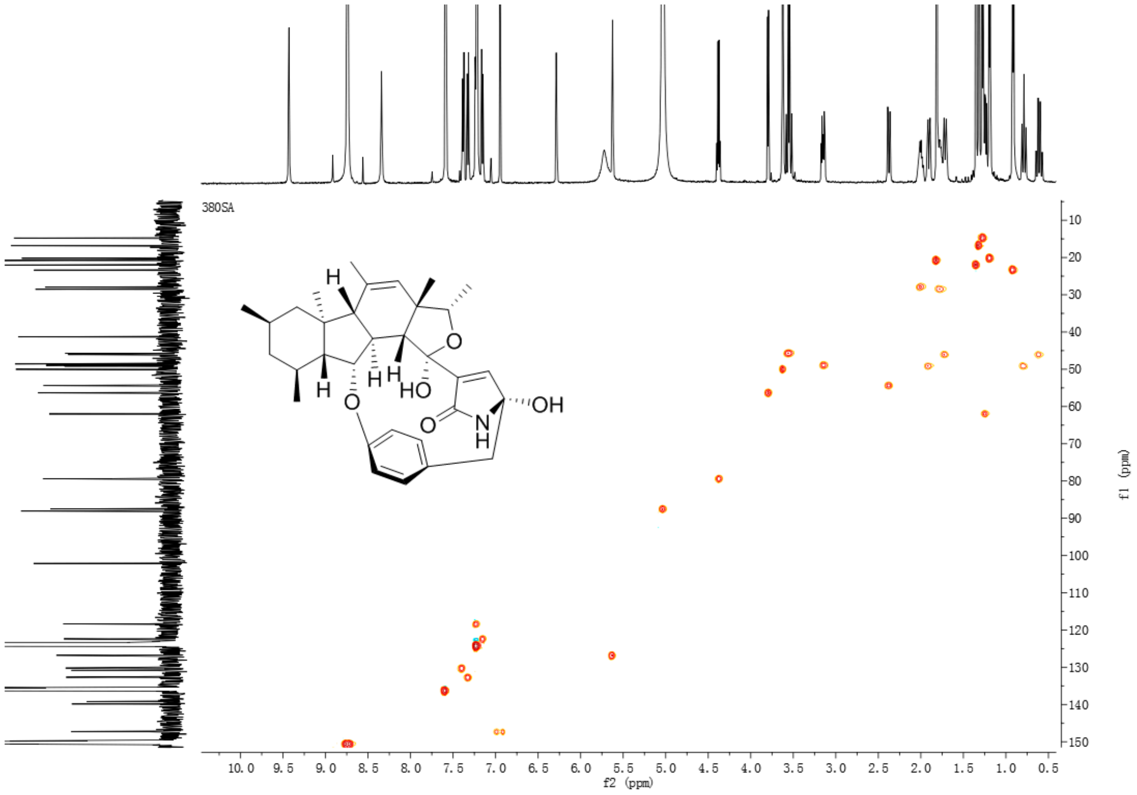

Figure S12. HSQC spectrum of penicipyrroether A (**9**)

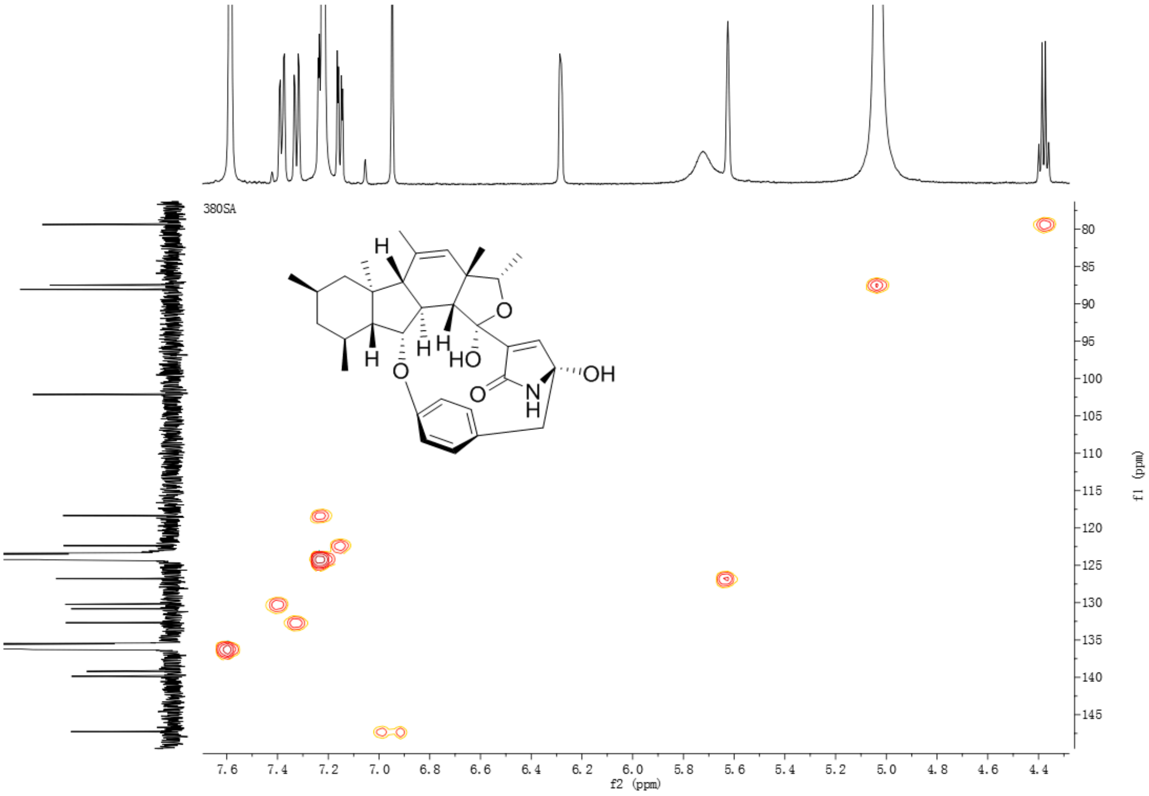

Figure S13. HSQC spectrum of penicipyrroether A (**9**)

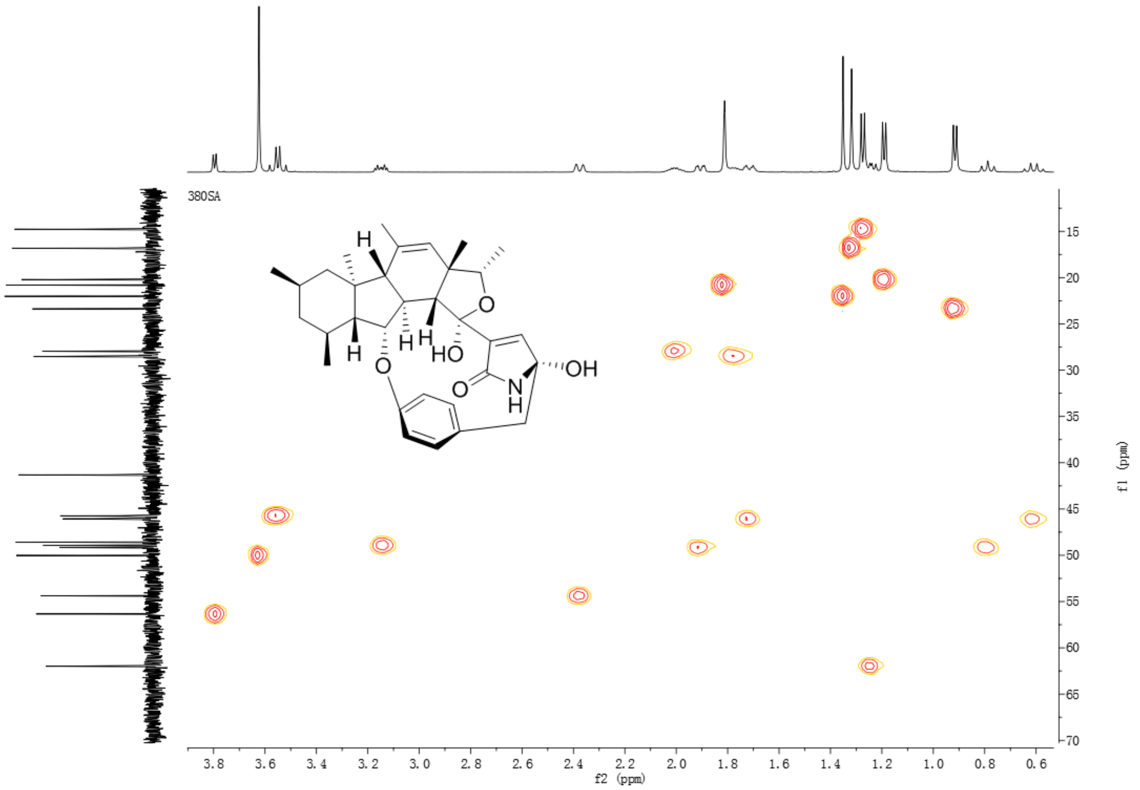

Figure S14. HMBC spectrum of penicipyrroether A (**9**)

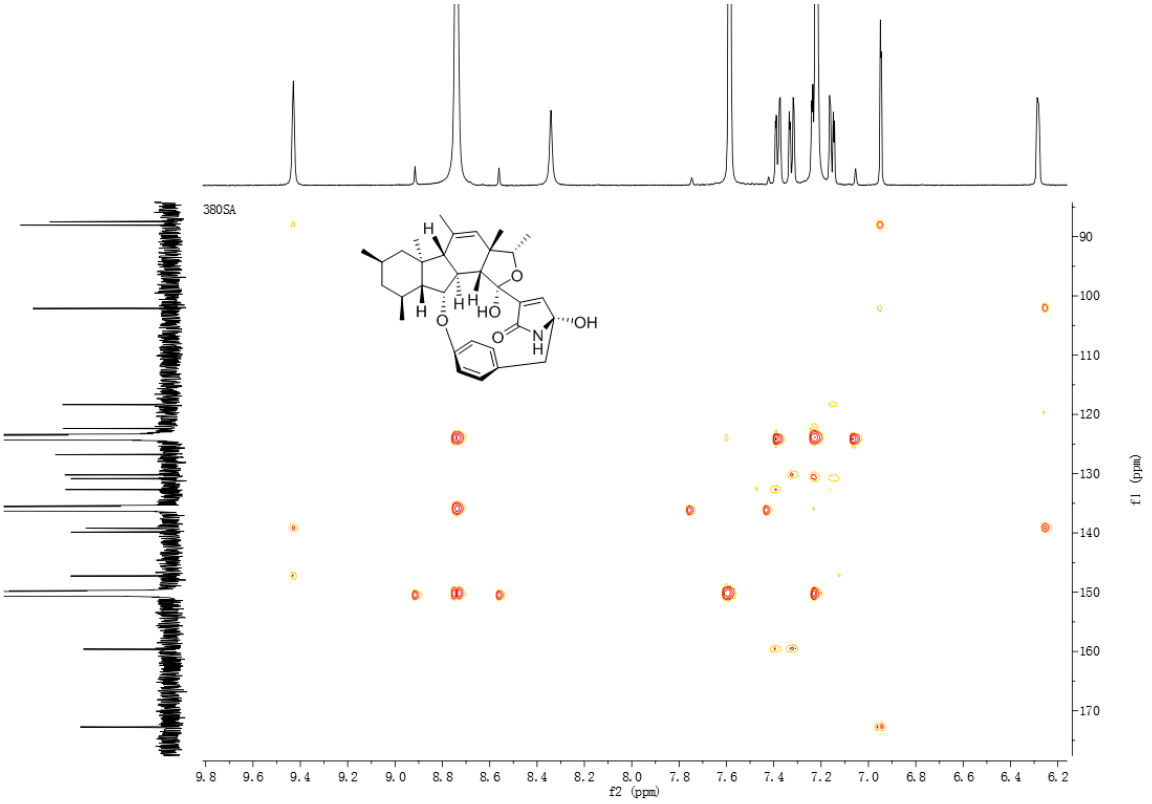

Figure S15. HMBC spectrum of penicipyrroether A (**9**)

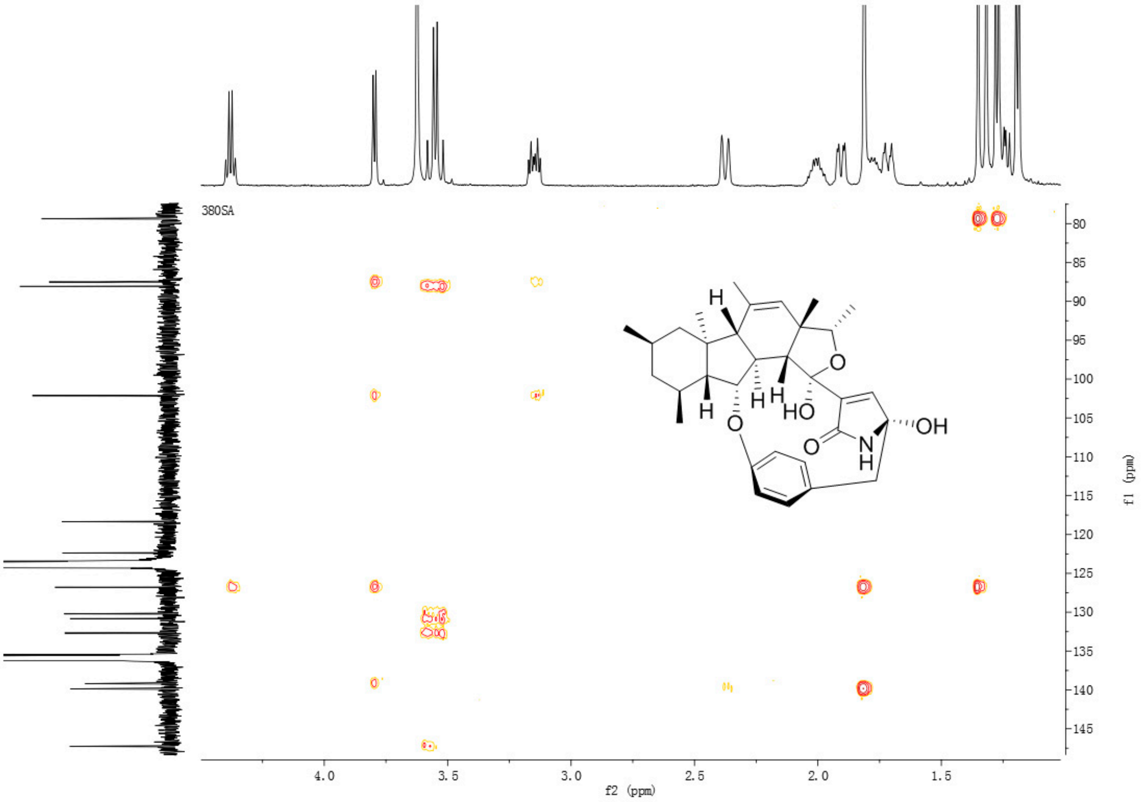

Figure S16. HMBC spectrum of penicipyrroether A (**9**)

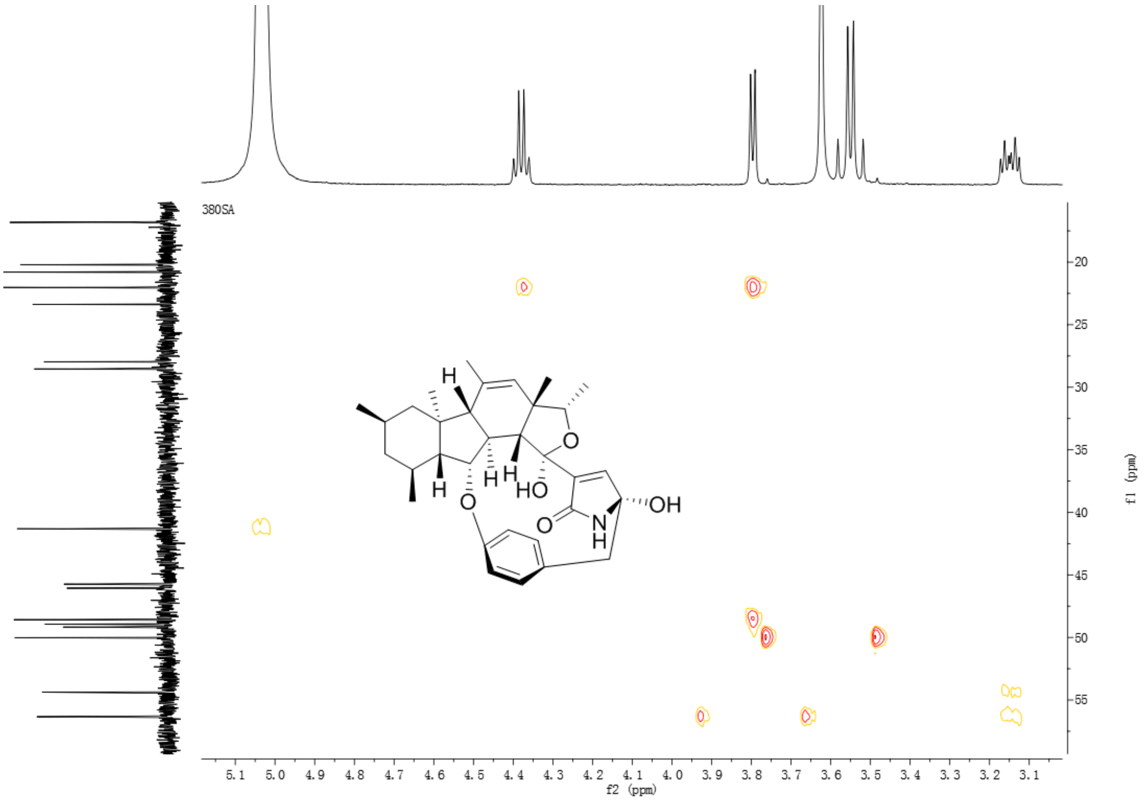

Figure S17. HMBC spectrum of penicipyrroether A (**9**)

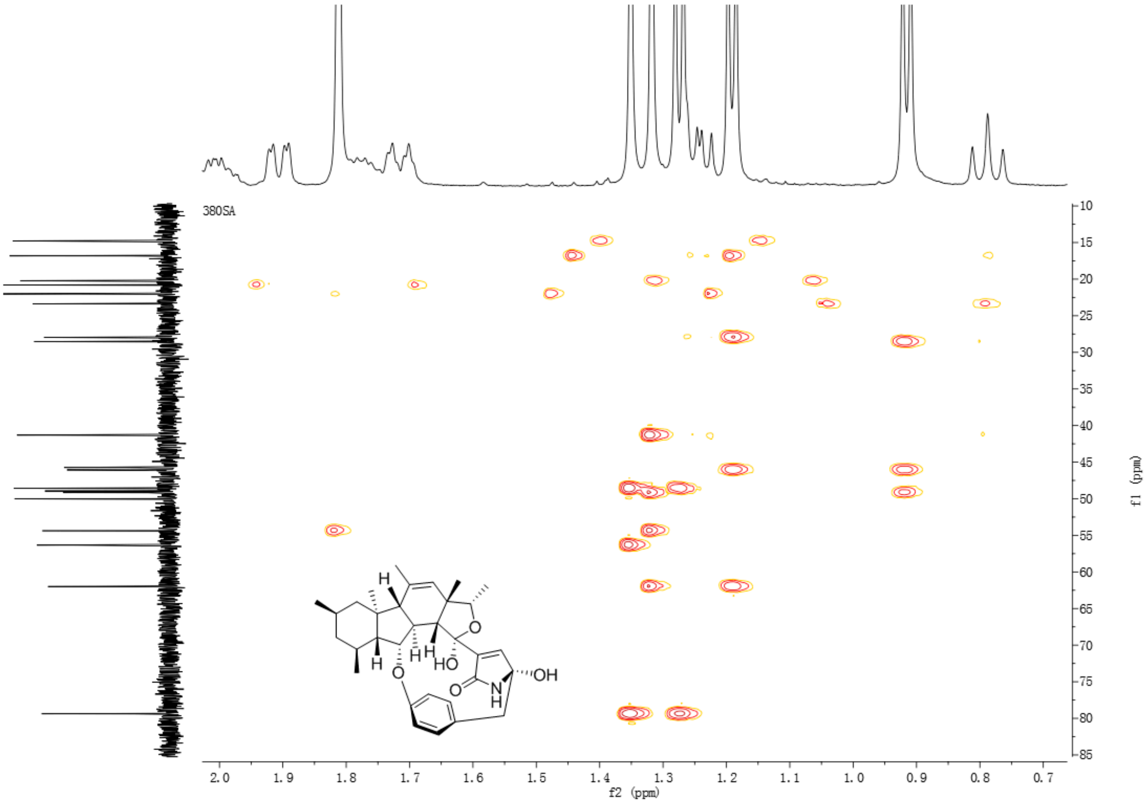

Figure S18. NOESY spectrum of penicipyrroether A (**9**)

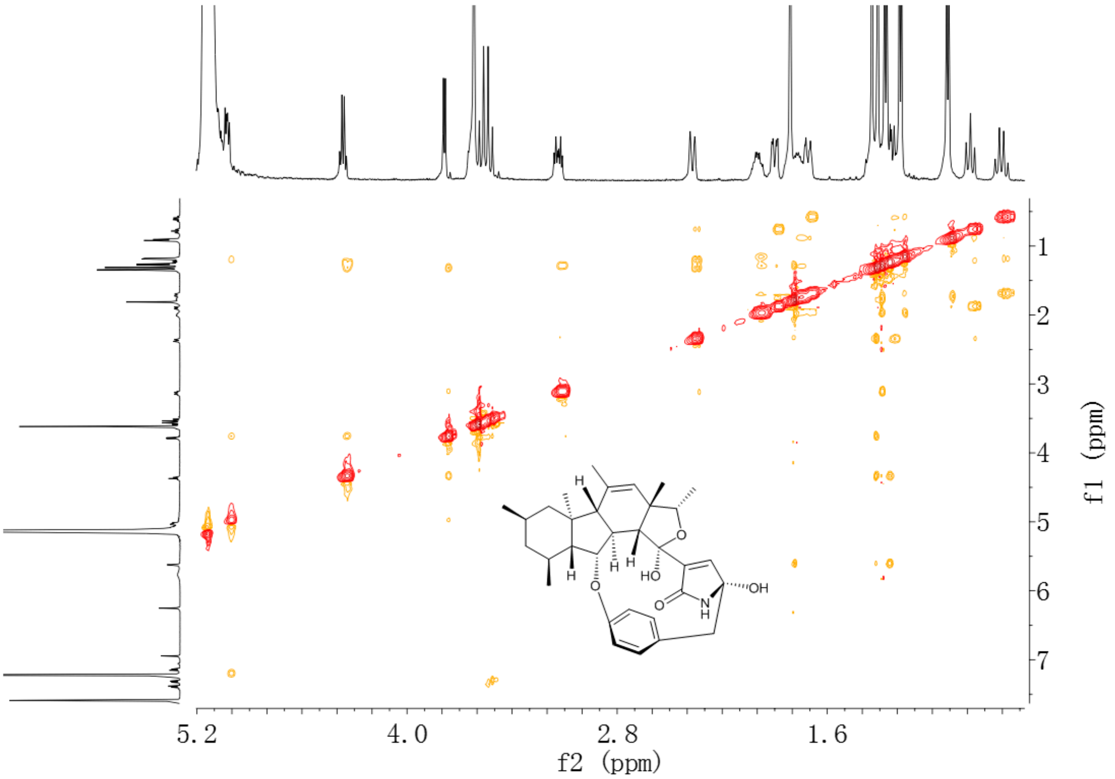

Figure S19. NOESY spectrum of penicipyrroether A (**9**)

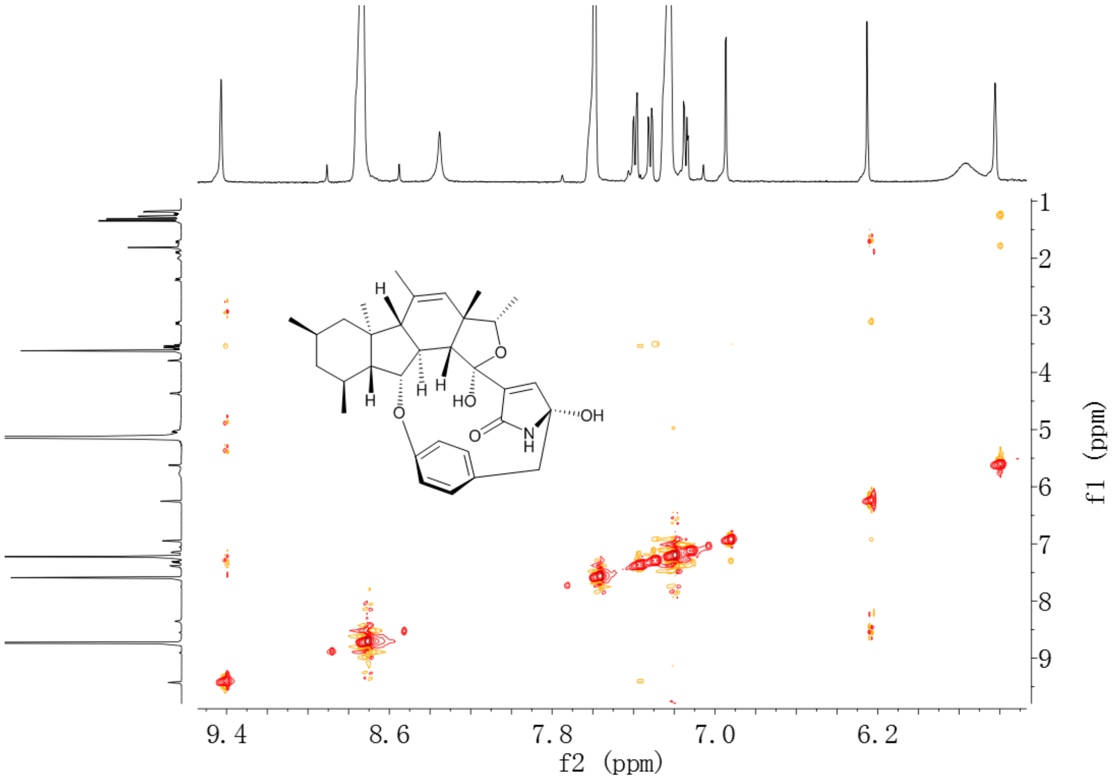

Figure S20. HRESIMS spectrum of penicipyrroether A (**9**)

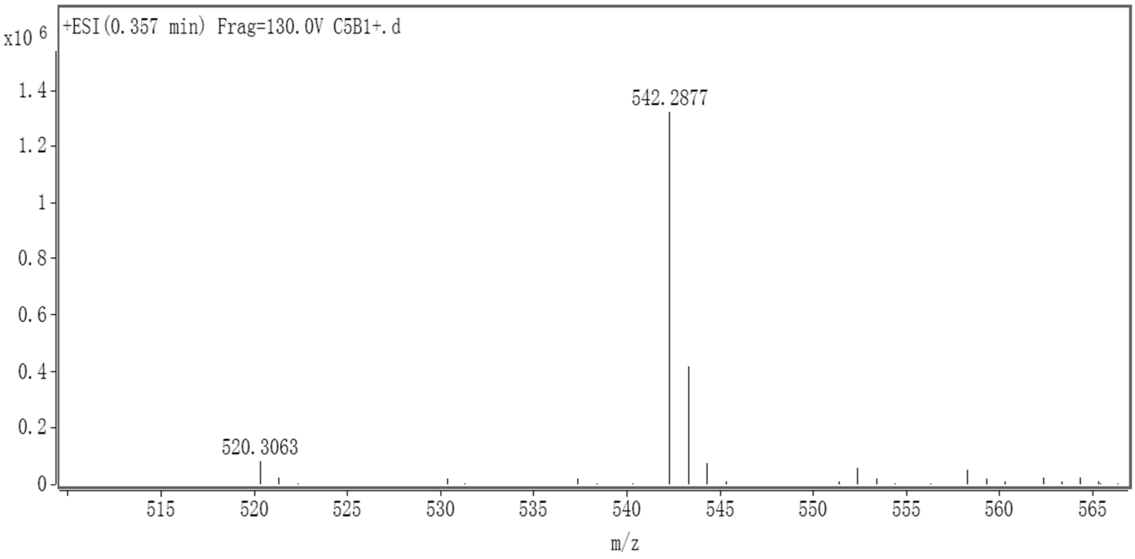

Figure S21. UV spectrum of penicipyrroether A (**9**)

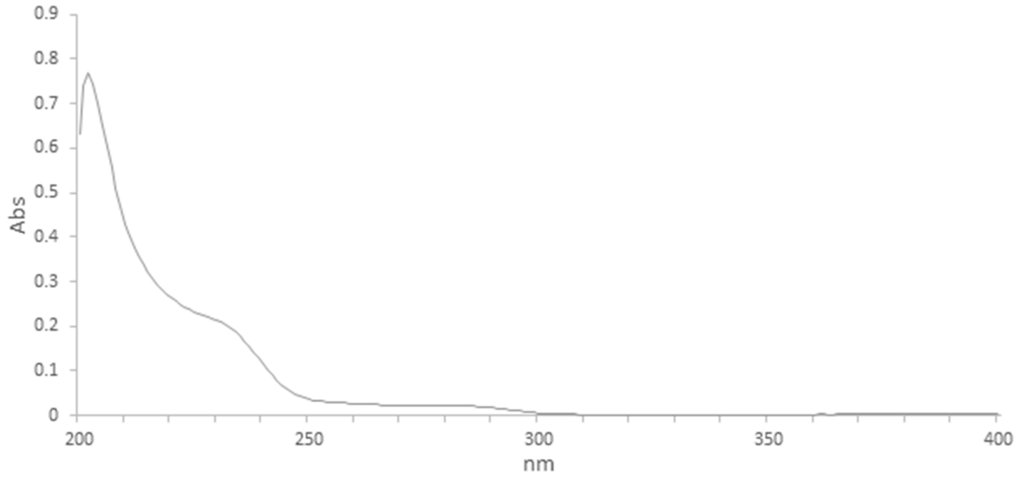

Figure S22. IR spectrum of penicipyrroether A (**9**)

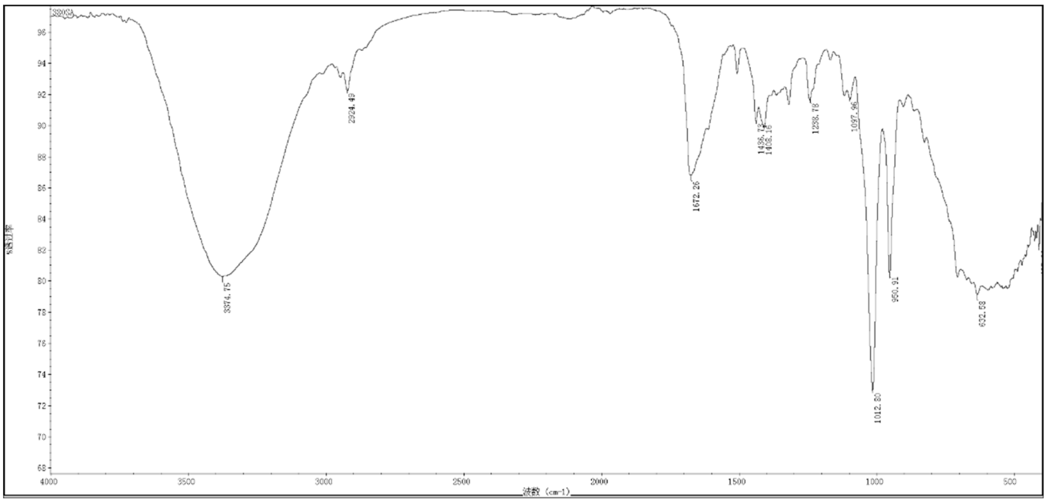

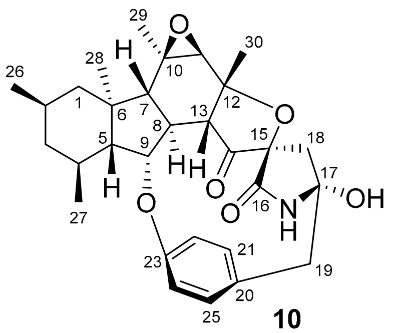

10

^{13}C and ^{1}H NMR data of pyrrospirone J (**10**, in DMSO-d_6)

No.	δ_C, type	δ_H (J in Hz)	No.	δ_C, type	δ_H (J in Hz)
1	47.3, CH_2	βH: 0.81, t (12.3);<br>αH: 1.80, m	17	86.2, C	–
2	27.4, CH	1.82, m	18	40.9, CH_2	1.99, d (12.1);<br>2.45, d (12.1)
3	45.5, CH_2	βH: 0.51, q (12.1); αH: 1.75, m	19	45.0, CH_2	βH: 2.99, d (14.5),<br>αH: 2.69, d (14.5)
4	26.8, CH	1.78, m	20	129.4, C	–
5	59.5, CH	1.22, dd (11.6, 8.8)	21	133.3, CH	6.92, dd (8.3, 2.1)
6	42.5, C	–	22	124.4, CH	6.82, dd (8.3, 2.7)
7	49.9, CH	1.55, d (14.3)	23	158.7, C	–
8	39.2, CH	2.79, m	24	119.7, CH	6.94, dd (8.8, 2.7)
9	85.0, CH	4.82, dd (8.6, 7.0)	25	130.6, CH	6.78, dd (8.8, 2.1)
10	58.8, C	–	26	22.7, CH_3	0.86, d (6.1)
11	63.5, CH	2.46, s	27	19.7, CH_3	1.02, d (6.2)
12	81.3, C	–	28	15.2, CH_3	1.05, s
13	44.5, CH	3.21, d (8.0)	29	21.0, CH_3	1.20, s
14	180.9, C	–	30	26.0, CH_3	1.59, s
15	79.7, C	–	OH-17	–	6.24, s
16	172.1, C	–	NH-16	–	8.76, s

Figure S23. ^{1}H NMR spectrum of pyrrospirone J (**10**)

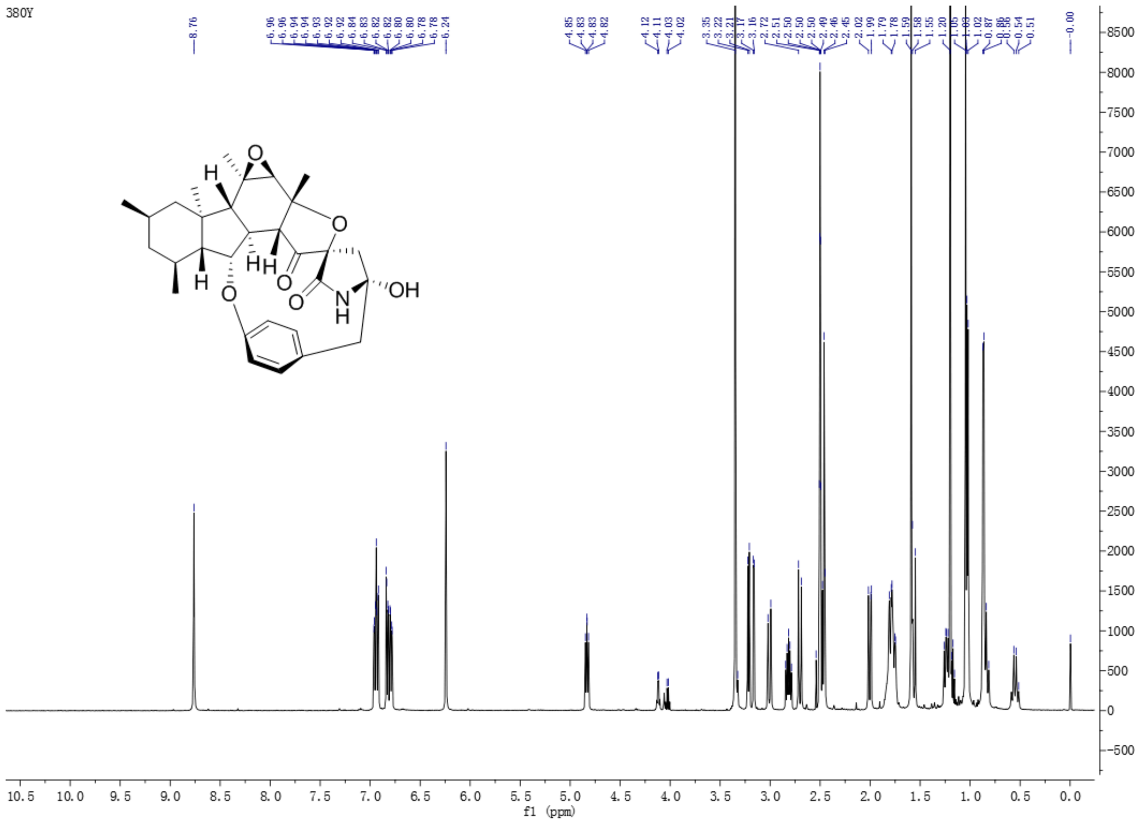

Figure S24. ^{1}H NMR spectrum of pyrrospirone J (**10**)

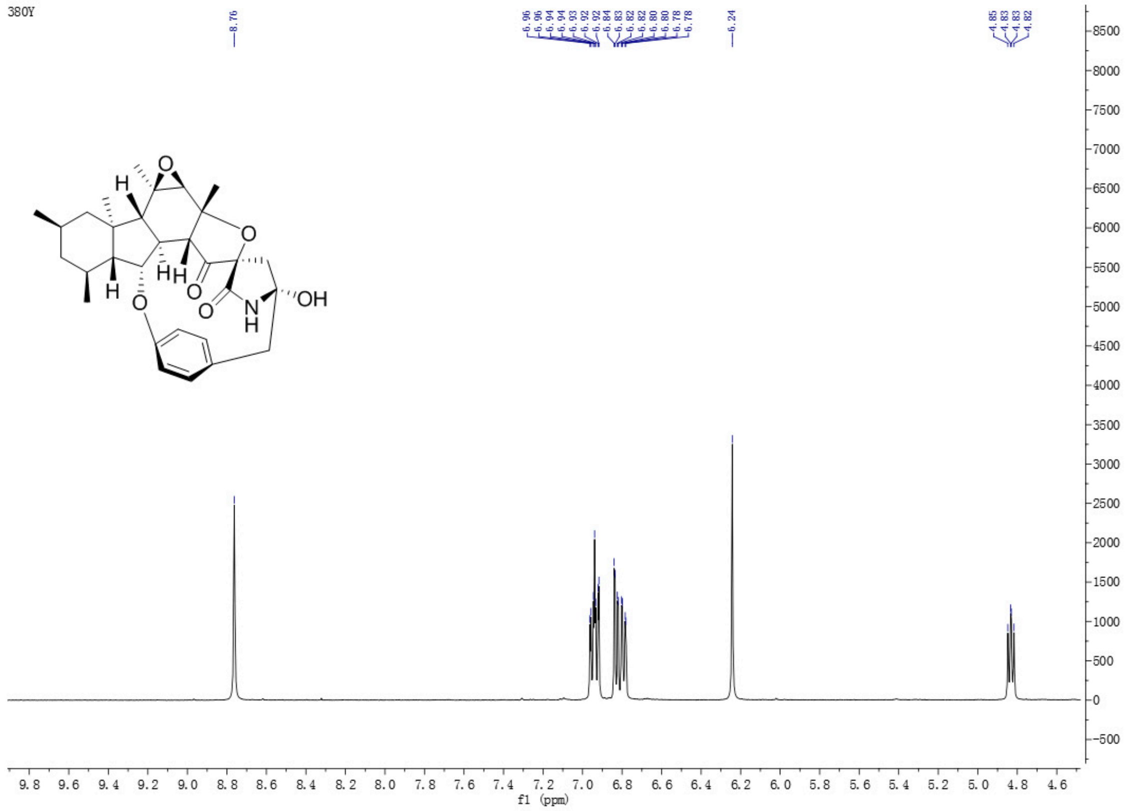

Figure S25. ^{1}H NMR spectrum of pyrrospirone J (**10**)

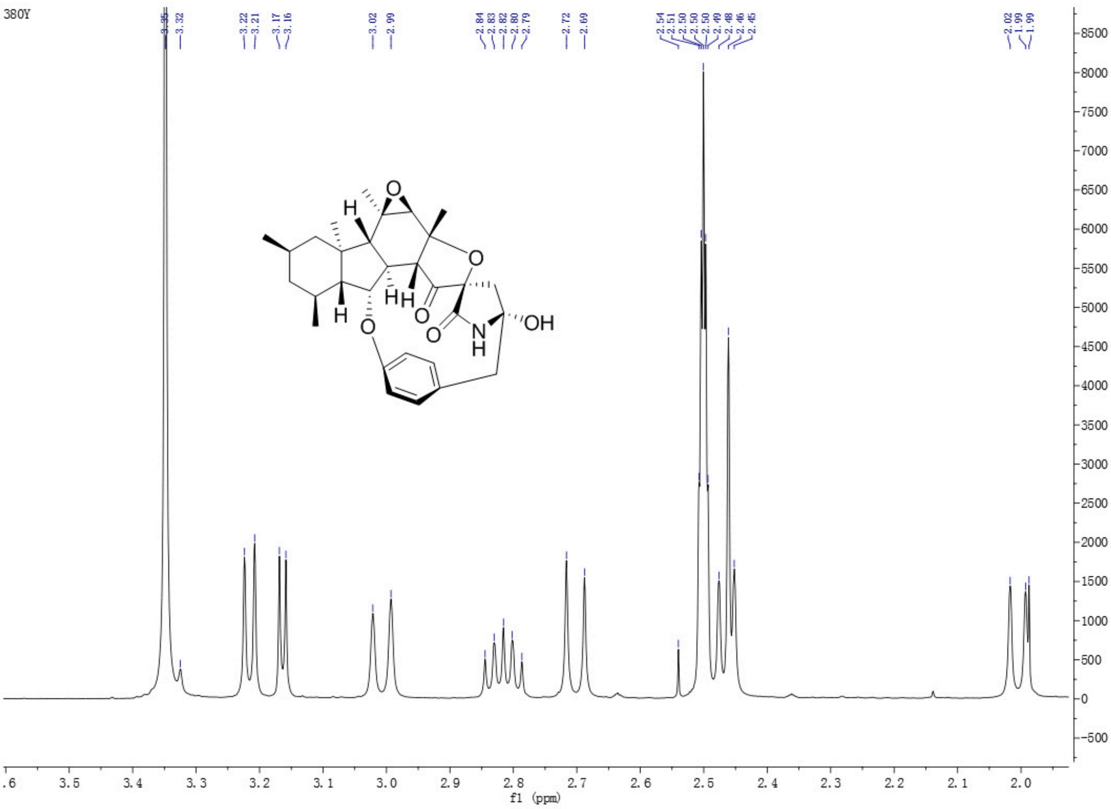

Figure S26. ^{1}H NMR spectrum of pyrrospirone J (**10**)

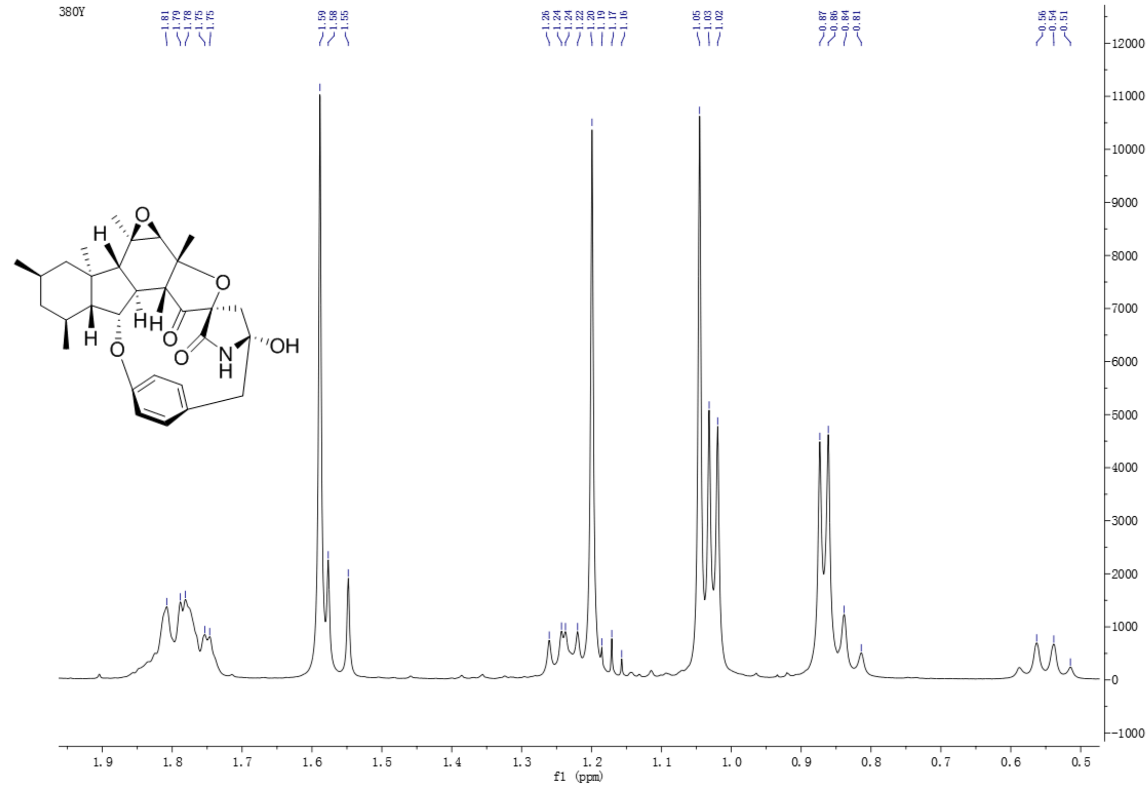

Figure S27. ^{13}C NMR spectrum of pyrrospirone J (**10**)

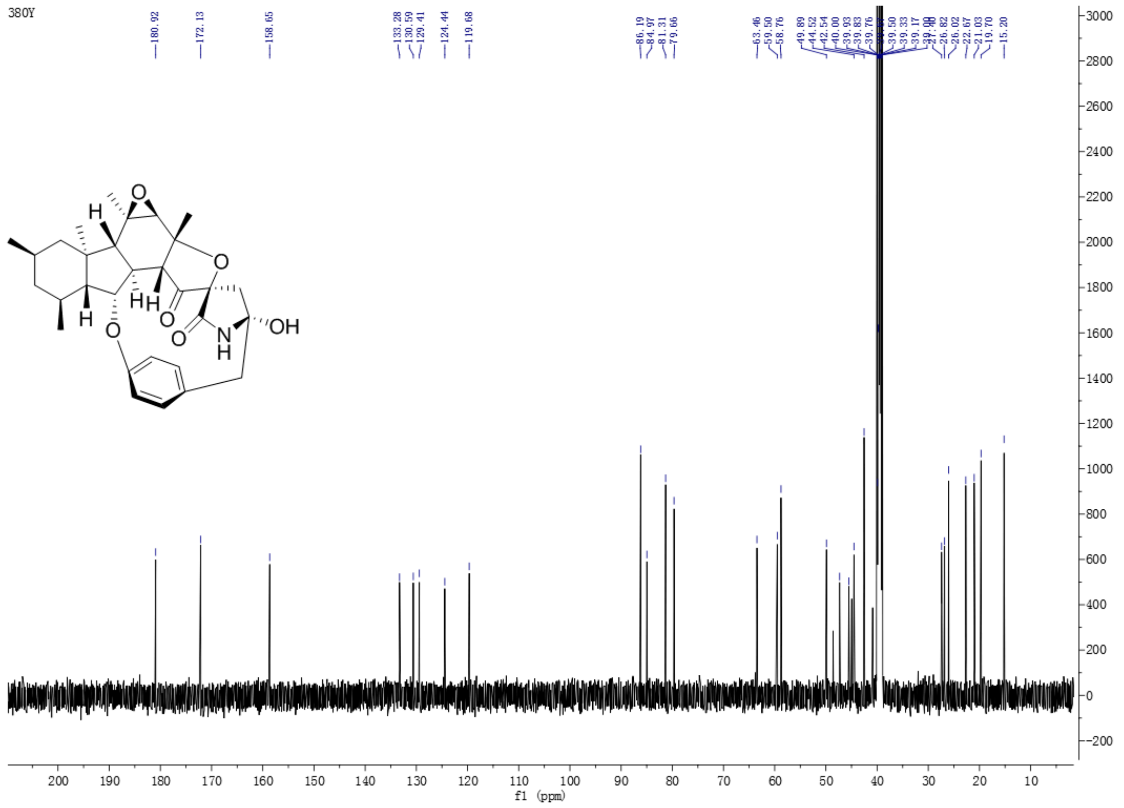

Figure S28. ^{13}C NMR spectrum of pyrrospirone J (**10**)

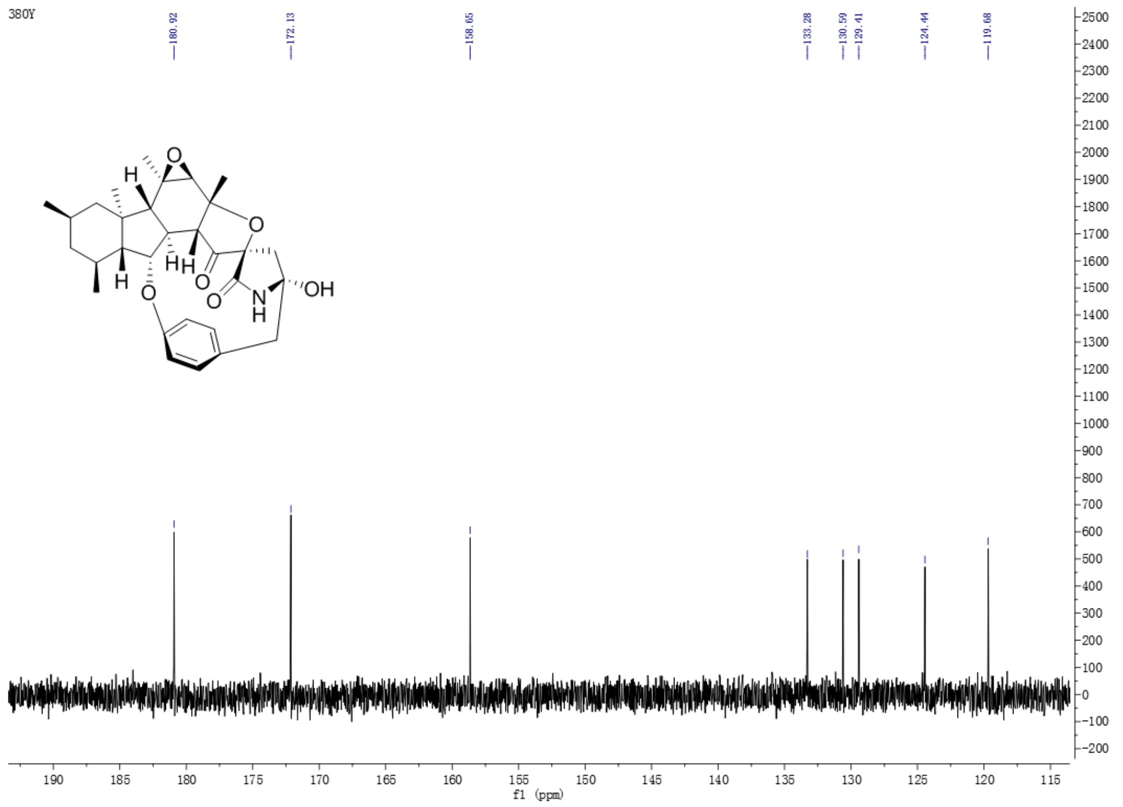

Figure S29. ^{13}C NMR spectrum of pyrrospirone J (**10**)

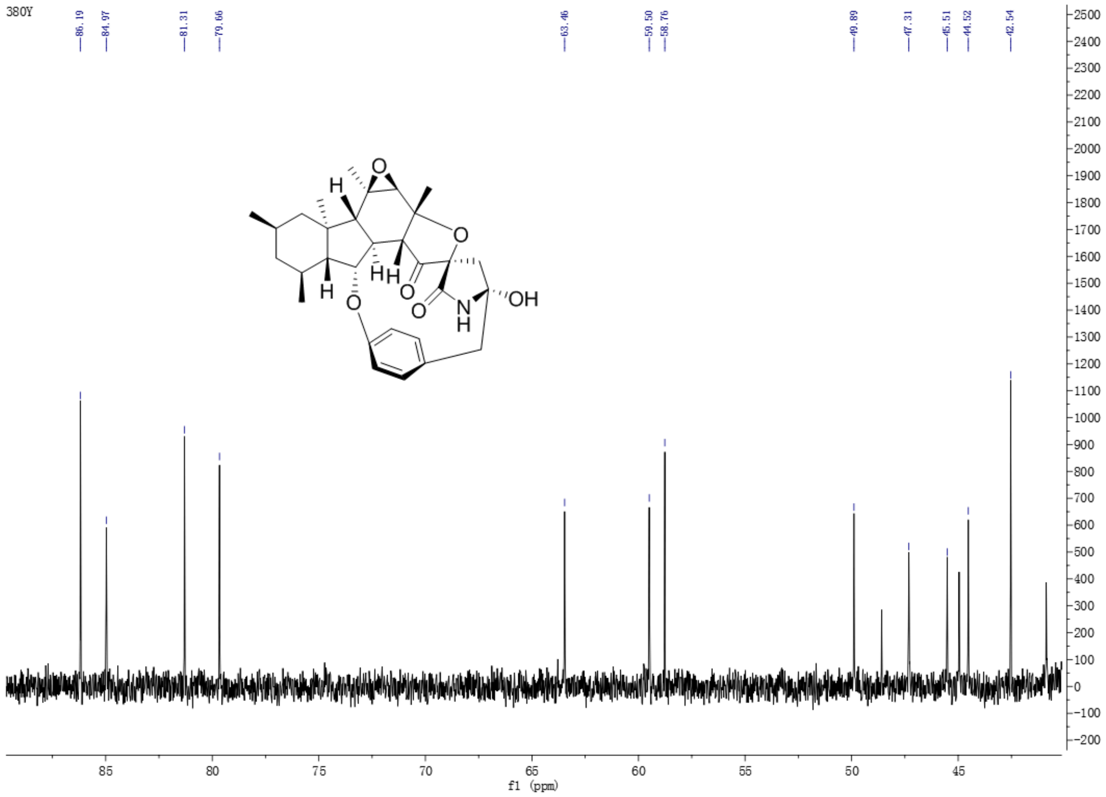

Figure S30. ^{13}C NMR spectrum of pyrrospirone J (**10**)

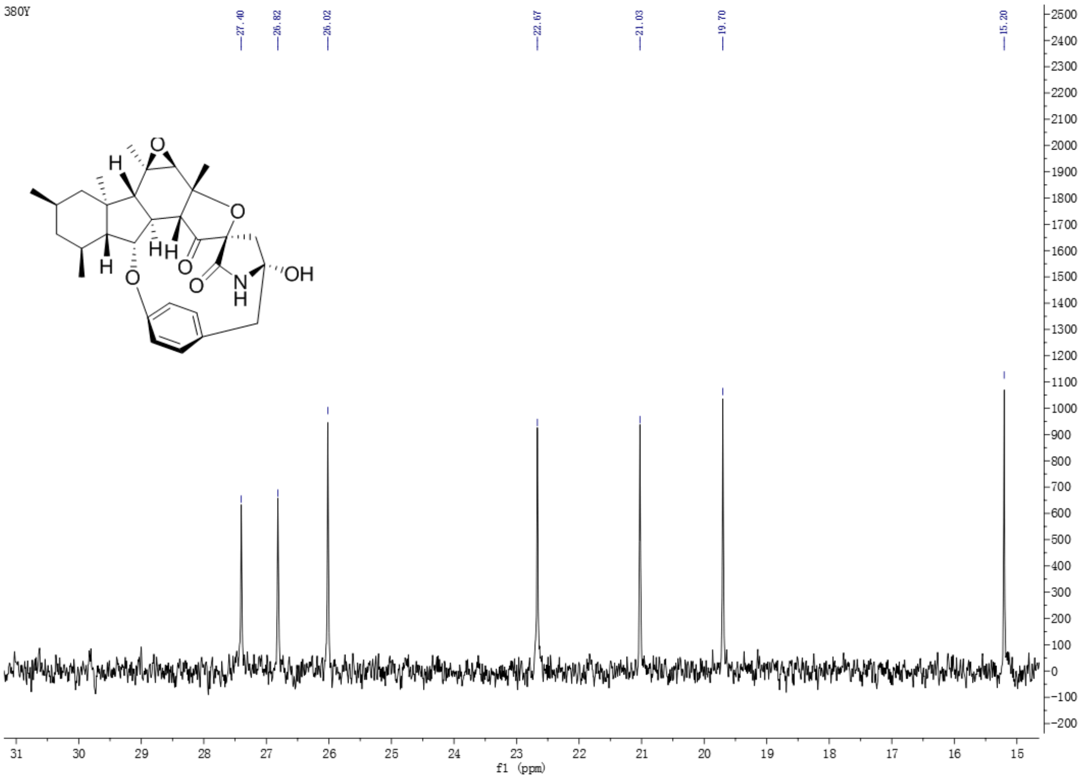

Figure S31. ^{1}H-^{1}H COSY spectrum of pyrrospirone J (**10**)

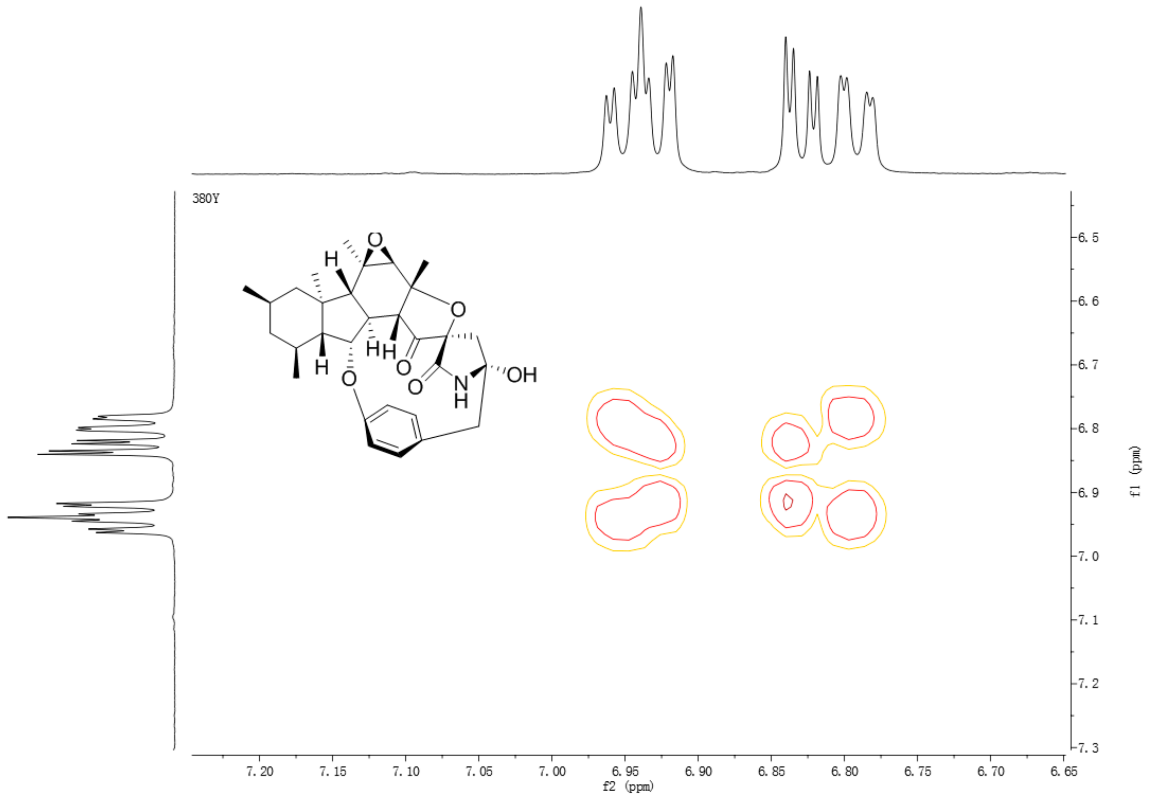

Figure S32. ^{1}H-^{1}H COSY spectrum of pyrrospirone J (**10**)

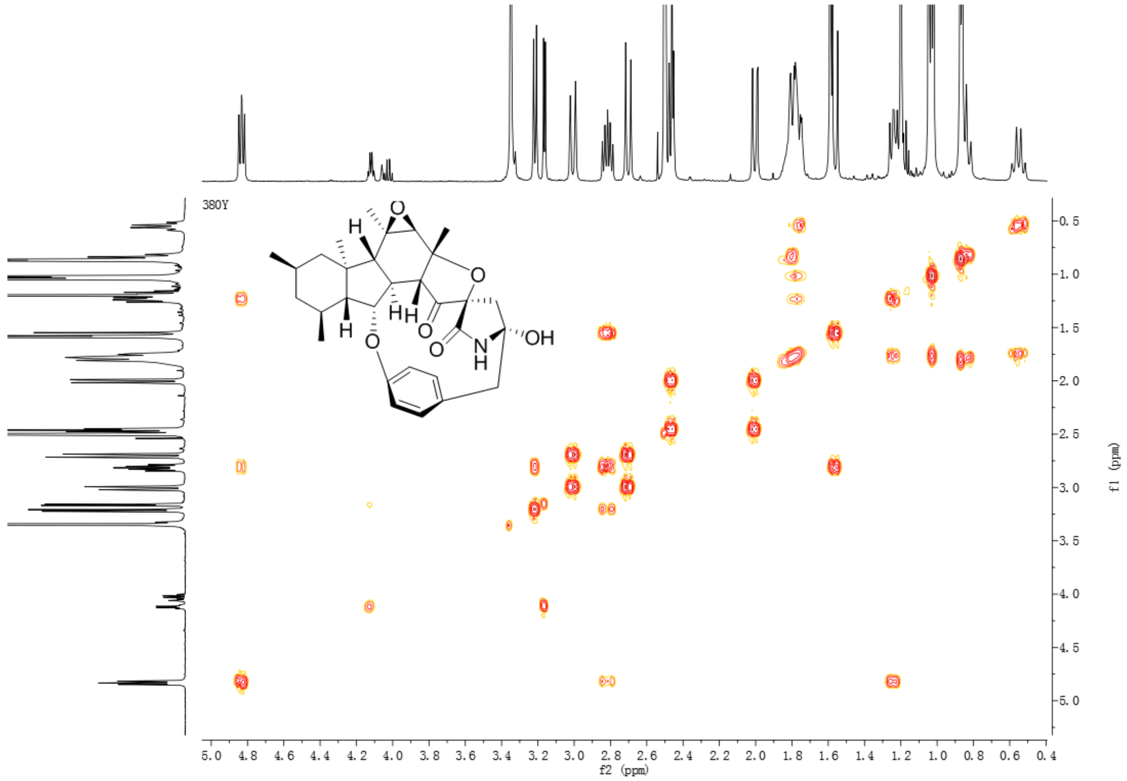

Figure S33. HSQC spectrum of pyrrospirone J (**10**)

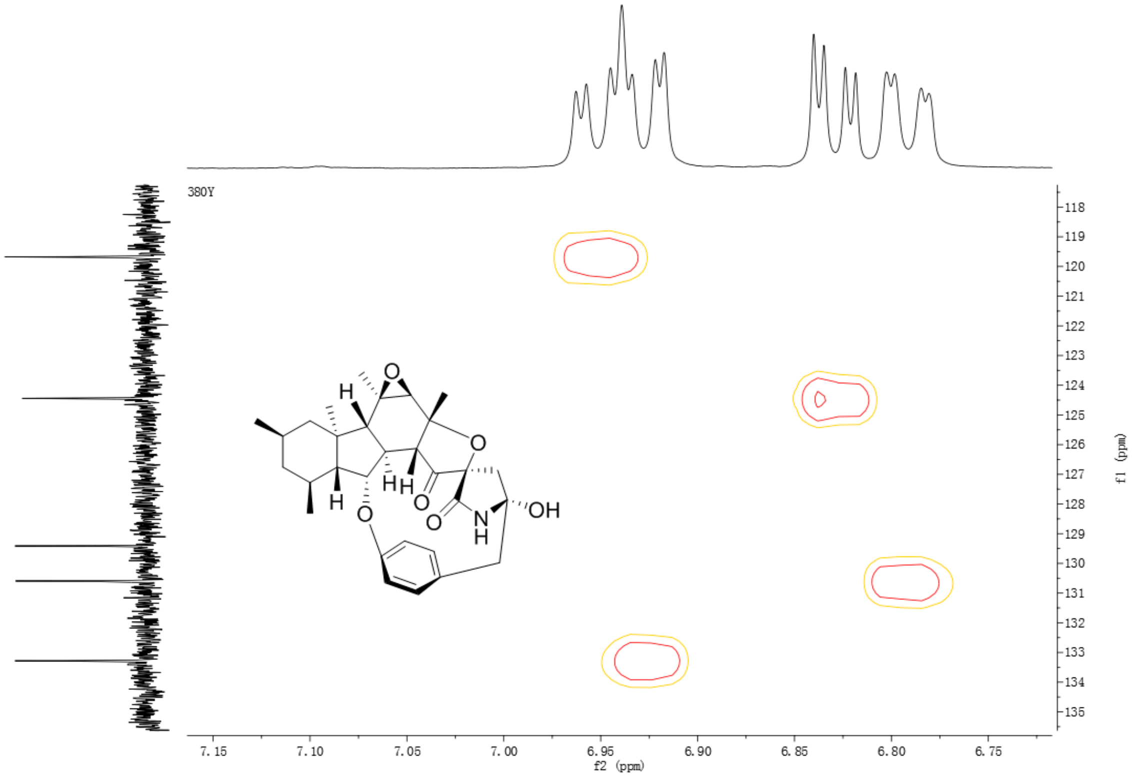

Figure S34. HSQC spectrum of pyrrospirone J (**10**)

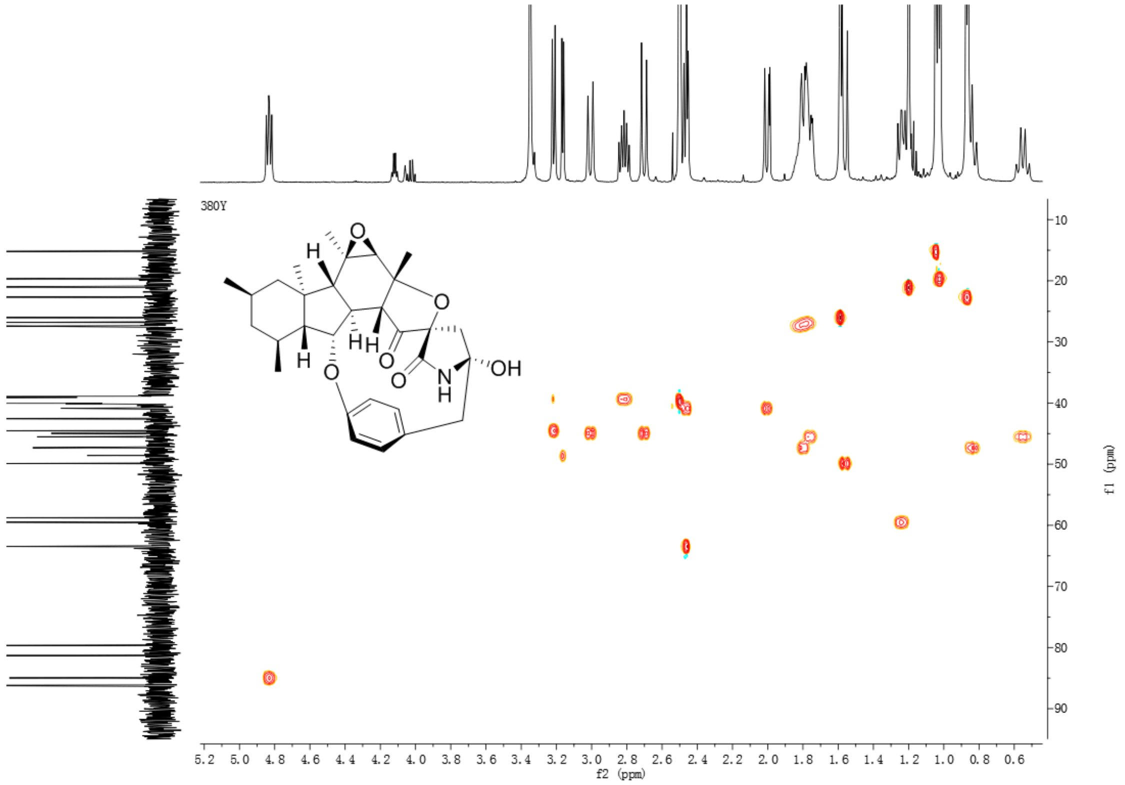

Figure S35. HMBC spectrum of pyrrospirone J (**10**)

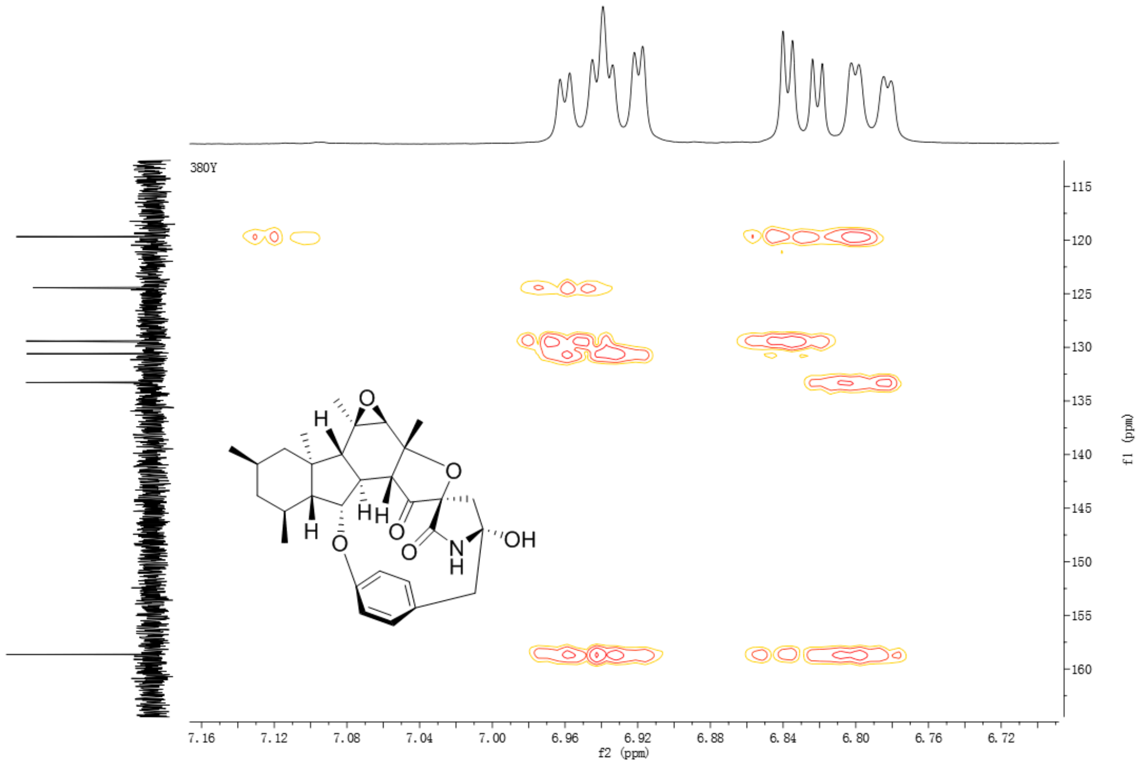

Figure S36. HMBC spectrum of pyrrospirone J (**10**)

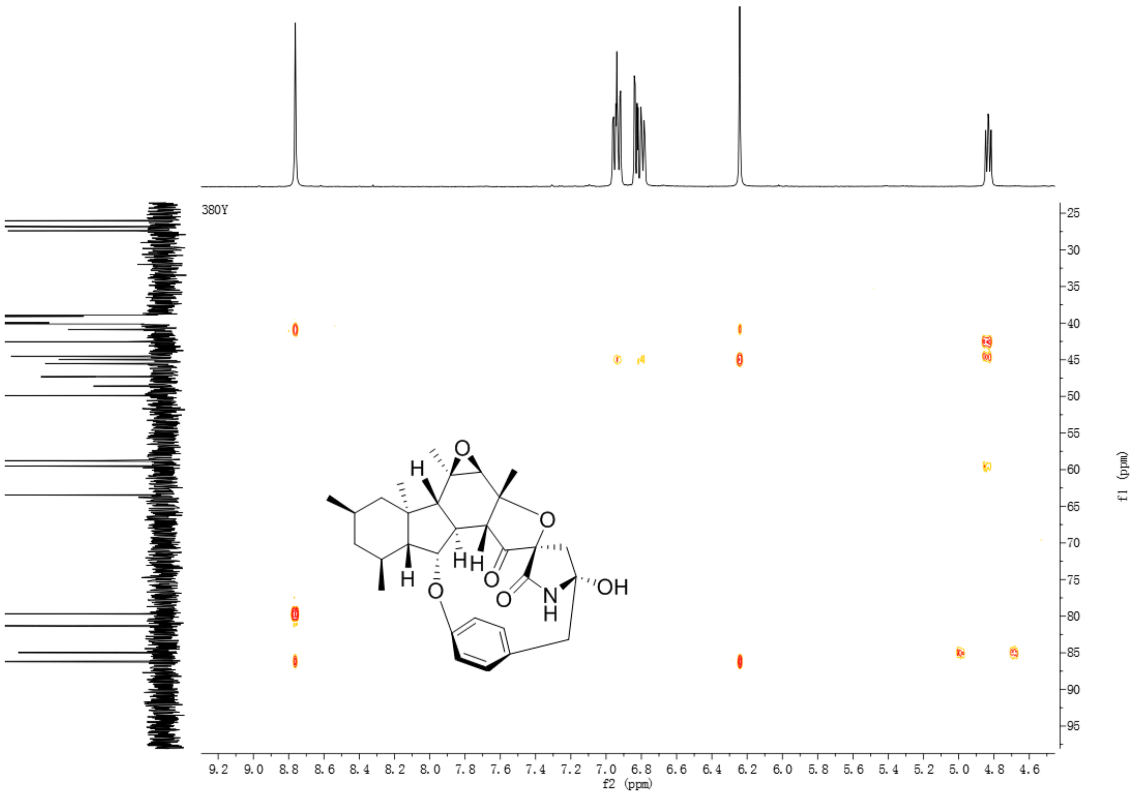

Figure S37. HMBC spectrum of pyrrospirone J (**10**)

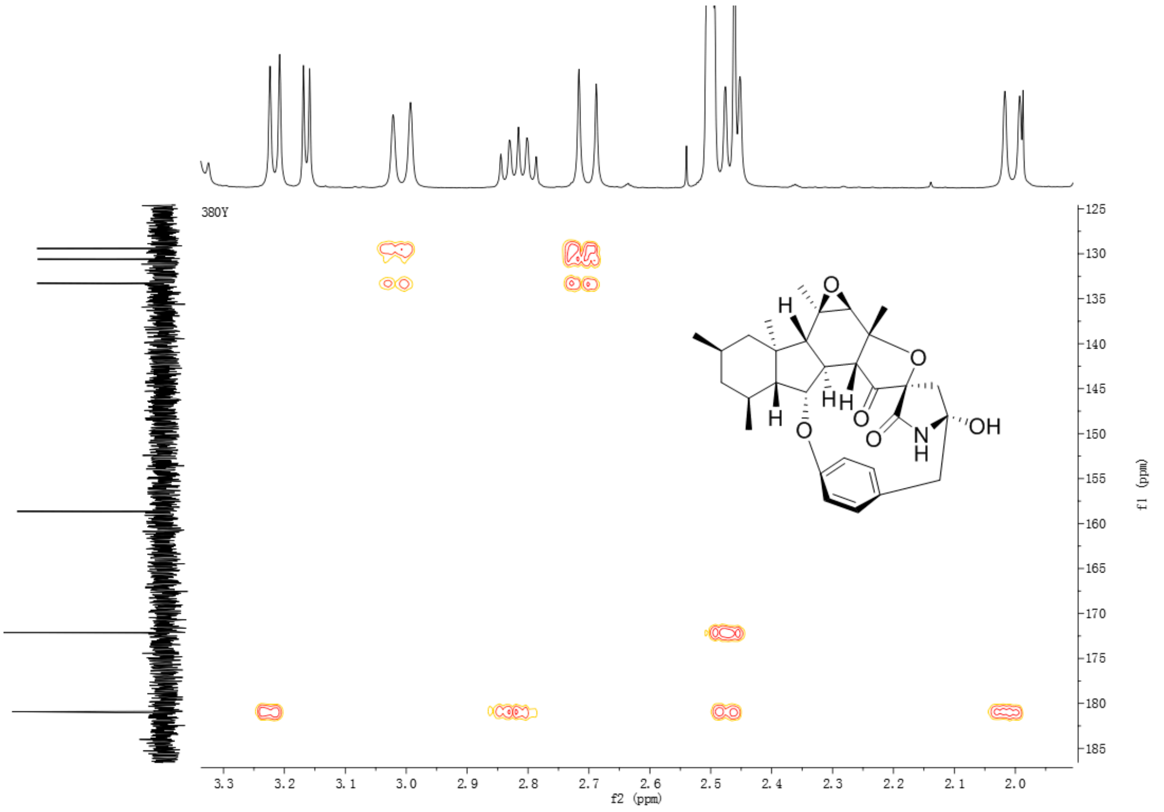

Figure S38. HMBC spectrum of pyrrospirone J (**10**)

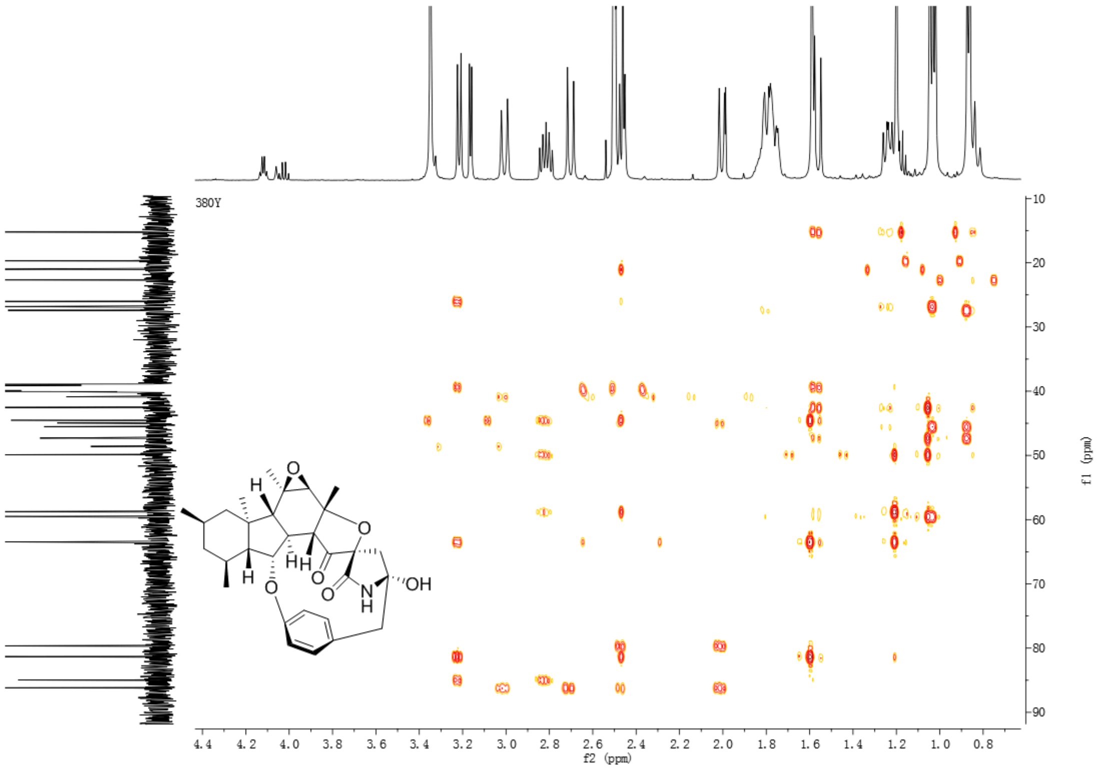

Figure S39. NOESY spectrum of pyrrospirone J (**10**)

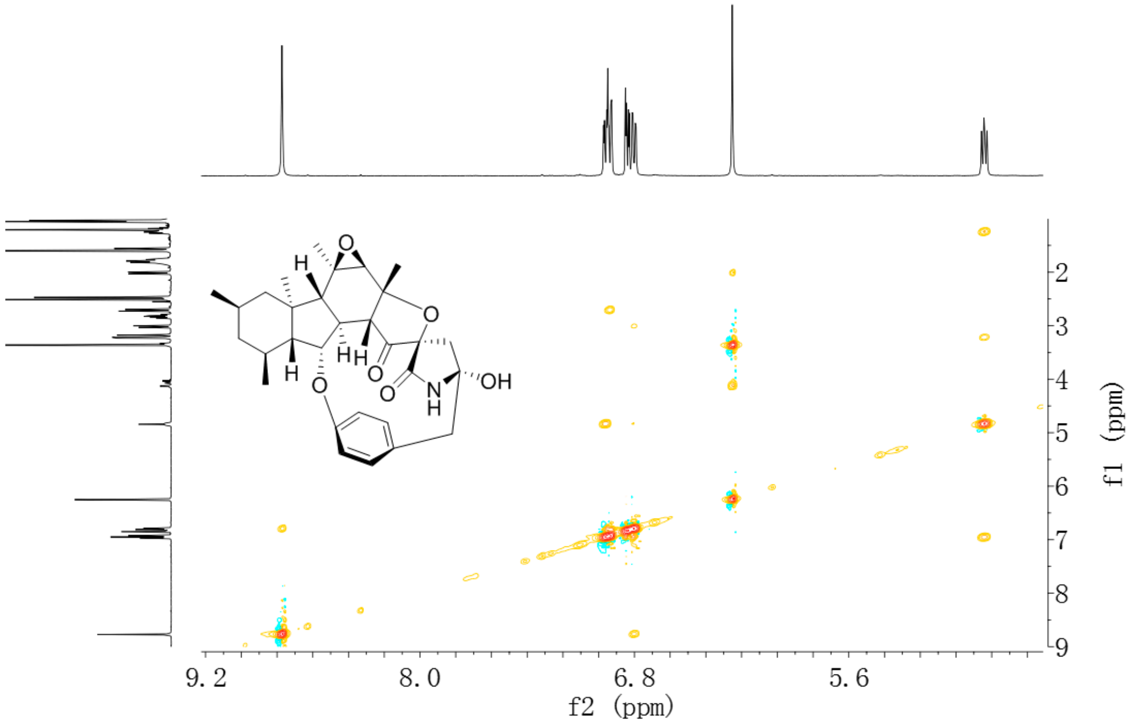

Figure S40. NOESY spectrum of pyrrospirone J (**10**)

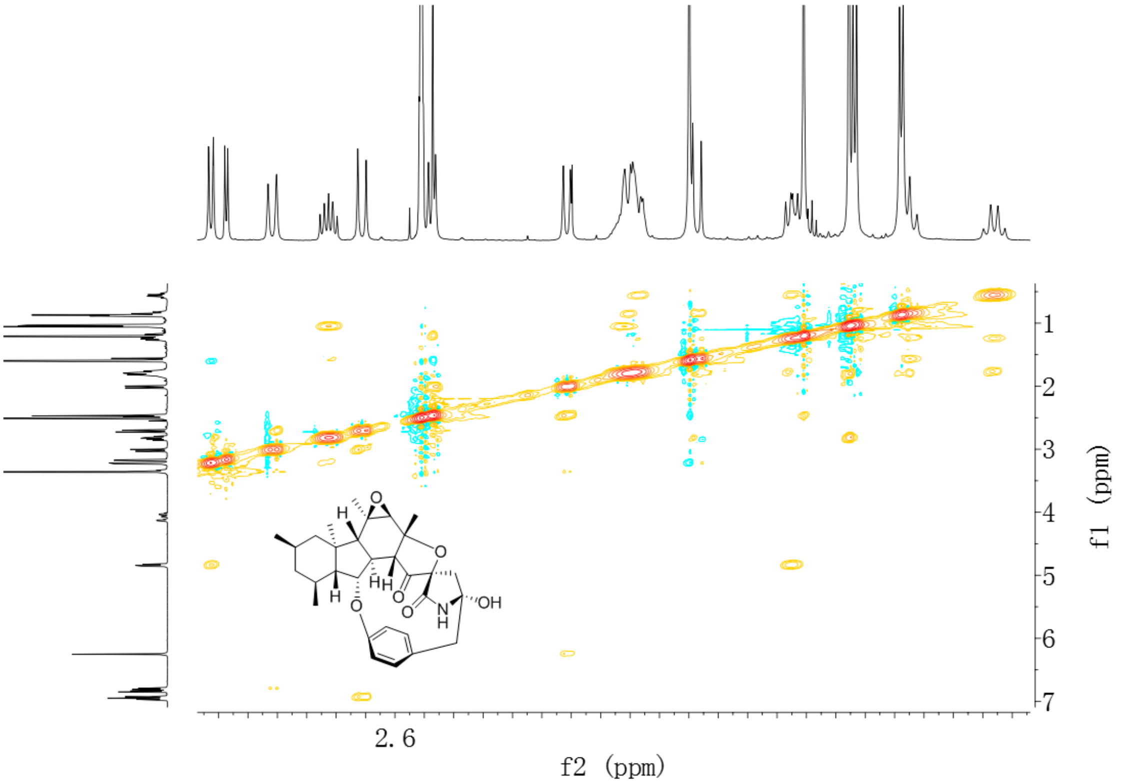

Figure S41. HRESIMS spectrum of pyrrospirone J (**10**)

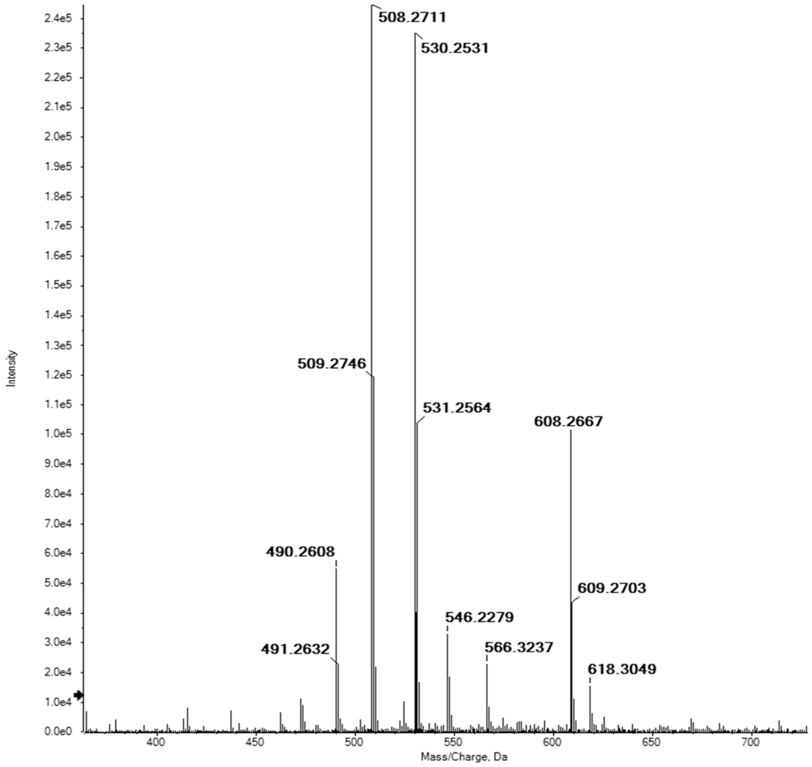

Figure S42. UV spectrum of pyrrospirone J (**10**)

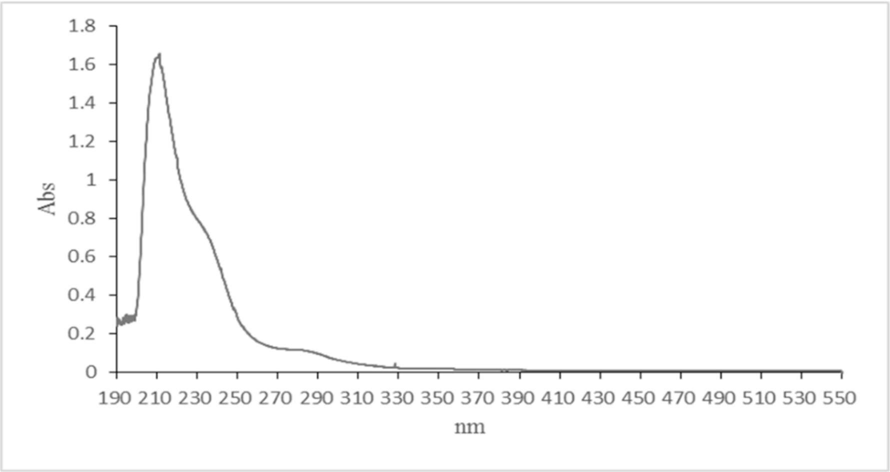

Figure S43. IR spectrum of pyrrospirone J (**10**)

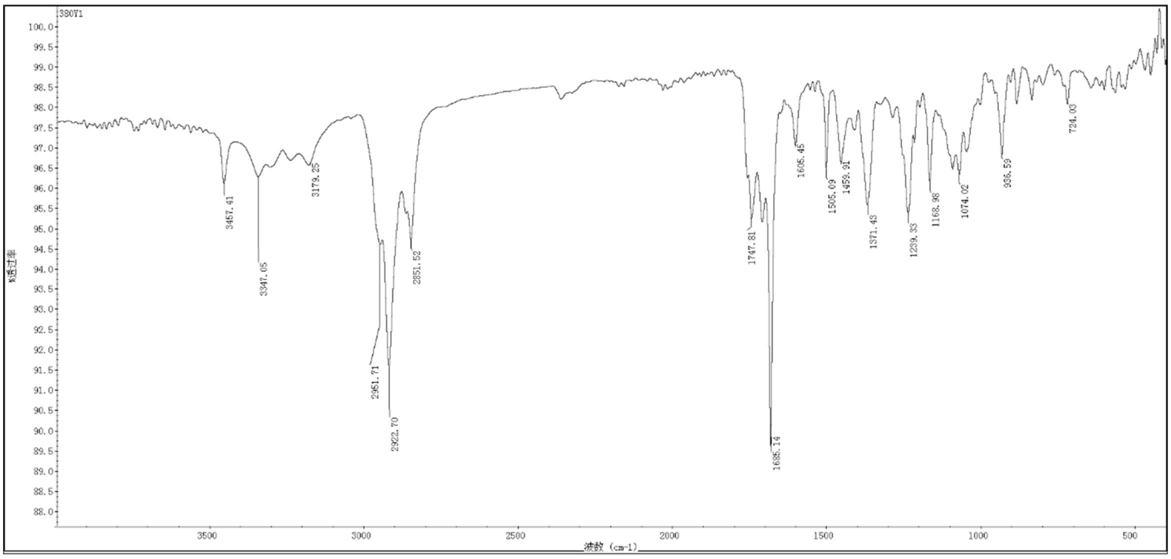

Figure S44. The optimized geometry of conformer (**9-1**) of penicipyrroether A (**9**)

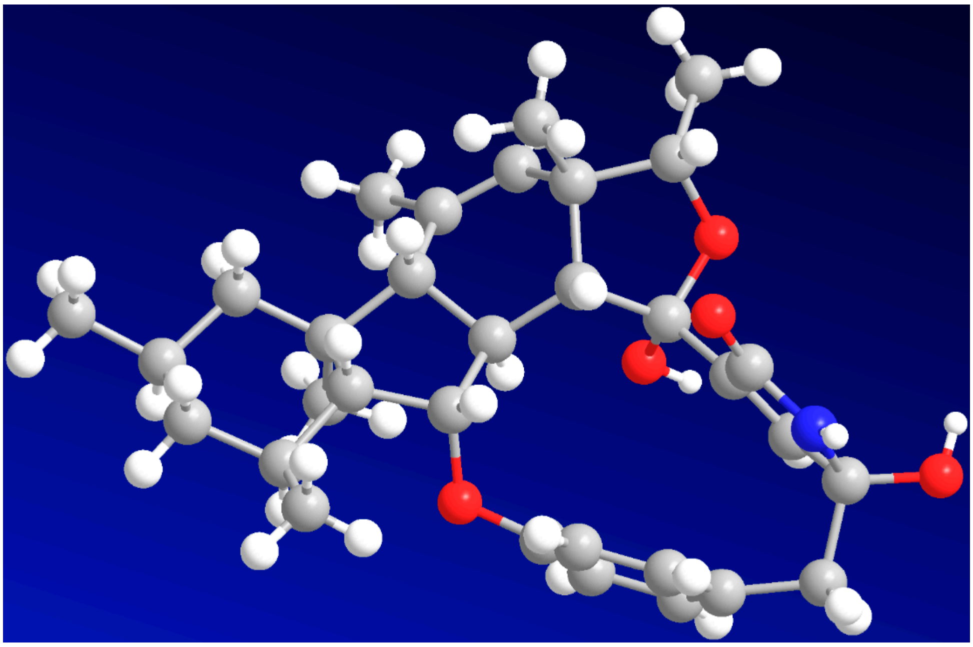

Table S3. Gibbs free energies[a] and equilibrium populations[b] of low-energy conformer of penicipyrroether A (**9**)

Conformer	In MeOH	
	ΔG	*P* (%)/100
**9-1**	0	1

[a] B3LYP/6-31+G(d,p), in kcal/mol; [b] from ΔG values at 298.15K.

Table S4. Cartesian coordinates for the low-energy reoptimized MMFF conformer of penicipyrroether A (**9**) at B3LYP/6-311+G(d,p) level of theory in CH_3OH

**9-1**		Standard Orientation (Ångstroms)			
Center number	Atomic number	Atomic Type	X	Y	Z
1.	8.	0.	-0.828049	-2.184711	-0.241803
2.	8.	0.	1.343517	1.137076	-1.843037
3.	1.	0.	0.743100	1.856627	-2.093147
4.	8.	0.	2.949700	0.733408	2.284654
5.	8.	0.	6.092230	-0.466159	-0.765769
6.	1.	0.	6.172283	0.492638	-0.655383
7.	8.	0.	2.068890	2.708659	-0.346392
8.	7.	0.	4.568263	-0.387165	1.065357
9.	1.	0.	5.065791	-0.754892	1.862743
10.	6.	0.	-2.129327	1.121152	-0.181992
11.	1.	0.	-2.387025	1.390419	0.849646
12.	6.	0.	3.399493	0.327129	1.221119
13.	6.	0.	3.284904	-2.810619	-0.364559
14.	6.	0.	2.839298	0.489740	-0.163410
15.	6.	0.	2.624258	-2.812972	0.869225
16.	1.	0.	3.199392	-2.837840	1.790499
17.	6.	0.	0.503078	-2.518036	-0.241970
18.	6.	0.	-1.210445	-1.033644	0.548072
19.	1.	0.	-0.801533	-1.144845	1.557457
20.	6.	0.	-0.783466	0.327931	-0.108488
21.	1.	0.	-0.502710	0.085126	-1.135559
22.	6.	0.	-1.919034	2.452150	-0.875979
23.	6.	0.	-2.755120	-0.988790	0.582529
24.	1.	0.	-2.992080	-0.484322	1.532981
25.	6.	0.	1.637299	1.349289	-0.483498
26.	6.	0.	4.805023	-0.832049	-0.301750
27.	6.	0.	-3.574019	-2.284829	0.602069
28.	1.	0.	-3.453467	-2.802689	-0.356944
29.	6.	0.	-0.902294	3.186744	-0.387409
30.	1.	0.	-0.721009	4.180251	-0.790646
31.	6.	0.	-5.058243	-1.887871	0.783467
32.	1.	0.	-5.689214	-2.783627	0.713894
33.	1.	0.	-5.184635	-1.507213	1.809489
34.	6.	0.	-3.179423	0.028689	-0.524303
35.	6.	0.	1.240879	-2.669785	0.938510
36.	1.	0.	0.749266	-2.614753	1.903726
37.	6.	0.	0.373587	1.187040	0.478427

38.	1.	0.	0.734794	0.767949	1.421239
39.	6.	0.	-0.016981	2.690417	0.747939
40.	6.	0.	1.124146	-2.719875	-1.476139
41.	1.	0.	0.526722	-2.671962	-2.380268
42.	6.	0.	2.506820	-2.863406	-1.529407
43.	1.	0.	2.997832	-2.912889	-2.498197
44.	6.	0.	3.663986	-0.121436	-1.021309
45.	1.	0.	3.557732	-0.156362	-2.096885
46.	6.	0.	4.728964	-2.387025	-0.453942
47.	1.	0.	5.161870	-2.634924	-1.427324
48.	1.	0.	5.366269	-2.837428	0.315470
49.	6.	0.	-4.648741	0.413102	-0.257152
50.	1.	0.	-5.020084	1.105549	-1.023081
51.	1.	0.	-4.710748	0.947410	0.702919
52.	6.	0.	-3.032024	-0.532542	-1.955657
53.	1.	0.	-3.331246	0.216693	-2.694988
54.	1.	0.	-3.669813	-1.405398	-2.111073
55.	1.	0.	-2.012562	-0.847769	-2.179866
56.	6.	0.	-3.135871	-3.249119	1.712391
57.	1.	0.	-2.111719	-3.595586	1.553100
58.	1.	0.	-3.787585	-4.129377	1.742264
59.	1.	0.	-3.188879	-2.768423	2.697515
60.	6.	0.	1.384216	3.341072	0.738614
61.	1.	0.	1.879104	3.059567	1.679423
62.	6.	0.	-0.657558	2.928149	2.133857
63.	1.	0.	-0.768602	4.001692	2.320479
64.	1.	0.	-0.022546	2.511317	2.922899
65.	1.	0.	-1.649345	2.483649	2.232739
66.	6.	0.	-5.580647	-0.819706	-0.199471
67.	1.	0.	-5.607616	-1.269949	-1.201160
68.	6.	0.	-2.817859	2.960090	-1.971856
69.	1.	0.	-2.791187	2.314689	-2.856939
70.	1.	0.	-2.522486	3.967061	-2.281373
71.	1.	0.	-3.863914	3.001505	-1.646362
72.	6.	0.	1.498955	4.846984	0.556856
73.	1.	0.	2.549827	5.144006	0.615662
74.	1.	0.	0.954151	5.377554	1.343734
75.	1.	0.	1.117986	5.168442	-0.415620
76.	6.	0.	-7.016183	-0.406496	0.150211
77.	1.	0.	-7.690210	-1.270065	0.151489
78.	1.	0.	-7.407610	0.321538	-0.568884

79.	1.	0.	-7.062455	0.051723	1.145645

Figure S45. The optimized geometry of conformers (**10-1**–**10-3**) of pyrrospirone J (**10**)

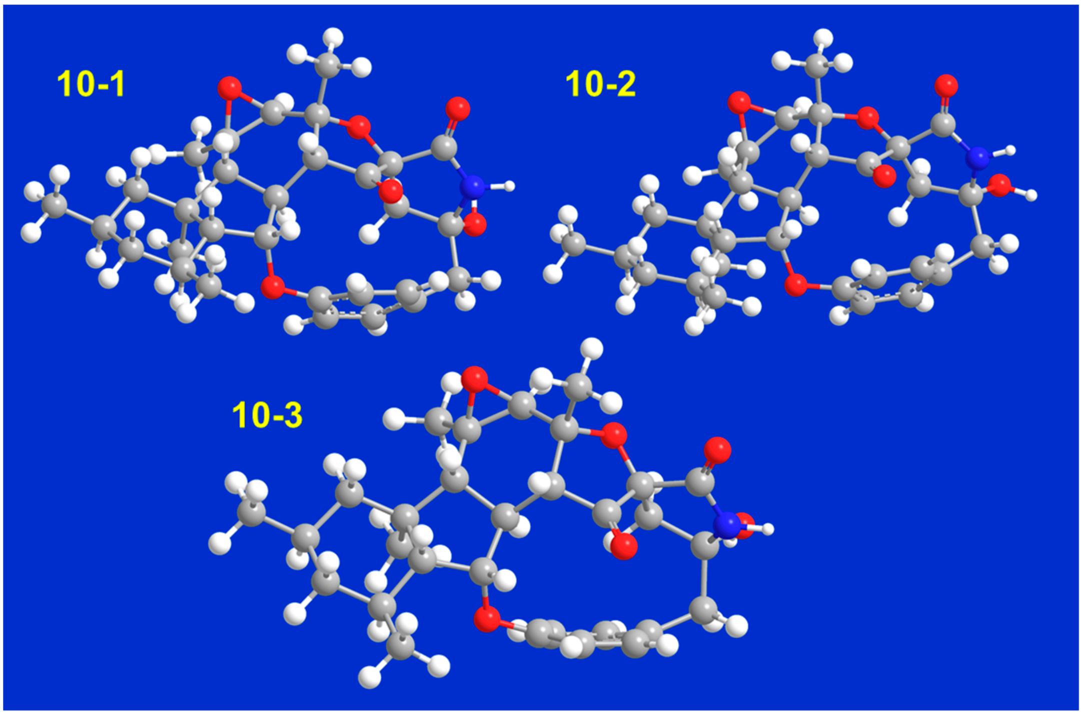

Table S5. Gibbs free energies[a] and equilibrium populations[b] of low-energy conformers of pyrrospirone J (**10**)

Conformers	In MeOH	
	Δ*G*	*P* (%)/100
**10-1**	0.00	0.818
**10-2**	1.24	0.101
**10-3**	1.37	0.081

[a] B3LYP/6-31G(d,p), in kcal/mol; [b] from Δ*G* values at 298.15K.

Table S6. Cartesian coordinates for the low-energy reoptimized MMFF conformers of pyrrospirone J (**10**) at B3LYP/6-311+G(d,p) level of theory in CH_3OH

**10-1**			Standard Orientation (Ångstroms)		
Center number	Atomic number	Atomic Type	X	Y	Z
1.	6.	0.	5.536012	-0.200574	0.281130
2.	6.	0.	5.233085	-1.269199	-0.789420
3.	6.	0.	3.824999	-1.900418	-0.701553
4.	6.	0.	2.806432	-0.755592	-0.653752
5.	6.	0.	3.026261	0.265760	0.509331
6.	6.	0.	4.429594	0.877654	0.332211
7.	6.	0.	1.297954	-1.058411	-0.666418
8.	6.	0.	0.649227	0.267211	-0.118712
9.	6.	0.	1.846436	1.219968	0.169277
10.	6.	0.	-0.332479	1.018043	-1.056450

11.	6.	0.	-0.760861	2.429047	-0.547993
12.	6.	0.	0.089756	3.001124	0.586711
13.	6.	0.	1.381966	2.413439	1.013005
14.	6.	0.	-1.681204	0.343103	-1.234246
15.	6.	0.	-2.694011	1.014312	-0.275191
16.	8.	0.	-2.087590	2.248168	0.052798
17.	6.	0.	-4.027367	1.109095	-1.055878
18.	7.	0.	-4.813138	0.091885	-0.569557
19.	6.	0.	-4.457596	-0.461189	0.735964
20.	6.	0.	-3.120551	0.285694	1.020333
21.	6.	0.	6.912828	0.435128	0.050661
22.	6.	0.	3.579117	-2.858131	-1.874958
23.	1.	0.	2.972368	-0.174791	-1.575970
24.	1.	0.	2.121109	1.677015	-0.789782
25.	8.	0.	1.321755	3.641323	0.239718
26.	6.	0.	-0.901671	3.429398	-1.695812
27.	1.	0.	0.129949	1.093448	-2.045764
28.	8.	0.	-1.955906	-0.468126	-2.087145
29.	8.	0.	-4.292957	1.872610	-1.962151
30.	8.	0.	-5.467199	-0.133790	1.687349
31.	6.	0.	-0.795726	-2.589399	1.621369
32.	6.	0.	-2.179603	-2.617579	1.778810
33.	6.	0.	-3.031346	-2.605487	0.660110
34.	6.	0.	-2.464509	-2.856140	-0.591571
35.	6.	0.	-1.078891	-2.864682	-0.759853
36.	6.	0.	-0.255200	-2.578748	0.329756
37.	6.	0.	2.900837	-0.384561	1.906377
38.	6.	0.	1.838531	2.621381	2.439353
39.	1.	0.	0.141398	0.034904	0.823852
40.	8.	0.	1.067196	-2.220096	0.174847
41.	6.	0.	-4.423287	-2.039171	0.788378
42.	1.	0.	5.565773	-0.702616	1.258156
43.	1.	0.	5.340634	-0.806529	-1.783175
44.	1.	0.	5.992826	-2.059796	-0.739294
45.	1.	0.	3.751351	-2.485247	0.222883
46.	1.	0.	4.454594	1.454932	-0.603814
47.	1.	0.	4.650359	1.588348	1.139020
48.	1.	0.	0.940629	-1.285327	-1.676095
49.	1.	0.	-0.513692	3.542539	1.317583
50.	1.	0.	-5.730806	-0.069253	-0.965285
51.	1.	0.	-3.322963	1.079428	1.745963

52.	1.	0.	-2.347999	-0.351328	1.438451
53.	1.	0.	6.949161	0.956028	-0.913709
54.	1.	0.	7.150001	1.165772	0.831681
55.	1.	0.	7.705028	-0.321367	0.049145
56.	1.	0.	2.615504	-3.365248	-1.775466
57.	1.	0.	4.358444	-3.626634	-1.918398
58.	1.	0.	3.590042	-2.324054	-2.833313
59.	1.	0.	-1.588970	3.039346	-2.452398
60.	1.	0.	-1.316275	4.367788	-1.317629
61.	1.	0.	0.071159	3.636867	-2.148974
62.	1.	0.	-5.604602	0.824270	1.651117
63.	1.	0.	-0.133945	-2.481084	2.474836
64.	1.	0.	-2.602866	-2.513989	2.775234
65.	1.	0.	-3.100252	-2.914941	-1.469185
66.	1.	0.	-0.657563	-2.949078	-1.754922
67.	1.	0.	3.251039	0.299397	2.683546
68.	1.	0.	1.874623	-0.672352	2.141122
69.	1.	0.	3.503395	-1.291522	1.982724
70.	1.	0.	2.928741	2.689886	2.499465
71.	1.	0.	1.512297	1.804265	3.088808
72.	1.	0.	1.419763	3.555810	2.821414
73.	1.	0.	-5.088133	-2.416568	0.004690
74.	1.	0.	-4.864929	-2.308671	1.751234

**10-2**			Standard Orientation (Ångstroms)		
Center number	Atom number	Atomic type	X	Y	Z
1.	6.	0.	5.534179	-0.206245	0.281730
2.	6.	0.	5.230600	-1.271881	-0.791631
3.	6.	0.	3.821537	-1.901267	-0.706407
4.	6.	0.	2.804518	-0.755165	-0.656807
5.	6.	0.	3.024777	0.262901	0.509319
6.	6.	0.	4.429110	0.873241	0.334749
7.	6.	0.	1.295535	-1.055416	-0.672195
8.	6.	0.	0.648718	0.269244	-0.120707
9.	6.	0.	1.846470	1.219689	0.171236
10.	6.	0.	-0.330970	1.023212	-1.057349
11.	6.	0.	-0.759237	2.433282	-0.546956
12.	6.	0.	0.089946	3.001079	0.591249
13.	6.	0.	1.381197	2.411109	1.017841
14.	6.	0.	-1.678837	0.347453	-1.234381
15.	6.	0.	-2.693008	1.019823	-0.277043
16.	8.	0.	-2.087358	2.254068	0.048067

17.	6.	0.	-4.024459	1.104360	-1.060734
18.	7.	0.	-4.778360	0.045250	-0.608635
19.	6.	0.	-4.451784	-0.450250	0.728984
20.	6.	0.	-3.125109	0.297569	1.020121
21.	6.	0.	6.912010	0.428110	0.053723
22.	6.	0.	3.575357	-2.856059	-1.882136
23.	1.	0.	2.972238	-0.172121	-1.577284
24.	1.	0.	2.122606	1.679456	-0.786115
25.	8.	0.	1.323737	3.641179	0.248480
26.	6.	0.	-0.894052	3.436657	-1.693068
27.	1.	0.	0.132067	1.099497	-2.046334
28.	8.	0.	-1.949297	-0.466121	-2.086922
29.	8.	0.	-4.304964	1.876391	-1.953937
30.	8.	0.	-5.397178	0.033024	1.684212
31.	6.	0.	-0.791284	-2.589995	1.620047
32.	6.	0.	-2.173687	-2.616849	1.786138
33.	6.	0.	-3.032466	-2.604298	0.672952
34.	6.	0.	-2.474771	-2.848972	-0.583909
35.	6.	0.	-1.090138	-2.857501	-0.760309
36.	6.	0.	-0.258967	-2.576075	0.325137
37.	6.	0.	2.897772	-0.390993	1.904566
38.	6.	0.	1.835426	2.614147	2.445746
39.	1.	0.	0.139813	0.035211	0.820862
40.	8.	0.	1.062821	-2.220663	0.164909
41.	6.	0.	-4.420754	-2.031300	0.817671
42.	1.	0.	5.562660	-0.710762	1.257525
43.	1.	0.	5.339594	-0.806983	-1.784199
44.	1.	0.	5.989158	-2.063725	-0.742844
45.	1.	0.	3.746373	-2.488003	0.216737
46.	1.	0.	4.455506	1.452897	-0.599757
47.	1.	0.	4.650129	1.581606	1.143509
48.	1.	0.	0.938397	-1.278893	-1.682705
49.	1.	0.	-0.514375	3.540517	1.322715
50.	1.	0.	-5.705752	-0.080569	-0.995153
51.	1.	0.	-3.355912	1.092056	1.733510
52.	1.	0.	-2.354114	-0.335423	1.446687
53.	1.	0.	6.949713	0.951232	-0.909393
54.	1.	0.	7.149555	1.156646	0.836587
55.	1.	0.	7.703239	-0.329422	0.050999
56.	1.	0.	2.610762	-3.361747	-1.784783
57.	1.	0.	4.353490	-3.625748	-1.926505

58.	1.	0.	3.588087	-2.319910	-2.839308
59.	1.	0.	-1.578308	3.048742	-2.453520
60.	1.	0.	-1.310453	4.373896	-1.314157
61.	1.	0.	0.080792	3.644909	-2.141617
62.	1.	0.	-6.200274	-0.502978	1.619369
63.	1.	0.	-0.124177	-2.483817	2.469522
64.	1.	0.	-2.589671	-2.513800	2.785660
65.	1.	0.	-3.115740	-2.902791	-1.457904
66.	1.	0.	-0.675313	-2.937633	-1.758347
67.	1.	0.	3.248750	0.290470	2.683526
68.	1.	0.	1.870854	-0.677104	2.138284
69.	1.	0.	3.498661	-1.299270	1.978832
70.	1.	0.	2.925639	2.681421	2.508115
71.	1.	0.	1.506886	1.795598	3.092234
72.	1.	0.	1.416732	3.547802	2.829651
73.	1.	0.	-5.104057	-2.431158	0.059000
74.	1.	0.	-4.822870	-2.287545	1.803125

**10-3**			Standard Orientation (Ångstroms)		
Center number	Atomic number	Atomic type	X	Y	Z
1.	6.	0.	5.534594	-0.208324	0.283031
2.	6.	0.	5.230924	-1.272085	-0.792160
3.	6.	0.	3.821239	-1.900379	-0.709052
4.	6.	0.	2.805230	-0.753421	-0.658601
5.	6.	0.	3.025353	0.262673	0.509292
6.	6.	0.	4.430401	0.872014	0.336878
7.	6.	0.	1.295966	-1.052223	-0.675967
8.	6.	0.	0.649904	0.272066	-0.122871
9.	6.	0.	1.848143	1.221020	0.171719
10.	6.	0.	-0.328976	1.028656	-1.058152
11.	6.	0.	-0.758774	2.436312	-0.542865
12.	6.	0.	0.091490	3.002173	0.595342
13.	6.	0.	1.383153	2.411416	1.020019
14.	6.	0.	-1.676417	0.352882	-1.237889
15.	6.	0.	-2.689175	1.017895	-0.274334
16.	8.	0.	-2.085825	2.252392	0.054109
17.	6.	0.	-4.025646	1.103173	-1.050123
18.	7.	0.	-4.776770	0.038209	-0.603267
19.	6.	0.	-4.453535	-0.454915	0.721963
20.	6.	0.	-3.114751	0.285912	1.020774
21.	6.	0.	6.913091	0.425274	0.056950
22.	6.	0.	3.575182	-2.853123	-1.886478

23.	1.	0.	2.974316	-0.169148	-1.578021
24.	1.	0.	2.125512	1.682181	-0.784598
25.	8.	0.	1.324965	3.642349	0.252195
26.	6.	0.	-0.897888	3.442882	-1.685551
27.	1.	0.	0.134772	1.108719	-2.046457
28.	8.	0.	-1.947623	-0.455444	-2.094975
29.	8.	0.	-4.310831	1.879913	-1.936680
30.	8.	0.	-5.514992	0.021432	1.558228
31.	6.	0.	-0.788691	-2.607216	1.614512
32.	6.	0.	-2.170701	-2.638918	1.783166
33.	6.	0.	-3.033688	-2.610787	0.673387
34.	6.	0.	-2.478307	-2.838875	-0.587863
35.	6.	0.	-1.094140	-2.844251	-0.767978
36.	6.	0.	-0.259873	-2.575367	0.318295
37.	6.	0.	2.896585	-0.393290	1.903429
38.	6.	0.	1.839019	2.613234	2.447621
39.	1.	0.	0.140503	0.036411	0.818076
40.	8.	0.	1.061416	-2.218092	0.159695
41.	6.	0.	-4.420162	-2.037031	0.824670
42.	1.	0.	5.562005	-0.714318	1.258097
43.	1.	0.	5.341131	-0.805833	-1.783940
44.	1.	0.	5.988716	-2.064673	-0.743936
45.	1.	0.	3.744795	-2.488434	0.213156
46.	1.	0.	4.458053	1.453128	-0.596669
47.	1.	0.	4.651454	1.578909	1.146920
48.	1.	0.	0.939977	-1.273912	-1.687211
49.	1.	0.	-0.511944	3.541129	1.327980
50.	1.	0.	-5.726172	-0.059340	-0.942173
51.	1.	0.	-3.336352	1.081741	1.736309
52.	1.	0.	-2.338136	-0.349835	1.434233
53.	1.	0.	6.951915	0.949755	-0.905371
54.	1.	0.	7.150675	1.152463	0.841052
55.	1.	0.	7.703681	-0.332903	0.053694
56.	1.	0.	2.610005	-3.358051	-1.790945
57.	1.	0.	4.352566	-3.623520	-1.931260
58.	1.	0.	3.589419	-2.315532	-2.842807
59.	1.	0.	-1.582752	3.055931	-2.445932
60.	1.	0.	-1.315573	4.378090	-1.303072
61.	1.	0.	0.075685	3.654426	-2.135289
62.	1.	0.	-5.386711	-0.340733	2.445441
63.	1.	0.	-0.119157	-2.512913	2.463505

64.	1.	0.	-2.582821	-2.554161	2.786475
65.	1.	0.	-3.121585	-2.881337	-1.460685
66.	1.	0.	-0.682581	-2.911904	-1.768208
67.	1.	0.	3.247472	0.286665	2.683763
68.	1.	0.	1.869216	-0.679077	2.135756
69.	1.	0.	3.496743	-1.302115	1.976753
70.	1.	0.	2.929313	2.679882	2.508859
71.	1.	0.	1.511013	1.794309	3.093939
72.	1.	0.	1.421337	3.546931	2.832574
73.	1.	0.	-5.116825	-2.431475	0.077964
74.	1.	0.	-4.826443	-2.303582	1.807334

Table S7. Experimental and calculated ^{13}C NMR data of pyrrospirone J (**10**)

Experimental data (ppm)				Calculated data (ppm)			
No.	δ_C	No.	δ_C	No.	δ_C	No.	δ_C
1	47.3	16	172.1	1	46.8	16	168.4
2	27.4	17	86.2	2	28.4	17	86.0
3	45.5	18	40.9	3	43.5	18	44.3
4	26.8	19	45.0	4	29.2	19	43.6
5	59.5	20	129.4	5	60.6	20	126.5
6	42.5	21	133.3	6	46.1	21	129.3
7	49.9	22	124.4	7	52.9	22	125.4
8	39.2	23	158.7	8	42.5	23	157.3
9	85.0	24	119.7	9	86.4	24	118.5
10	58.8	25	130.6	10	60.4	25	132.8
11	63.5	26	22.7	11	62.8	26	22.8
12	81.3	27	19.7	12	81.0	27	18.7
13	44.5	28	15.2	13	47.7	28	15.7
14	**180.9**	29	21.0	14	**184.1**	29	21.1
15	79.7	30	26.0	15	77.9	30	24.4
CAME	0.28						
LAD	3.6						

CMAE = corrected mean absolute error, computed as $(1/n)\sum_{t}^{n}|\delta\text{calcd}-\delta\text{exptl}|$, where δ refers to the scaled calculated chemical shifts here. LAD= largest absolute deviation.

Figure S46. Four conformations of the low-energy conformers of pyrrospirone J (**10**) calculated at B3LYP/6-31G(d) level

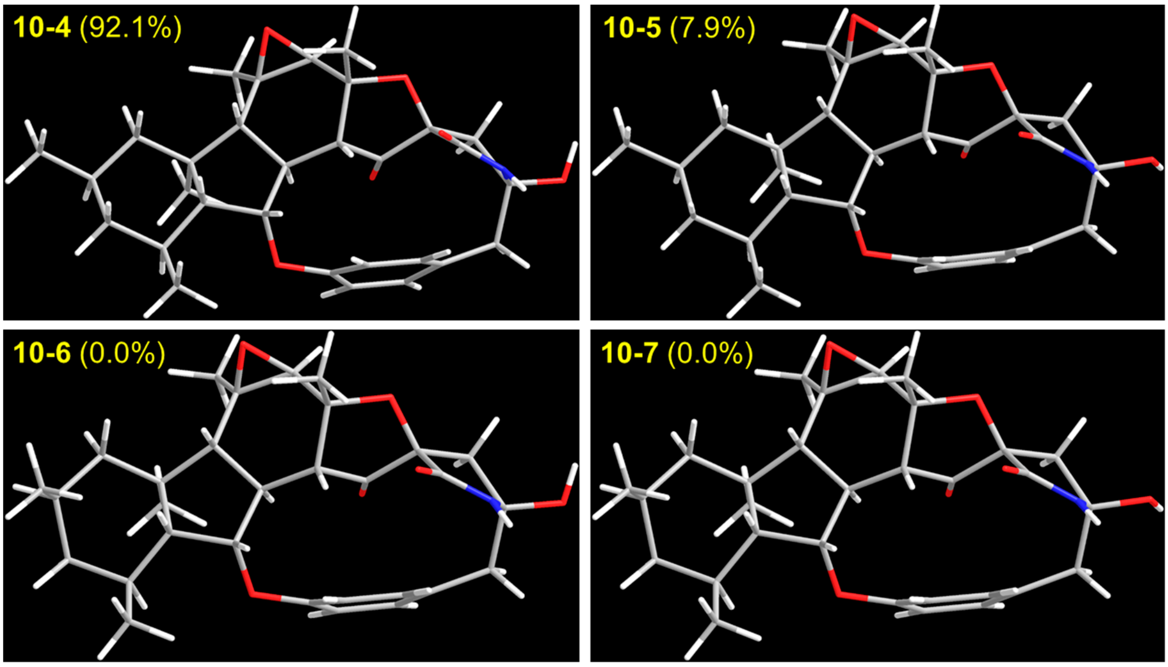

Table S8. Gibbs free energies[a] and equilibrium populations[b] of the low-energy conformers of pyrrospirone J (**10**) for ^{13}C NMR calculation

Conformers	In DMSO	
	Δ*G*	*P* (%)
**10-4**	0.00	92.1
**10-5**	1.46	7.9
**10-6**	8.04	0
**10-7**	9.54	0

[a] B3LYP/6-31+G(d,p), in kcal/mol; [b] from ΔG values at 298.15K.

Table S9. Cartesian coordinates for the low-energy reoptimized MMFF conformers of pyrrospirone J (**10**) at B3LYP/6-311+G(d,p) level of theory in DMSO for ^{13}C NMR calculation

**10-4**		Standard Orientation (Ångstroms)			
Center number	Atomic number	Atom type	X	Y	Z
1.	6.	0.	-5.647761	-0.169344	-0.162415
2.	6.	0.	-5.308569	-1.400759	0.705858
3.	6.	0.	-3.905675	-2.015209	0.480091
4.	6.	0.	-2.888432	-0.871359	0.567028
5.	6.	0.	-3.141882	0.305471	-0.438567
6.	6.	0.	-4.527376	0.896280	-0.112883
7.	6.	0.	-1.363109	-1.138893	0.577618
8.	6.	0.	-0.777670	0.167813	-0.013617
9.	6.	0.	-1.918358	1.204497	-0.038559
10.	6.	0.	0.537903	0.668317	0.568070
11.	6.	0.	0.782838	2.210397	0.678760
12.	6.	0.	-0.061820	2.944917	-0.374887
13.	6.	0.	-1.414651	2.461710	-0.781216

14.	6.	0.	1.659620	0.410272	-0.409038
15.	6.	0.	2.887894	1.119211	0.143033
16.	6.	0.	4.062938	1.000760	-0.832452
17.	6.	0.	4.903722	-0.201238	-0.283777
18.	7.	0.	4.648442	-0.057293	1.148454
19.	6.	0.	3.468965	0.559018	1.464487
20.	6.	0.	-6.996588	0.434681	0.251394
21.	6.	0.	-3.631830	-3.126011	1.504048
22.	6.	0.	-3.111018	-0.171460	-1.909828
23.	6.	0.	0.390229	-2.500844	-0.289050
24.	6.	0.	0.979294	-2.650407	-1.548797
25.	6.	0.	2.360796	-2.496438	-1.695046
26.	6.	0.	3.188563	-2.200683	-0.601597
27.	6.	0.	2.607385	-2.317278	0.671748
28.	6.	0.	1.228014	-2.468118	0.832700
29.	8.	0.	-0.979287	-2.315467	-0.196726
30.	6.	0.	-1.908518	2.818215	-2.166972
31.	8.	0.	2.980755	0.638428	2.579946
32.	8.	0.	1.529196	0.127477	-1.578061
33.	8.	0.	6.281826	-0.060034	-0.563752
34.	6.	0.	0.600383	2.793853	2.073537
35.	6.	0.	4.595367	-1.656888	-0.797389
36.	1.	0.	0.774632	0.225040	1.537412
37.	1.	0.	-2.123852	1.548806	0.983458
38.	1.	0.	-3.057154	-0.418228	1.558379
39.	1.	0.	-0.566236	-0.059598	-1.061985
40.	8.	0.	-1.245674	3.613475	0.078594
41.	8.	0.	2.239982	2.391761	0.319719
42.	1.	0.	-5.749552	-0.507415	-1.203106
43.	1.	0.	-5.379277	-1.106960	1.765844
44.	1.	0.	-6.075932	-2.172347	0.554296
45.	1.	0.	-3.863191	-2.467390	-0.518862
46.	1.	0.	-4.501877	1.336846	0.895539
47.	1.	0.	-4.773736	1.715895	-0.802103
48.	1.	0.	-1.030776	-1.300978	1.608550
49.	1.	0.	0.531738	3.512787	-1.093345
50.	1.	0.	3.729192	0.852679	-1.860006
51.	1.	0.	4.689263	1.898599	-0.784169
52.	1.	0.	5.110194	-0.648457	1.830272
53.	1.	0.	-6.964683	0.796681	1.287231
54.	1.	0.	-7.802277	-0.306121	0.182862
55.	1.	0.	-7.266418	1.283020	-0.389118
56.	1.	0.	-3.625303	-2.727404	2.527544
57.	1.	0.	-2.667545	-3.607011	1.315787
58.	1.	0.	-4.410885	-3.896418	1.456887
59.	1.	0.	-3.403307	0.633901	-2.589318
60.	1.	0.	-2.129515	-0.538639	-2.219253
61.	1.	0.	-3.813007	-0.992472	-2.072600
62.	1.	0.	0.341292	-2.747528	-2.421641
63.	1.	0.	2.776886	-2.480512	-2.699304
64.	1.	0.	3.217572	-2.226788	1.564660
65.	1.	0.	0.807004	-2.473450	1.832962
66.	1.	0.	-2.996150	2.948842	-2.178297
67.	1.	0.	-1.647054	2.045953	-2.896717

68.	1.	0.	-1.452088	3.760856	-2.483340
69.	1.	0.	6.556475	0.815653	-0.244414
70.	1.	0.	-0.442487	2.714548	2.392141
71.	1.	0.	0.865798	3.856192	2.063967
72.	1.	0.	1.248962	2.267098	2.775152
73.	1.	0.	5.338231	-2.283885	-0.285944
74.	1.	0.	4.857647	-1.678741	-1.860054

**10-5**		Standard Orientation (Ångstroms)			
Center number	Atom number	Atom type	X	Y	Z
1.	6.	0.	-5.647713	-0.174103	-0.162189
2.	6.	0.	-5.307190	-1.403798	0.707956
3.	6.	0.	-3.903827	-2.017293	0.482649
4.	6.	0.	-2.887567	-0.872365	0.567697
5.	6.	0.	-3.142271	0.302897	-0.439313
6.	6.	0.	-4.528373	0.892699	-0.114346
7.	6.	0.	-1.362023	-1.138196	0.578175
8.	6.	0.	-0.777535	0.168405	-0.014316
9.	6.	0.	-1.919744	1.203553	-0.040442
10.	6.	0.	0.537445	0.671305	0.566691
11.	6.	0.	0.780273	2.214195	0.675873
12.	6.	0.	-0.066245	2.946519	-0.377883
13.	6.	0.	-1.418136	2.460875	-0.784148
14.	6.	0.	1.660502	0.413894	-0.409218
15.	6.	0.	2.886298	1.126177	0.141003
16.	6.	0.	4.065121	1.009940	-0.829893
17.	6.	0.	4.901683	-0.191117	-0.271118
18.	7.	0.	4.636930	-0.060819	1.144862
19.	6.	0.	3.471259	0.572583	1.464530
20.	6.	0.	-6.997110	0.429208	0.250717
21.	6.	0.	-3.628833	-3.126564	1.507946
22.	6.	0.	-3.111062	-0.175875	-1.909983
23.	6.	0.	0.390918	-2.501582	-0.287375
24.	6.	0.	0.979875	-2.652947	-1.546971
25.	6.	0.	2.361339	-2.500235	-1.692837
26.	6.	0.	3.188524	-2.205425	-0.598938
27.	6.	0.	2.607615	-2.319199	0.674837
28.	6.	0.	1.228361	-2.468788	0.834850
29.	8.	0.	-0.977874	-2.315401	-0.195941
30.	6.	0.	-1.912709	2.815551	-2.170183
31.	8.	0.	2.990550	0.662712	2.582505
32.	8.	0.	1.530850	0.124894	-1.577373
33.	8.	0.	6.297158	0.029169	-0.440403
34.	6.	0.	0.597288	2.798667	2.070242
35.	6.	0.	4.594336	-1.655530	-0.787514
36.	1.	0.	0.774849	0.229604	1.536759
37.	1.	0.	-2.125470	1.548623	0.981255
38.	1.	0.	-3.056317	-0.418090	1.558509
39.	1.	0.	-0.565620	-0.059964	-1.062466
40.	8.	0.	-1.251552	3.613314	0.074831
41.	8.	0.	2.235343	2.397661	0.314968
42.	1.	0.	-5.749214	-0.513964	-1.202345
43.	1.	0.	-5.377827	-1.108392	1.767495
44.	1.	0.	-6.073901	-2.176340	0.557877
45.	1.	0.	-3.861339	-2.470686	-0.515781

46.	1.	0.	-4.503201	1.334809	0.893393
47.	1.	0.	-4.775622	1.711057	-0.804731
48.	1.	0.	-1.029190	-1.299839	1.608971
49.	1.	0.	0.526430	3.515240	-1.096374
50.	1.	0.	3.728782	0.864953	-1.857980
51.	1.	0.	4.709212	1.890797	-0.766242
52.	1.	0.	5.150170	-0.591714	1.837902
53.	1.	0.	-6.965535	0.792884	1.285970
54.	1.	0.	-7.802067	-0.312507	0.183378
55.	1.	0.	-7.267777	1.276284	-0.391104
56.	1.	0.	-3.622476	-2.726657	2.530919
57.	1.	0.	-2.664162	-3.607006	1.320220
58.	1.	0.	-4.407229	-3.897710	1.461888
59.	1.	0.	-3.404108	0.628387	-2.590416
60.	1.	0.	-2.129127	-0.542367	-2.218867
61.	1.	0.	-3.812289	-0.997767	-2.071732
62.	1.	0.	0.341855	-2.749853	-2.419777
63.	1.	0.	2.777086	-2.485390	-2.697460
64.	1.	0.	3.218243	-2.224588	1.566805
65.	1.	0.	0.806736	-2.472242	1.834814
66.	1.	0.	-3.000600	2.944134	-2.181689
67.	1.	0.	-1.649738	2.043414	-2.899565
68.	1.	0.	-1.458037	3.758899	-2.486945
69.	1.	0.	6.520756	-0.181848	-1.360968
70.	1.	0.	-0.445218	2.717589	2.389799
71.	1.	0.	0.860783	3.861457	2.059079
72.	1.	0.	1.248187	2.274496	2.771578
73.	1.	0.	5.335727	-2.278612	-0.268898
74.	1.	0.	4.849956	-1.690858	-1.854976

**10-6**		Standard Orientation (Ångstroms)			
Center number	Atom number	Atom type	X	Y	Z
1.	6.	0.	-5.641922	0.000002	0.200826
2.	6.	0.	-5.343885	-1.481022	-0.151091
3.	6.	0.	-3.915349	-2.010913	0.200430
4.	6.	0.	-2.958463	-0.829251	0.406878
5.	6.	0.	-3.163666	0.353185	-0.597312
6.	6.	0.	-4.544500	0.997248	-0.311581
7.	6.	0.	-1.432077	-1.095061	0.498603
8.	6.	0.	-0.808673	0.185286	-0.107607
9.	6.	0.	-1.933559	1.233962	-0.174378
10.	6.	0.	0.495382	0.678565	0.506956
11.	6.	0.	0.756822	2.218479	0.610069
12.	6.	0.	-0.052151	2.955930	-0.468216
13.	6.	0.	-1.398627	2.484630	-0.907655
14.	6.	0.	1.645195	0.390131	-0.426919
15.	6.	0.	2.863172	1.095625	0.153300
16.	6.	0.	4.067698	0.944582	-0.781288
17.	6.	0.	4.877656	-0.253739	-0.180074
18.	7.	0.	4.581639	-0.072591	1.239932
19.	6.	0.	3.396115	0.556171	1.503986
20.	6.	0.	-5.939978	0.197698	1.699139
21.	6.	0.	-3.918116	-2.950783	1.416243
22.	6.	0.	-3.103551	-0.137602	-2.062739
23.	6.	0.	0.339713	-2.501704	-0.260611

24.	6.	0.	0.963535	-2.690623	-1.498152
25.	6.	0.	2.350279	-2.555660	-1.607197
26.	6.	0.	3.148838	-2.241375	-0.497370
27.	6.	0.	2.529171	-2.318547	0.760887
28.	6.	0.	1.143857	-2.448528	0.884452
29.	8.	0.	-1.029455	-2.302905	-0.213887
30.	6.	0.	-1.852303	2.849753	-2.305345
31.	8.	0.	2.872331	0.659749	2.601177
32.	8.	0.	1.551749	0.081507	-1.592835
33.	8.	0.	6.264821	-0.134638	-0.422319
34.	6.	0.	0.544680	2.817416	1.994511
35.	6.	0.	4.567123	-1.718687	-0.665630
36.	1.	0.	0.695812	0.244414	1.488467
37.	1.	0.	-2.159627	1.585119	0.840922
38.	1.	0.	-3.195908	-0.400587	1.391331
39.	1.	0.	-0.572969	-0.065577	-1.145862
40.	8.	0.	-1.240577	3.636767	-0.045704
41.	8.	0.	2.224482	2.378392	0.285666
42.	1.	0.	-6.574361	0.249858	-0.322635
43.	1.	0.	-6.095079	-2.109966	0.344375
44.	1.	0.	-5.519445	-1.615020	-1.224886
45.	1.	0.	-3.552013	-2.596022	-0.652129
46.	1.	0.	-4.423136	1.790134	0.437942
47.	1.	0.	-4.902525	1.498269	-1.219688
48.	1.	0.	-1.145866	-1.221505	1.548619
49.	1.	0.	0.564898	3.516520	-1.172467
50.	1.	0.	3.765508	0.777405	-1.815649
51.	1.	0.	4.702064	1.836687	-0.732117
52.	1.	0.	5.014770	-0.654080	1.948396
53.	1.	0.	-5.069386	-0.004893	2.333065
54.	1.	0.	-6.748226	-0.467088	2.027580
55.	1.	0.	-6.254819	1.229182	1.898452
56.	1.	0.	-4.257908	-2.436652	2.324263
57.	1.	0.	-2.915972	-3.351390	1.604602
58.	1.	0.	-4.589045	-3.801493	1.247507
59.	1.	0.	-3.254485	0.688162	-2.761905
60.	1.	0.	-2.158591	-0.628627	-2.311035
61.	1.	0.	-3.896127	-0.863000	-2.260563
62.	1.	0.	0.350242	-2.803798	-2.386608
63.	1.	0.	2.796123	-2.569907	-2.598618
64.	1.	0.	3.113675	-2.212084	1.668982
65.	1.	0.	0.692409	-2.422293	1.871126
66.	1.	0.	-2.939443	2.978304	-2.348992
67.	1.	0.	-1.565333	2.085712	-3.034338
68.	1.	0.	-1.388733	3.796400	-2.598552
69.	1.	0.	6.540455	0.745114	-0.115230
70.	1.	0.	-0.507683	2.755234	2.284276
71.	1.	0.	0.824369	3.876093	1.982047
72.	1.	0.	1.165998	2.289537	2.719271
73.	1.	0.	5.287027	-2.340076	-0.115935
74.	1.	0.	4.860539	-1.771935	-1.719008

**10-7**		Standard Orientation (Ångstroms)			
Center number	Atom number	Atom type	X	Y	Z
1.	6.	0.	-5.641976	-0.004192	0.200656

2.	6.	0.	-5.342518	-1.485351	-0.149445
3.	6.	0.	-3.913563	-2.013465	0.202984
4.	6.	0.	-2.957653	-0.830621	0.407509
5.	6.	0.	-3.164085	0.350291	-0.598056
6.	6.	0.	-4.545437	0.993447	-0.312878
7.	6.	0.	-1.431041	-1.094703	0.499109
8.	6.	0.	-0.808505	0.185491	-0.108365
9.	6.	0.	-1.934847	1.232657	-0.176366
10.	6.	0.	0.494877	0.681259	0.505663
11.	6.	0.	0.753980	2.221976	0.607494
12.	6.	0.	-0.056551	2.957211	-0.471184
13.	6.	0.	-1.401973	2.483412	-0.910846
14.	6.	0.	1.646171	0.393875	-0.426947
15.	6.	0.	2.861601	1.102753	0.151735
16.	6.	0.	4.069917	0.954723	-0.778133
17.	6.	0.	4.875468	-0.242563	-0.167715
18.	7.	0.	4.570505	-0.075834	1.236193
19.	6.	0.	3.398421	0.569225	1.504282
20.	6.	0.	-5.940091	0.194951	1.698758
21.	6.	0.	-3.915576	-2.951342	1.420337
22.	6.	0.	-3.103729	-0.142263	-2.062864
23.	6.	0.	0.340465	-2.502570	-0.259102
24.	6.	0.	0.964148	-2.692982	-1.496524
25.	6.	0.	2.350792	-2.558931	-1.605289
26.	6.	0.	3.148825	-2.245516	-0.495087
27.	6.	0.	2.529556	-2.320265	0.763580
28.	6.	0.	1.144441	-2.449365	0.886317
29.	8.	0.	-1.027933	-2.303216	-0.213162
30.	6.	0.	-1.855927	2.846321	-2.309058
31.	8.	0.	2.881931	0.682653	2.604065
32.	8.	0.	1.553688	0.078997	-1.591815
33.	8.	0.	6.277643	-0.041939	-0.300958
34.	6.	0.	0.540851	2.821737	1.991504
35.	6.	0.	4.565825	-1.716106	-0.656582
36.	1.	0.	0.695994	0.248579	1.487850
37.	1.	0.	-2.161067	1.584638	0.838584
38.	1.	0.	-3.195118	-0.400894	1.391491
39.	1.	0.	-0.572227	-0.066309	-1.146338
40.	8.	0.	-1.246537	3.636368	-0.049915
41.	8.	0.	2.219794	2.384202	0.281719
42.	1.	0.	-6.574645	0.244136	-0.323129
43.	1.	0.	-6.093219	-2.114406	0.346636
44.	1.	0.	-5.517714	-1.620827	-1.223121
45.	1.	0.	-3.549812	-2.599657	-0.648680
46.	1.	0.	-4.424599	1.787144	0.435839
47.	1.	0.	-4.903994	1.493319	-1.221411
48.	1.	0.	-1.144286	-1.220767	1.548962
49.	1.	0.	0.559794	3.518607	-1.175391
50.	1.	0.	3.765362	0.791356	-1.813347
51.	1.	0.	4.721140	1.830139	-0.712027
52.	1.	0.	5.054653	-0.598597	1.955896
53.	1.	0.	-5.069237	-0.006240	2.332769
54.	1.	0.	-6.747779	-0.470090	2.028074
55.	1.	0.	-6.255679	1.226407	1.896974
56.	1.	0.	-4.255771	-2.435998	2.327507

57.	1.	0.	-2.913117	-3.350827	1.609504
58.	1.	0.	-4.585810	-3.802891	1.253039
59.	1.	0.	-3.254934	0.682650	-2.762965
60.	1.	0.	-2.158425	-0.633027	-2.310416
61.	1.	0.	-3.895950	-0.868303	-2.259909
62.	1.	0.	0.350788	-2.805970	-2.384912
63.	1.	0.	2.796149	-2.573974	-2.597127
64.	1.	0.	3.114607	-2.209742	1.670621
65.	1.	0.	0.692569	-2.421563	1.872709
66.	1.	0.	-2.943286	2.973010	-2.353248
67.	1.	0.	-1.567426	2.082121	-3.037335
68.	1.	0.	-1.393863	3.793494	-2.602924
69.	1.	0.	6.525084	-0.274872	-1.210068
70.	1.	0.	-0.511235	2.757550	2.282019
71.	1.	0.	0.818536	3.880913	1.977590
72.	1.	0.	1.164500	2.296392	2.716059
73.	1.	0.	5.284522	-2.333656	-0.100736
74.	1.	0.	4.852082	-1.782075	-1.714775
